# Safety, Humoral and Cell Mediated Immune Responses to Two Formulations of an Inactivated, Split-Virion Influenza A/H5N1 Vaccine in Children

**DOI:** 10.1371/journal.pone.0004028

**Published:** 2008-12-29

**Authors:** Tawee Chotpitayasunondh, Usa Thisyakorn, Chitsanu Pancharoen, Stephanie Pepin, Nolwenn Nougarede

**Affiliations:** 1 Queen Sirikit National Institute of Child Health, Bangkok, Thailand; 2 Chulalongkorn Hospital, Bangkok, Thailand; 3 sanofi pasteur, Marcy l'Etoile, France; Institut Pasteur, France

## Abstract

**Background:**

Highly pathogenic influenza A/H5N1 has caused outbreaks in wild birds and poultry in Asia, Africa and Europe. It has also infected people, especially children, causing severe illness and death. Although the virus shows limited ability to transmit between humans, A/H5N1 represents a potential source of the next influenza pandemic. This study assesses the safety and immunogenicity of aluminium hydroxide adjuvanted (Al) and non adjuvanted influenza A/Vietnam/1194/2004 NIBRG-14 (H5N1) vaccine in children.

**Methods and Findings:**

In a Phase II, open, randomised, multicentre trial 180 children aged 6 months to 17 years received two injections, 21 days apart, of vaccine containing either: 30 µg haemagglutinin (HA) with adjuvant (30 µg+Al) or 7.5 µg HA without adjuvant. An additional 60 children aged 6–35 months received two “half dose” injections (ie 15 µg+Al or 3.8 µg). Safety was followed for 21 days after vaccination. Antibody responses were assessed 21 days after each injection and cellular immune responses were explored. Vaccination appeared well tolerated in all age groups. The 30 µg+Al formulation was more immunogenic than 7.5 µg in all age groups: in these two groups 79% and 46% had haemagglutinination inhibition antibody titres ≥32 (1/dil). Among 6–35 month-olds, the full doses were more immunogenic than their half dose equivalents. Vaccination induced a predominantly Th2 response against H5 HA.

**Conclusions:**

This influenza A(H5N1) vaccine was well tolerated and immunogenic in children and infants, with Al adjuvant providing a clear immunogenic advantage. These results demonstrate that an H5N1 Al-adjuvanted vaccine, previously shown to be immunogenic and safe in adults, can also be used in children, the group most at risk for pandemic influenza.

**Trial Registration:**

ClinicalTrials.gov NCT00491985

## Introduction

The emergence of a novel influenza virus, against which the bulk of the world's population has no immunity, presents a significant pandemic risk. Highly pathogenic avian influenza A/H5N1 viruses have rapidly expanded their geographical range, with infected birds identified across Asia, Africa, the Middle East and Europe [1]. As of June 2008, 382 cases of human infection by H5N1 had been confirmed, of which 46% (179 cases) were aged 0–19 years, and 63% (241 cases) were recorded as fatal [2]. Although current highly pathogenic H5N1 strains do not meet all the criteria for a pandemic virus [3] since they appear poorly able to spread from person-to-person, a probable case of human-to-human transmission has been recorded [4]. Each case of human infection by this subtype presents the potential for the virus to acquire the ability to transmit more effectively from person-to-person. Avian H5N1 therefore represents a potential source of the next influenza pandemic [5]. It has been estimated that a severe pandemic in the United States could infect 200 million people, resulting in clinical illness in 90 million and death in 2 million [6]. The same study predicted that as a consequence of illness within the working population, gross domestic product could decrease by 5% and the financial burden of providing outpatient care for 18 to 45 million people could total $675 billion.

During an influenza pandemic, children are expected to be severely affected. Given reports of mortality rates of close to 90% in children infected with avian A/H5N1 strains in Thailand, the evaluation of human H5N1 vaccines in young people is therefore crucial [7]. As well as being at high risk of contracting influenza, children are key in viral transmission: they shed influenza virus more efficiently and for longer than adults and tend to have extensive social networks [8].

Preparation for an influenza pandemic includes stock-piling of antivirals and the development of candidate vaccines. Conventional influenza vaccines may not be suitable against a pandemic caused by influenza strain such as H5N1 due to the lack of pre-existing immunity in the human population against any newly emerged strain, and also the low immunogenicity of H5N1 strains in particular [9]. Alternative methods for rapid production and dose-reduction of vaccines are desirable since an immunologically naïve population will require at least two doses of an H5N1 vaccine and the global response to an influenza pandemic will require the maximum number of vaccine doses in the shortest possible time after the onset of the pandemic[3], [10]–[12].

The aim of the present study was to document in Thai children the safety and immunogenicity of an H5N1 influenza vaccine based on a reference strain derived by the UK National Institute for Biological Standards and Control (NIBSC) from the pathogenic influenza A Vietnam/1194/2004 strain. This vaccine has been shown to be safe, immunogenic and able to induce cross-reactive immune response in adult volunteers [13]. Here, we present data from the first part (up to day 42) of a continuing trial. This trial also explored the Th1/Th2 balance of cellular immune responses before and after vaccination in infants and young children.

## Methods

This multicentre, randomised, open Phase II trial evaluated the safety and humoral immunogenicity of different formulations of influenza A/Vietnam/1194/2004 NIBRG-14 (H5N1) vaccine and explored the cellular immune responses to the vaccine in Thai children.

The study was conducted in accordance with all relevant regulations and Good Clinical Practice (GCP) guidelines.

The study protocol was approved by the independent ethics committees of Chulalongkorn Hospital and the Queen Sirikit National Institute of Child Health (Children's Hospital) prior to the start of the trial. The clinical trial protocol and the supporting CONSORT checklist are available as supporting information; see Checklist S1 and Protocol S1.

### Participants

Healthy children, aged 6 months to 17 years, were recruited at two centres in Bangkok, Thailand between June and September 2007. The main exclusion criteria were: ongoing febrile illness; recent receipt (preceding three months) of blood or blood-derived products; seropositivity for Hepatitis B, C, or HIV; history of H5N1 infection or previous vaccination with an avian influenza vaccine; any vaccination during the previous four weeks, or planned in the following four weeks; congenital or acquired immunodeficiency; immunosuppressive therapy within the preceding six months; long-term systemic corticosteroid therapy; systemic hypersensitivity to any of the vaccine components or a history of life-threatening reaction to vaccines containing the same substances; pregnancy; or chronic illness at a stage which could interfere with trial conduct or completion.

Before enrolment, each child's parents (or other legal representative) provided their written informed consent and each child aged 7 years or older provided written informed assent.

### Vaccine

The H5N1 vaccine was a monovalent A/H5N1, inactivated, split virion-influenza virus vaccine (Sanofi Pasteur, Lyon, France). It was propagated in embryonated hens' eggs, using the licensed manufacturing process for the interpandemic vaccine Vaxigrip®, as described previously [13]. The vaccine strain was the influenza A/Vietnam/1194/NIBRG-14 (H5N1) reference strain prepared by the NIBSC, and is one of the reference viruses indicated as suitable for use in a mock-up vaccine by the European Medicines Agency (EMEA) [14].

Based on the results from a previous trial in French adults [13], two formulations were selected for investigation in groups of children of all ages: 30 µg HA with aluminium hydroxide adjuvant (30 µg+Al), and 7.5 µg HA without adjuvant (7.5 µg). Furthermore, in a subgroup of the youngest age group (aged 6 to 35 months), two “half-dose” formulations were evaluated, i.e., 15 µg+Al and 3.75 µg.

Vaccine was presented in ready-to-use multi-dose vials. Adjuvanted vaccine vials contained per millilitre, 60 µg HA and 1200 µg aluminium hydroxide adjuvant, expressed as Al^3+^. The volume of vaccine withdrawn for injection of the full or half doses was respectively, 0.5 ml or 0.25 ml. Non-adjuvanted vaccine vials contained 12.5 µg HA/ml, and 0.6 or 0.3 ml were withdrawn for injection.

### Procedures

As a precaution, children were enrolled and vaccinated in an age-based step-down design. In the first step, 60 children aged 9–17 years were enrolled and randomised to receive one of the two full-dose formulations. Safety data from the first seven days after the first vaccination were reviewed before deciding whether to proceed to the second vaccination of these first 60 children, and to enrol and vaccinate the next 60 children, aged 3–8 years. The same procedure was followed before enrolling and vaccinating 60 children aged 6–35 months with the half dose vaccines, and again before enrolling and vaccinating the final 60 children aged 6–35 months with the full dose vaccines. The randomisation list was generated by the sponsor's biostatistics department using the block permutation method, stratified by age group and centre. Randomization lists for each age group and centre featured a list of sequentially assigned subject number and, concealed underneath a scratchable patch, the corresponding vaccine assigned assigned to that subject number. At each centre, the enrolling investigator enrolled the subject, assigned the next non-assigned subject number on the list, then scratched the patch to reveal the assigned treatment group.

All subjects received two intramuscular injections, 21 days apart, of the assigned formulation in the deltoid (children aged ≥12 months) or anterolateral aspect of the thigh (<12 months) and were kept under observation for 30 minutes each time. Blood samples, collected before and 21 days after vaccination, were processed at the trial centres and serum samples were stored and shipped frozen at −17°C to the Global Clinical Immunology Laboratory (sanofi pasteur, Pennsylvania, USA) for analysis. Additional blood samples were collected in sodium heparin tubes before the first vaccination, and 8 days after the second vaccination from the 120 children aged 6–35 months and peripheral blood mononuclear cells were isolated for cellular immune response testing.

### Safety analysis

Parents were given safety diaries, thermometers and rulers to record any adverse events occurring up to day 21 after vaccination. The daily occurrence of a set of solicited systemic and injection site reactions were recorded up to day 7 following vaccination (see results for details). The severity of non-measurable reactions was assessed using a grading scale of 1 to 3. During the following visit, investigators interviewed the children and their parents, transcribed events into case report forms, and assessed whether they were vaccine-related. The list of solicited reactions was adapted to be suitable for infants, and therefore differed between children <2 years old and older children (see results for details).

### Antibody response

Serum samples were tested for their ability to inhibit haemagglutination and to neutralise influenza A/H5N1 virus. The haemagglutination inhibition assay reflects the ability of specific anti-influenza antibodies to inhibit haemagglutination of horse red blood cells by influenza virus HA, and has been described previously [13]. The starting dilution of the HI assay used here was 1∶8, and seroresponse threshold was 1∶32. Titres were expressed as the reciprocal of dilution (1/dil). Samples without detectable antibody activity were assigned the titre of half the assay detection limit, i.e. a titre of 1∶4.

Neutralising antibody activity was measured using a seroneutralisation (SN) assay based on the ability of antibodies to inhibit the infection of Madin-Darby canine kidney (MDCK) cell culture by influenza virus. Inactivated human serum samples were pre-incubated with a standardised amount of virus prior to the addition of MDCK cells. After overnight incubation, enzyme-linked immunosorbent assay (ELISA) was used to measure the viral nucleoprotein in infected MDCK cells. Since serum antibodies to the influenza virus HA inhibit the viral infection of MDCK cells, the optical density results of the ELISA were inversely proportional to the serum Ab concentration. Samples without detectable antibody activity were assigned the titre of half the assay's detection limit (10), i.e. a titre of 5.

### Cell Mediated Immunity

Th1 and Th2 cytokine secretion was assessed on freshly isolated peripheral blood mononuclear cells (PMBCs) after 4 days of in vitro re-stimulation with recombinant haemagglutinin (rHA) analogous to that of Influenza A/Vietnam/1203/2004(H5N1) (Protein Science Corporation, Meriden, CT) or A/New Caledonia/20/99(H1N1) split inactivated vaccine. Cytokine secretion (interleukin (IL) 5 and IL13 as surrogate markers of a Th2 response, and interferon gamma (IFNγ), and tumor necrosis factor alpha (TNFα) as markers of a Th1 response) was quantified in the cell supernatant by Luminex technology. Briefly, PBMCs were stimulated in vitro in 96-well plate with 0.1 µg/ml of rHA. Secreted cytokines were measured by Luminex using Human cytokine 4-plex and Bio-Plex reagent kits (Bio-Rad, Hercules, CA) according to the manufacturer's instruction. Premixed anti-cytokine biotinylated antibody capture beads were dispensed in a pre-moistened filter plate. The beads were washed standard, control and samples were dispensed into the plate. After 30 minutes at room temperature, cells were washed before biotinylated detection antibody solution was added to each well. Plates were incubated for a further 30 minutes as above. After several washes, streptavidin-PE were dispensed into each well of the plate and incubated as above for 10 more minutes. Data were acquired with the Bio-plex Luminex 100 (Bio-Rad, Hercules, CA) then analyzed with the Bio-plex manager software.

### Statistical Analysis

The study cohort (N = 240) provided a probability >90% of detecting an adverse event with an incidence of 1% in the study, and a probability of 70% for each pooled adjuvanted and non-adjuvanted group (N = 120). Statistical analyses were descriptive with no hypothesis testing, and were performed on the full analysis set. In line with EMEA immunogenicity criteria [14], [15], HI titres were described per group using i) geometric mean titre (GMT) at each timepoint, ii) the geometric mean ratio of titres between pre- and post-vaccination (GMTR), iii) the proportion of subjects with titres ≥32, and, referred to hereafter as the seroresponse rate iv) the proportion of subjects with either a pre-vaccination titre of <8 and a post-vaccination titre of ≥32, or a 4-fold rise in titre from a pre-vaccination titre of ≥8, referred to as hereafter as the seroconversion rate. In a population that is naïve before vaccination, the above defined seroresponse and seroconversion rates are identical, and the GMTR is equal the GMT divided by the half the assay's lower detection limit (i.e., 4). Neutralising titres were described per group as GMT and as the proportion of each group with 2- or 4-rise in titre after vaccination. Analyses of cell mediated responses were descriptive.

## Results

Between June and September 2007, 240 healthy children, aged between 6 months and 17 years, were recruited and vaccinated as planned. Of these, 239 successfully completed the trial, and one subject in the 3–8 year, 7.5 µg group was withdrawn at the Day 21 visit, before the second vaccination, for non-compliance with the protocol. All available data were included in the analyses, i.e., data on 240 subjects for the first vaccination, and 239 for the second vaccination. Table 1 shows the age and gender distribution within each age group; 47.9% of the overall population was male.

**Table 1:** Age and sex distribution across groups.

	Age and vaccine formulation group: dose and adjuvant content							
	9 to 17 years		3 to 8 years		6 to 35 months			
	30 µg+Al	7.5 µg	30 µg+Al	7.5 µg	30 µg+Al	15 µg+Al	7.5 µg	3.8 µg
**N randomized at day 0**	30	30	30	30	30	30	30	30
**Age in years**								
Mean (standard deviation)	12.7 (2.6)	12.3 (2.2)	6.5 (1.9)	5.8 (1.8)	1.6 (0.6)	2.0 (0.8)	1.7 (0.7)	1.8 (0.8)
[Min; Max]	9.0; 17.3	9.1; 16.8	3.0; 8.7	3.0; 8.9	0.5; 3.0	0.5; 3.0	0.6; 3.0	0.5; 2.9
**Gender n (%)**								
Male	14 (47)	18 (60)	14 (47)	16 (53)	14 (47)	14 (47)	12 (40)	13 (43)
Female	16 (53)	12 (40)	16 (53)	14 (47)	16 (53)	16 (53)	18 (60)	17 (57)

### Safety

All tested formulations of the A/H5N1 vaccine appeared well tolerated in all age groups over the 42 day period of observation. There were no vaccine related serious adverse events, no other significant adverse events and only two subjects experienced an unsolicited adverse event judged to be vaccine related: one subject had a maculo-papular rash which spontaneously disappeared after one day, and one subject had an injection site papule and mild itching which spontaneously resolved after 2 days. Both subjects were 2 year-olds and had received the 15 µg+Al formulation. These adverse events were not immediate, they were of short duration and no action was taken. Only five solicited systemic reactions were classed as severity grade 3 (one case each of headache, irritability, lost appetite, vomiting, and fever, all of which occurred with the non-adjuvanted vaccine in children younger than 3 years).

Combining data from children of all three age groups, the proportion experiencing at least one solicited injection site reaction in the seven days following the first injection of the 30 µg+Al vaccine was 57% (N = 90, 95% confidence interval: 46–67), and was 44% (95% CI: 34–55) following the first injection of 7.5 µg. The proportion experiencing solicited systemic reactions after the first injection was the same with each of these two formulations: 44% (95% CI: 34–55). Although sample sizes per group were too small to draw conclusions, no differences in vaccine reactogenicity were apparent between any of the age or vaccine formulation groups (Tables 2 and 3). Reactogenicity was no higher after the second injection, and indeed appeared to be lower than after the first: the overall incidences of solicited injection site reactions in the seven days after the second injection of 30 µg+Al and 7.5 µg were 47% (95% CI: 36–58) and 40% (95% CI: 30–51), and the corresponding incidences of solicited systemic reactions were 39% (95% CI: 29–50) and 34% (95% CI: 24–45). This trend for fewer reactions after the second injection was observed in all age and vaccine formulation subgroups (data not shown).

**Table 2:** Reactogenicity within 7 days after first vaccination in children aged 3–17 years: number and proportion subjects per age and vaccine group experiencing each solicited reaction at least once during period Day 0–7.

	9 to 17 years		3 to 8 years	
	30 µg+Al (N = 30)	7.5 µg (N = 30)	30 µg+Al (N = 30)	7.5 µg (N = 30)
	n (%)	n (%)	n (%)	n (%)
**Injection site reactions**				
Pain	12 (40%)	11 (37%)	19 (63%)	11 (37%)
Ecchymosis*	0	0	1 (3%)	0
Erythema*	7 (23%)	6 (20%)	1 (3%)	2 (7%)
Swelling*	1 (3%)	0	1 (3%)	1 (3%)
Induration*	0	1 (3%)	3 (10%)	1 (3%)
**Systemic reactions**				
Fever†	2 (7%)	2 (7%)	3 (10%)	5 (17%)
Headache	3 (10%)	6 (20%)	5 (17%)	5 (17%)
Malaise	1 (3%)	2 (7%)	3 (10%)	8 (27%)
Myalgia	3 (10%)	5 (17%)	12 (40%)	4 (13%)
Shivering	0	2 (7%)	0	1 (3%)

* any measurable reaction >0 cm.

† oral temperature ≥37.4°C.

N is the number of participants for whom each reaction was solicited, and for whom data are available.

**Table 3:** Reactogenicity within 7 days after first vaccination in children aged 6–35 months: number and proportion subjects per age and vaccine group experiencing each solicited reaction at least once during period Day 0–7.

	6 to 35 months			
	30 µg+Al	7.5 µg	15 µg+Al	3.8 µg
	n/N (%)	n/N (%)	n/N (%)	n/N (%)
**Injection site reactions** *				
Tenderness (6–23 months only)	10/23 (44%)	8/24 (33%)	4/14 (29%)	3/15 (20%)
Pain (24–35 months only)	2/7 (29%)	1/6 (17%)	7/16 (44%)	9/15 (60%)
Ecchymosis (24–35 months only)†	0/7	2/6 (33%)	1/16 (6%)	2/15 (13%)
Erythema (6–35 months)†	5/30 (17%)	6/30 (20%)	8/30 (27%)	5/30 (17%)
Swelling (6–35 months)†	2/30 (7%)	3/30 (10%)	3/30 (10%)	2/30 (7%)
Induration (6–35 months)†	0/30	3/30 (10%)	4/30 (13%)	3/30 (10%)
**Systemic reactions** *				
Fever (6–35 months)‡	4/30 (13%)	5/30 (17%)	7/30 (23%)	4/30 (13%)
Headache (24–35 months only)	1/7 (14%)	0/6	2/16 (13%)	4/15 (27%)
Malaise (24–35 months only)	2/7 (29%)	2/6 (33%)	3/16 (19%)	5/15 (33%)
Myalgia (24–35 months only)	1/7 (14%)	0/6	2/16 (13%)	5/15 (33%)
Shivering (24–35 months only)	0/7	0/6	0/16	2/15 (13%)
Vomiting (6–23 months only)	7/23 (30%)	7/24 (29%)	1/14 (7%)	0/15
Abnormal crying (6–23 months only)	4/23 (17%)	8/24 (33%)	7/14 (50%)	1/15 (7%)
Drowsiness (6–23 months only)	4/23 (17%)	4/24 (17%)	2/14 (14%)	0/15
Loss of appetite (6–23 months only)	5/23 (22%)	8/24 (33%)	4/14 (29%)	2/15 (13%)
Irritability (6–23 months only)	7/23 (30%)	10/24 (42%)	5/14 (36%)	2/15 (13%)

* Injection site tenderness, vomiting, abnormal crying, drowsiness and loss of appetite were solicited only for children younger than 24 months; injection site pain, or ecchymosis, headache, malaise, myalgia and shivering were solicited only for children older than 24 months. Other injection site reactions were solicited for all children.

† any measurable reaction >0 cm.

‡ oral temperature ≥37.4°C.

N is the number of participants for whom each reaction was solicited, and for whom data are available.

Fever, the only consistently evaluated and objectively measurable solicited systemic reaction, affected between 2 and 7 children per group of 30 after the first injection and between 0 and 8 after the second, and tended to occur more frequently among younger children.

### Haemagglutination inhibition antibody response

**Figure 1:**
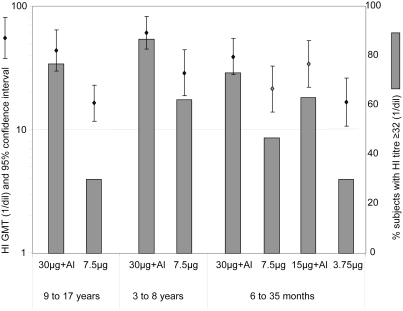
Haemagglutination inhibition antibody response 21 days after two injections, 21 days apart of adjuvanted or non-adjuvanted H5N1 vaccine. Results are presented per age and vaccine formulation group as geometric mean titres (GMT) and the proportion of subjects with titres ≥32.

Before vaccination, none of the subjects had detectable HI antibodies to H5N1. The first vaccination induced an HI response in at least one subject in each group of 30 children, but titres remained low: the GMT 21 days after the first vaccination ranged from 4.29 (95% CI: 3.8–4.83) to 8.48 (95% CI: 5.34–13.5) per group. After the second vaccination, titres increased in all groups: GMTs ranged from 16.4 (95% CI: 11.7; 22.8) and 60.4 (95% CI: 44.6; 81.9), and the seroresponse rate (number of subjects with titres ≥32) ranged between 30% and 77% (Figure 1). Combining all age groups, GMTs were higher after two injections of 30 µg+Al than after two injections of 7.5 µg: 46.9 (95% CI: 38.6; 56.8), compared with 21.5 (95% CI: 17.1;27.0). The corresponding GMTRs between D0 and D42 were 11.7 and 5.44. Seroresponse rates were also higher: 79% (95% CI: 69.0; 86.8) compared with 46% (95% CI: 35.4; 57.0). These higher responses with the 30 µg+Al formulation were observed consistently in all age groups (Figure 1). In the youngest age group, half dose formulations appeared to be slightly less immunogenic than their full dose equivalents, but 95% confidence intervals were largely overlapping. Finally, there was no effect of age on the HI immune response.

### Neutralising antibody responses

Neutralising Ab responses followed a similar pattern to those of HI. The highest proportion (≥90%) of individuals in each age group displaying a four-fold or greater rise in titre between day 0 and 42 was observed after two injections of the 30 µg+Al vaccine, as were the highest GMTs (Table 4).

**Table 4:** Neutralising antibody response 21 days after one and two injections, 21 days apart of adjuvanted or non-adjuvanted H5N1 vaccine.

	Age and vaccine formulation group: dose and adjuvant content							
	9 to 17 years		3 to 8 years		6 to 35 months			
	30 µg+Al	7.5 µg	30 µg+Al	7.5 µg	30 µg+Al	15 µg+Al	7.5 µg	3.8 µg
**After 1st vaccination (Day 21)**								
GMT (95%CI)	11.8 (6.95; 20.2)	7.26 (5.34; 9.87)	7.40 (5.09; 10.7)	7.69 (5.36; 11.0)	5.28 (4.72; 5.91)	5.82 (4.58; 7.38)	6.19 (4.48; 8.57)	6.75 (5.04; 9.04)
**After 2^nd^ vaccination (Day 42)**								
GMT (95%CI)	92.1 (61.6; 138)	33.5 (22.9; 49.0)	106 (84.6; 133)	54.2 (35.3; 83.1)	72.1 (49.1; 106)	60.2 (36.2; 100)	40.7 (25.0; 66.3)	30.4 (18.6; 49.5)
n/N (%) 2-fold increase day 0–42	29/30 (97)	27/30 (90)	30/30 (100)	29/29 (97)	29/30 (97)	28/30 (93)	25/30 (83)	24/30 (80)
n/N (%) 4-fold increase day 0–42	29/30 (97)	20/30 (67)	30/30 (100)	21/29 (72)	27/30 (90)	25/30 (83)	21/30 (70)	18 (60)

N is the number of participants in each group for whom data are available at each timepoint necessary to calculate each table entry.

### Cellular immune responses

**Figure 2:**
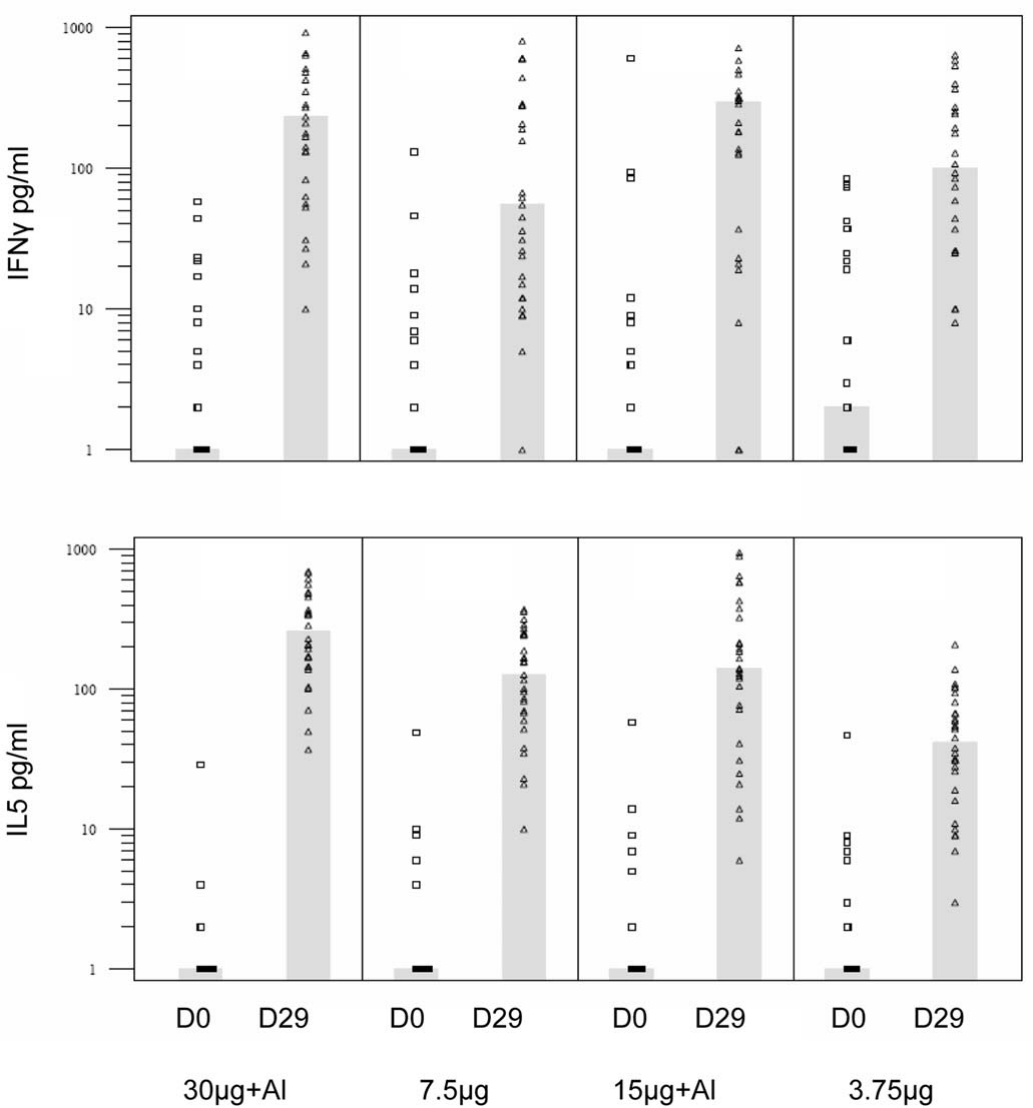
Levels of IFNγ and IL5 secreted after in vitro re-stimulation with recombinant H5 haemagglutinin by cells obtained before and eight days after two injections, 21 days apart, of adjuvanted or non-adjuvanted H5N1 vaccine in groups of children aged 6–35 months. Symbols represent results from individual samples, bars indicate the median level.

Before vaccination of these 6–35 month olds, an H1N1-specific response (data not shown) and a weak cross-reactive CD4 response against H5 (Figure 2) were detected, with both Th1 (IFNγ) and Th2 (IL13) cytokine secretion. This response was mainly directed against epitopes included in the H1N1 vaccine strain. More than 50% subjects were negative for H1N1 epitopes and only a low reactivity was found against the recombinant HA antigen in 8/112 subjects for IFN-γ and 26/114 subjects for IL13.

Eight days after the second vaccination, concentrations of both Th1 (IFNγ) and Th2 (IL-5 and IL-13) cytokines increased in all study groups, with a predominant secretion of IL13, even in absence of aluminium adjuvant (figure 2). Vaccination did not significantly increase TNFα secretion and induced a predominantly Th2 response against rHA, as reflected by the weak IFNγ/IL13 ratios of between 0.077 (95%CI: 0.041–0.145) in the 7.5 µg group and 0.211 (0.118–0.376) in the 3.75 µg grroup.

## Discussion

Children and young adults are likely to be particularly vulnerable to infection during an influenza pandemic and an important source of infection for others. Some 40% of cases have been predicted to occur in individuals aged 19 years or younger [16]. Among human cases of highly pathogenic avian influenza A/H5N1 virus infection confirmed over the last decade, a disproportionately high number have been children, although this is possibly due, in part, to the proximity between children and poultry in areas of Asia where most of these cases have occurred [17]. A recent study profiled the social contact networks of school age children and teenagers in the USA as a way of assessing the potential for influenza transmission within this population [8]. The authors suggested that high school students are likely to form the local transmission backbone for the next influenza pandemic.

Vaccination of children will therefore form an essential element of pandemic influenza vaccination programs. Our study was designed to evaluate the safety and immunogenicity of different formulations of an A/H5N1 vaccine in children. The two full-dose formulations chosen for this study and tested in all age groups of children (the adjuvanted 30 µg formulation and the non-adjuvanted 7.5 µg formulation), had previously been evaluated in a trial in French adults, and found to be well tolerated, immunogenic and able to induce cross-reactive antibodies[13]. The two half-dose formulations evaluated in the youngest group of children (aged 6–35 months) were chosen in line with recommendations for seasonal influenza vaccination of young children [18], for whom a half of the standard adult vaccine dose can be used. All formulations of the H5N1 vaccine appeared to be well tolerated with notably no evidence of increased reactogenicity after the second vaccination, no serious or significant adverse events and very few severity grade 3 solicited reactions. There were no marked differences in reactogenicity between the higher-dose adjuvanted groups and the lower dose non-adjuvanted groups. In accordance with published data with licensed seasonal influenza, the youngest group of children tended to have a higher incidence of fever [19].

It has been argued that due to the existence of numerous undetected mild or asymptomatic cases [20], the true human case fatality rate influenza A (H5N1) is considerably lower than the ∼60% calculated based only on confirmed cases reported by the WHO [2]. Indeed around 10% of a cohort of Hong Kong poultry workers had anti-H5 antibodies after the 1997 outbreak of H5N1 [21]. The children enrolled to our study in Bangkok showed no evidence of having been previously exposed to H5N1 influenza. They had no detectable antibody responses before vaccination, and the low levels of immune response seen after the first vaccination are concordant with a primary immune response, rather than a booster response. The H5N1-specific CD4 responses seen in some 6–35 month-old children is likely to be due to cross reactive T-cell responses stimulated by prior infection by other influenza strains. Such cross-reactive cellular responses between influenza strains have been described previously [22]–[24].

Antibody responses increased in all groups after the second vaccination. In terms of both geometric mean titres and the HI seroresponse rate, responses were highest among children vaccinated with the adjuvanted 30 µg formulation. Antibody responses to both the adjuvanted 30 µg formulation and the non-adjuvanted 7.5 µg formulation appeared to be at least as good as, if not better than those observed with the same vaccine formulations in a previous study in adults [13]. It should be pointed out that vaccines in this study were presented in ready-to-use multi-dose vials, whereas in the adult study, vaccines were presented as single dose vials of vaccine and adjuvant for extemporaneous preparation. Both assays used to document the antibody response are functional assays, nevertheless, in absence of an established correlate of protection, it is unclear how the haemagglutination inhibiting and neutralising antibody responses documented in this study would translate to efficacy against infection or disease in a pandemic context. In their guidance for the licensing of pandemic vaccines, the EMEA acknowledges this uncertainty and requires that mock up vaccines be at least able to meet the three criteria defined for the vaccination of adults or elderly adults against seasonal influenza; i.e., a GMTR of at least 2.5 or 2.0, a seroprotection rate of at least 70 or 60% and a seroconversion or significant titre increase rate of at least 40 or 30% [14], [15]. Although in our study, the seroresponse threshold considered was 1∶32 instead of the 1∶40 in the EMEA criteria, with a GMTR of 11.7 and a seroresponse and seroconversion rate of 79%, the 30 µg+Al formulation in children in this study satisfy all three criteria.

Immune responses to the non-adjuvanted vaccines in Thai children in our study appeared comparable to or higher than those observed in a study among US children, despite a 6–12-fold difference in the amount of antigen: after two doses of 45 µg HA without adjuvant, the 38% had titres >1∶40 [25]. Several factors potentially contribute to this difference, including genetic factors, and the lack of standardization of assay methods between laboratories.

We explored the Th1 and Th2 cytokine secretion profile in subjects before and after vaccination. These analyses were performed in the youngest group of children (6–35 months) as it is in immunologically immature infants that immune responses are most biased towards a Th2 response [26]–[28]. As expected, we observed low levels of cytokine secretion with a Th2-dominant profile before vaccination. In all groups, vaccination induced a Th2-biased response to the H5 protein. Although the study design did not allow the adjuvant effect to be separated from any antigen dose effect, there was no evidence of increased Th2-dominance in among subjects vaccinated with an adjuvanted vaccine.

In summary, these influenza A/H5N1 vaccines appeared immunogenic and well tolerated in a population of naïve children and infants from the age of 6 months, with the 30 µg+Al vaccine formulation eliciting the greatest immune responses, at least as good as those previously seen with the same vaccine formulation in adults. Given the likely burden of disease in children and their role in disease transmission, the vaccination of children will form an essential part of pandemic vaccination campaigns to control the spread of the disease. Furthermore, as the logistics of vaccine administration during a pandemic are expected to be particularly challenging, the ability to use the same vaccine across age groups will be a major advantage.

## Supporting Information

Protocol S1 Trial Protocol
(1.12 MB PDF)

Checklist S1 CONSORT checklist
(0.06 MB DOC)
