# Supplementary material for: Safety, Humoral and Cell Mediated Immune Responses to Two Formulations of an Inactivated, Split-Virion Influenza A/H5N1 Vaccine in Children
Source: PLoS One. 2008 Dec 29;3(12):e4028. doi: 10.1371/journal.pone.0004028 (PMC2605261; doi:10.1371/journal.pone.0004028)
Supplement: Protocol S1 — Trial Protocol (1.12 MB PDF) [file pone.0004028.s001.pdf]

sanofi pasteur
The vaccines business of sanofi-aventis Group

Safety and Immunogenicity of Two Different Formulations of an Intramuscular A/H5N1 Inactivated, Split Virion Pandemic Influenza Vaccine in Children

A Phase II, open, randomized, multicenter trial including 240 subjects in three groups: 60 subjects aged 9 to 17 years, 60 subjects aged 3 to 8 years, and 120 subjects aged 6 to 35 months to be enrolled in a step-down design (based on age) following review of Day 0 to 7 safety data from the previous age group. Each child will receive two administrations 21 days apart and a booster vaccination at either 6 or 12 months after the first vaccination.

Clinical Trial Protocol Amendment 1

Trial Code: **GPA04** **Development Phase:** II

**Sponsor:**	Sanofi Pasteur SA<br>2, avenue Pont Pasteur, 69367 Lyon cedex 07, France
**Investigational Product:**	A/H5N1 inactivated, split-virion influenza virus
**Form/Route:**	Liquid/Intramuscular
**Manufacturer:**	Sanofi Pasteur SA<br>1541 avenue Marcel Mérieux<br>69280 Marcy l'Etoile, France
**Principal/Coordinating Investigators:**	Tawee CHOTPITAYASUNONDH, M.D (Coordinating Investigator)<br>Queen Sirikit National Institute of Child Health (Children's Hospital)<br>Bangkok, Thailand<br>Tel: +66 2 354 8400/Fax: +66 2 354 8400<br>Usa THISYAKORN, M.D<br>Chulalongkorn Hospital<br>Bangkok, Thailand<br>Tel: +66 2 256 4930/Fax: +66 2 256 4930
**Sponsor's Responsible Medical Officer (Sponsor's signatory):**	Melanie SAVILLE MB BS – Clinical Team Leader<br>Clinical Development, sanofi pasteur,<br>Tel.:(+33) 4 37 37 59 22/Fax (+33) 4 37 37 74 38
**Clinical Scientist:**	Stephanie PEPIN PhD<br>Clinical Development, sanofi pasteur<br>Tel:(+33) 4 37 37 58 50/Fax (+33) 4 37 37 74 38
**Clinical Research Associate (CRA):**	Fabienne ROCHE<br>Clinical Operations, sanofi pasteur<br>Tel: (+33) 4 37 37 99 90/Fax (+33) 4 37 37 79 28
**Trial Team:**	See Appendix 1
**Version and Date of the Protocol:**	Version 5.0, dated 21 May 2007

This protocol (Version 5.0, 21 May 2007) is the first amendment to the initial protocol Version 3.0 (17 January 2007). Versions 1.0, 2.0 and 4.0 were not used.

Synopsis

**Company:**	sanofi pasteur
**Investigational Product/ Drug Product:**	A/H5N1 inactivated split virion pandemic influenza vaccine made in embryonated eggs
**Active Substance:**	Inactivated influenza virus split with octoxinol 9

**Title of the trial:**	Safety and Immunogenicity of Two Different Formulations of an Intramuscular A/H5N1 Inactivated, Split Virion Pandemic Influenza Vaccine in Children
**Development phase:**	II
**Principal/Coordinating Investigator:**	Tawee CHOTPITAYASUNONDH, M.D (Coordinating Investigator)<br>Usa THISYAKORN, M.D
**Investigators and trial centers:**	Tawee CHOTPITAYASUNONDH, M.D (Coordinating Investigator)<br>Queen Sirikit National Institute of Child Health (Children's Hospital)<br>Bangkok, Thailand<br>Tel: +66 2 354 8400/Fax: +66 2 354 8400<br><br>Usa THISYAKORN, M.D<br>Chulalongkorn Hospital<br>Bangkok, Thailand<br>Tel: +66 2 256 4930/Fax: +66 2 256 4930
**Planned trial period:**	June 2007 (FVFS) to September 2008 (LVLS if 6 month booster and 6 month safety follow-up after booster) March 2009 (LVLS if 12 month booster and 6 month safety follow-up after booster).
**Objectives:**	• To describe the safety profiles (injection site reactions and systemic events) during the 21 days following each injection (including the booster injection).<br>• To describe the immune response 21 days after the first and the second injection of each formulation.<br>• To describe the antibody persistence until the time of the booster vaccination at 6 or 12 months* after the first vaccination.<br>• To describe the immune response 21 days after a booster vaccination administered at 6 or 12 months after the first vaccination.<br>* The timing and dosage of the booster vaccination is dependent on the results of ongoing studies (GPA01 and GPA02). The possibilities of a booster at 6 or 12 months (and two flow-charts) are included as options in this protocol.
**Endpoints:**	**Safety**<br>• Occurrence, nature, (Medical Dictionary for Regulatory Activities [MedDRA]), duration, severity, and relationship to vaccination for any unsolicited systemic adverse events reported in the 30 minutes after each injection.<br>• The occurrence, time to onset, number of days of occurrence, severity, and seriousness of solicited (prelisted in the subject diary and Case Report Form [CRF]) injection site reactions and systemic reactions occurring within 7 days following each injection will be reported (including the booster injection).<br>• The occurrence, nature (MedDRA preferred term), severity, relationship to vaccination and seriousness of unsolicited (spontaneously reported) adverse events within 21 days following each injection will be reported (including the

**Company:**	sanofi pasteur
**Investigational Product/ Drug Product:**	A/H5N1 inactivated split virion pandemic influenza vaccine made in embryonated eggs
**Active Substance:**	Inactivated influenza virus split with octoxinol 9

booster injection).

- The occurrence, nature, time to onset, and relationship to vaccination of serious adverse events (SAEs) during the whole study period will be reported.
- The occurrence of the following reactions (MedDRA Preferred Terms given in parentheses) in the three days following each injection will be more especially reported (as defined by the Committee for Medicinal Prodcuts for Human Use [CHMP] Note for Guidance [CPMP/BWP/214/96]) (including the booster injection).
  - Injection site induration >5 cm observed for more than 3 days.
  - Injection site ecchymosis (injection site hemorrhage).
  - Rectal equivalent temperature >38°C for 24 hours or more (pyrexia).
  - Malaise.
  - Shivering (chills).

Immunogenicity

Anti-Hemagglutinin (anti-HA) Ab titers against the A/H5N1 strain will be expressed as described below (the assay will be performed using horse erythrocytes for all subjects and using turkey erythrocytes for subjects in Group 1 and Group 2; anti-HA titers will not be measured using turkey erythrocytes for Group 3 due to the extra blood sample volume that would be required):

- Hemagglutination Inhibition (HI) titer obtained in duplicate on D0, D21, and D42, and summarized at the subject level by individual geometric means (GMs) of duplicates at each timepoint. The following endpoints will be derived:
  - Individual titer ratios D21/D0, D42/D0, and D42/D21.
  - Proportion of subjects with a titer ≥40 (turkey) or ≥32 (horse) 1/dilution [dil]) on D0, D21, and D42.
  - Seroconversion (for subjects with a titer <10 [turkey] or <8 [horse] [1/dil] on D0: post-injection titer ≥40 [turkey] or ≥32 [horse] [1/dil])

  or

  - Significant increase (for subjects with a titer ≥10 [turkey] or ≥8 [horse] [1/dil]: ≥four-fold increase of the titer) on D21 and D42.
- Seroneutralization (SN) titer obtained in duplicate on D0, D21, and D42, and summarized at the subject level by individual GM of duplicates at each timepoint. The following endpoints will be derived:
  - Individual titer ratios D21/D0, D42/D0, and D42/D21.
  - Two- and four-fold increase from D0 to D21, from D0 to D42, and from D21 to D42.

Antibody persistence

Anti-HA titer (HI method) and neutralizing Ab titer (SN method) in duplicate at M3 and prior to the booster vaccination at M6 (D180), or at M3 and M6 and prior to the booster vaccination at M12 (D365).

**Company:**	sanofi pasteur
**Investigational Product/ Drug Product:**	A/H5N1 inactivated split virion pandemic influenza vaccine made in embryonated eggs
**Active Substance:**	Inactivated influenza virus split with octoxinol 9

	**Booster vaccination response**<br>• HI titer obtained in duplicate at V07 (M6) (for 6-month booster design) or V08 (M12) (for 12-month booster design), V08 (M6+21 days) (for 6-month booster design) or V09 (M12+21 days) (for 12-month booster design), and summarized at the subject level by individual GMs of duplicates at each timepoint. The following endpoints will be derived:<br>• Individual titer ratios V08/V07 (for 6-month booster design) or V09/V08 (for 12-month booster design).<br>• Proportion of subjects with a titer ≥40 (turkey) or ≥32)horse) (1/dil) at V07 and V08 (for 6-month booster design) or V08 and V09 (for 6-month booster design).<br>• Seroconversion (for subjects with a titer <10 [1/dil] at V07 (for 6-month booster design) or V08 (for 12-month booster design): post-injection titer ≥40 [turkey] or ≥32 [horse] [1/dil]) at V08 (for 6-month booster design) or V09 (for 12-month booster design),<br>or<br>• Significant increase (for subjects with a titer ≥10 [1/dil]: ≥four-fold increase of the titer) at V08 (for 6-month booster design) or V09 (for 12-month booster design).<br>• SN titer obtained in duplicate at V07 and V08 (for 6-month booster design) or V08 and V09 (for 12-month booster design) and summarized at the subject level by individual GMs of the duplicates at each timepoint. The following endpoints will be derived:<br>• Individual titer ratios V08/V07 (for 6-month booster design) or V09/V08 (for 12-month booster design).<br>• Two- and four-fold increase from V07 to V08 (for 6-month booster design) or V08 to V09 (for 12-month booster design).
**Statistical methods for the objective:**	The analyses will be mainly descriptive.<br>• The safety analysis will describe the incidence of solicited reactions within 7 days after each injection and of unsolicited adverse events (AEs) within 21 days after each and any injection. The 95% confidence intervals (CI) of point estimates of pre-defined solicited reactions will be calculated using the exact binomial distribution (Clopper-Pearson method) for proportions. Additionally, for each injection, the safety data will be presented according to the EMEA safety criteria.<br>• For the immunological parameters, 95% CIs of GM of titers at each timepoint and GM of titers ratios between timepoints will be calculated using normal approximation of the Log_{10}-transformed titers and then back-transformation (primo and booster vaccinations). Additionnaly, the proportion of subjects with an anti-HA antibody titer ≥40 (turkey) or ≥32 (horse) (1/dil) and the seroconversion (or significant increase) rate will be presented for HI titers and two- and four-fold increases will be presented for SN. 95% confidence intervals (CIs) for these rates will be calculated using the exact binomial distribution (Clopper-Pearson method) for proportions. HI and SN antibody titers will also be presented by frequency.

**Company:**	sanofi pasteur
**Investigational Product/ Drug Product:**	A/H5N1 inactivated split virion pandemic influenza vaccine made in embryonated eggs
**Active Substance:**	Inactivated influenza virus split with octoxinol 9

**Observational objectives:**	• To describe in Group 3 the cellular mediated immune (CMI) response before the first vaccination and 8 days after the second vaccination.
**Observational Endpoints:**	**Cellular mediated immune (CMI) response**<br>Secretion of a panel of Th1 (interferon-gamma [IFN-γ], tumor necrosis factor-alpha [TNF-α], interleukin-2 [IL-2]), and Th2 (IL-5, IL-4, IL-10, IL-13) cytokines by peripheral blood mononuclear cells (PBMCs), upon in vitro re-stimulation with vaccine antigens will be quantified before the first vaccination and 8 days after the second vaccination.
**Methodology/Trial Design:**	Phase II, multicenter, randomized, open trial. Subjects will be enrolled in a step-down design (based on age) following internal review of Day 0 to 7 safety data for each primary series vaccination from the previous age group.<br>Primary series vaccination at D0 and D21 and booster vaccination at either M6 or M12 (according to the Ab persistence data from GPA01 and GPA02).
**Planned Sample Size:**	<u>Group 1 (9 to 17 years) (N=60)</u><br>7.5 µg HA without adjuvant (N=30)<br>30 µg HA + aluminum hydroxide as adjuvant (600 µg aluminum) (N=30)<br><u>Group 2 (3 to 8 years) (N=60)</u><br>7.5 µg HA without adjuvant (N=30)<br>30 µg HA + aluminum hydroxide as adjuvant (600 µg aluminum) (N=30)<br><u>Group 3 (6 to 35 months) (N=120)</u><br>3.75 µg HA without adjuvant (N=30)<br>7.5 µg HA without adjuvant (N=30)<br>15 µg HA + aluminum hydroxide as adjuvant (300 µg aluminum) (N=30)<br>30 µg HA + aluminum hydroxide as adjuvant (600 µg aluminum) (N=30)
**Vaccination and Specimen Collection Schedules and Duration of Follow-up:**	All subjects will receive two vaccinations separated by 21 days plus a booster vaccination at M6 or M12, in an 8 or 9 visit schedule (and a safety follow-up visit). The expected total duration is 12 or 18 months (depending whether the booster is administered at 6 or 12 months), including a 6-month follow-up for safety after the booster vaccination.<br>A blood sample (7 mL for Groups 1 and 2, and 2 mL for Group 3) will be taken immediately prior to the first vaccination and at each subsequent visit for serology analyses. In addition, a 2 mL blood sample will be taken for Group 3 only prior to the first vaccination and 8 days after the second vaccination for analysis of the CMI response.
**Investigational Product:**<br><br>**Form:**<br>**Composition:**	A/H5N1 inactivated split-virion pandemic influenza vaccine made in embryonated eggs with or without aluminum hydroxide adjuvant.<br>Ready-to-use multidose 7 mL format vials containing 5 mL of vaccine.<br>Monovalent (drug substance) A/H5N1 inactivated influenza vaccine split with octoxinol-9 produced on embryonated hens' eggs.

**Company:**	sanofi pasteur
**Investigational Product/ Drug Product:**	A/H5N1 inactivated split virion pandemic influenza vaccine made in embryonated eggs
**Active Substance:**	Inactivated influenza virus split with octoxinol 9

**Route:**<br><br>**Batch Number:**	Two formulations will be used:<br>- 30 µg HA with aluminum hydroxide as adjuvant (600 µg aluminum)/0.5 mL.<br>- 7.5 µg HA without adjuvant/0.6 mL.<br>*Note: vaccines contain 45 µg Thimerosal per full dose (25 µg Thimerosal per half dose, i.e. doses of 3.75 µg HA without adjuvant and 15 µg HA + aluminum hydroxide as adjuvant [300 µg]).*<br>For each formulation, intramuscular injection into the deltoid (subjects ≥1 year of age) and thigh (subjects <1 year of age).<br>The volumes injected for each group are detailed below:<br>Group 1: 0.5 mL of 30µg+Ad/0.5mL (Dose 30 µg HA + Ad)<br>0.6 mL of 7.5µg/0.6mL (Dose 7.5 µg HA)<br>Group 2: 0.5 mL of 30µg+Ad/0.5mL (Dose 30 µg HA + Ad)<br>0.6 mL of 7.5µg/0.6mL (Dose 7.5 µg HA)<br>Group 3: 0.25 mL of 30µg+Ad/0.5mL (Dose 15 µg HA + Ad)<br>0.3 mL of 7.5µg/0.6mL (Dose 3.75 µg HA)<br>0.5 mL of 30µg+Ad/0.5mL (Dose 30 µg HA + Ad)<br>0.6 mL of 7.5µg/0.6mL (Dose 7.5 µg HA)<br>Note: Ad = aluminum hydroxide adjuvant<br>To be determined
**Inclusion criteria:**	***All subjects:***<br>1) Able to attend all scheduled visits and to comply with all trial procedures.<br>***Children/Adolescents aged ≥2 years to <18 years:***<br>2) Aged ≥2 years to <18 years on the day of inclusion.<br>3) Informed Consent Form signed by one parent or another legal representative and by an independent witness if the parent/legal representative is illiterate. In addition, two assent forms signed: one signed by subjects aged between 7 and 12 years and the other signed by subjects aged between 13 and 17 years.<br>4) For a female, inability to bear a child or negative urine pregnancy test (as applicable).<br>5) For a female of child-bearing potential, use of an effective method of contraception or abstinence for at least 4 weeks prior to the first vaccination and at least 4 weeks after the last vaccination (as applicable).<br>***Infants/toddlers aged ≥6 months to <2 years:***<br>6) Aged ≥6 months to <2 years on the day of inclusion.<br>7) Born at full term of pregnancy (≥37 weeks) with a birth weight ≥2 kg.<br>8) Informed Consent Form signed by one parent/legally acceptable representative and an independent witness if the parent/legally acceptable representative is

**Company:**	sanofi pasteur
**Investigational Product/ Drug Product:**	A/H5N1 inactivated split virion pandemic influenza vaccine made in embryonated eggs
**Active Substance:**	Inactivated influenza virus split with octoxinol 9

	illiterate.<br>9) Subject who completed vaccination according to the national immunization schedule.
**Exclusion criteria:**	***All subjects:***<br>1) Participation in another clinical trial in the 4 weeks preceding the first trial vaccination.<br>2) Planned participation in another clinical trial during the present trial period.<br>3) Congenital or acquired immunodeficiency, immunosuppressive therapy such as anti-cancer chemotherapy or radiation therapy within the preceding 6 months, or long-term systemic corticosteroid therapy.<br>4) Systemic hypersensitivity to any of the vaccine components or history of life-threatening reaction to the trial vaccine or a vaccine containing the same substances (egg proteins, chick proteins, thimerosal, aluminum, neomycin, formaldehyde, and octoxinol 9).<br>5) Chronic illness at a stage that could interfere with trial conduct or completion.<br>6) Blood or blood-derived products received in the past 3 months.<br>7) Any vaccination in the 4 weeks preceding the first trial vaccination.<br>8) Vaccination planned in the 4 weeks following any trial vaccination.<br>9) History of the H5N1 infection (confirmed either clinically, serologically or virologically).<br>10) Known HIV, Hepatitis B (HBsAg) or Hepatitis C seropositivity.<br>11) Previous vaccination with an avian flu vaccine.<br>12) Subject at high risk of the H5N1 infection during the trial.<br>13) Thrombocytopenia or bleeding disorder contraindicating IM vaccination.<br>14) Febrile illness (rectal equivalent temperature ≥38.0°C) on the day of inclusion.<br>***Children/Adolescents aged ≥2 years to <18 years:***<br>15) Breast-feeding mothers.<br>16) Current alcohol abuse or drug addiction that may interfere with the subject's ability to comply with trial procedures (if applicable, depending on the subject's age).<br>***Infants/toddlers aged ≥6 months to <2 years:***<br>17) History of seizures.
**Statistical methods:**	A first statistical analysis will be performed once all the data obtained 21 days after the second vaccination have been locked (first partial database lock), to address the safety and immunogenicity objectives (excluding CMI) of the D0 to D42 period.<br>A final statistical analysis will be performed when all the data obtained 21 days after the booster vaccination have been locked (second partial database lock), to address the

**Company:**	sanofi pasteur
**Investigational Product/ Drug Product:**	A/H5N1 inactivated split virion pandemic influenza vaccine made in embryonated eggs
**Active Substance:**	Inactivated influenza virus split with octoxinol 9

	immunogenicity and safety objectives for the booster vaccination and the antibody persistence objective.<br>An addendum will be produced after the final 6 month safety follow-up (final database lock).<br>All the main analyses will be descriptive. For the main parameters, 95% CIs of point estimates will be calculated using normal approximation for quantitative data and exact binomial distribution (Clopper-Pearson method) for proportions.<br>Additionally for Group 1 and Group 2, and after each injection, the safety data will be presented according to the safety criteria quoted in the Note for Guidance on harmonization requirements for Influenza vaccines (CHMP/ who received the first injection).<br>**Sample size**<br>The overall study cohort (N=240) will provide a probability >90% of detecting any AE with an incidence of 1% in the study, and a probability of 70% for each pooled adjuvanted and non adjuvanted group (N=120).

Figure 1: Phase II Trial, 9 Visits, 2 Vaccinations with a 6-month Booster Vaccination, 6 or 7 Blood Samplings, 12 Months Duration/Subject

Visit	V01	V02	V03	V04	V05	V06	V07	V08	6-month follow-up*
Visit interval	VAC1	VAC1 +8D	VAC2 = VAC1+21 D	VAC2+8 D	VAC2+21 D	VAC1+90 D	VAC3 = VAC1 +180D	VAC3 +21D	VAC1+ 365D
Indicative Days (D)	D0	D8+1D	D21±3D	D29+1D	VAC2+ 21D±3D	D90±8D	D180 ±8D	VAC3+ 21D±3D	D365± 15D
Indicative Months (M)						M3	M6		M12
Informed consent signed†	√								
Demography	√								
Body stature	√								
Axillary temperature	√		√				√		
Urine pregnancy test‡	√		√				√		
Physical examination	√	√	√	√	√	√	√	√	√§
Influenza-like illness and flu vaccination history	√						√		
Past and current significant medical history	√								
Inclusion & exclusion criteria	√								
Randomization	√						√ (if applic-able)		
Temporary and definite contraindications			√				√		
Blood sampling for serology (7 mL for Groups 1 and 2, 2 mL for Group 3)	√		√		√	√	√	√	
Blood sampling for CMI response (2 mL) (Group 3 only)	√			√					
Vaccine injection	√		√				√		
30-minute observation period	√		√				√		
Diary Card (DC)/ Memory Aid (MA):<br>Provided:<br>Checked:<br>Collected:	 <br>DC1 	 <br><br>DC1 	 <br>DC2<br><br>DC1	 <br><br>DC2 	 <br>MA1<br><br>DC2	 <br><br>MA1 	 <br>DC3<br><br>MA1	 <br>MA2<br><br>DC3	 <br><br><br>MA2**
Injection Site Reactions and Systemic Event Assessment	√	√	√	√	√		√	√	
Reportable Concomitant Medication	√	√	√	√	√		√	√	
Termination record					√			√	
Serious Adverse Events	Collected through the whole study period								

---

* A telephone call or a visit at the site is to be arranged 6 months after the booster vaccination to collect information on SAEs. In the event of an SAE, the Investigator may arrange a visit with the subject to obtain further information.

† In addition, one assent form to be signed by subjects aged 7 to 12 years and another assent form to be signed by subjects aged 13 to 17 years.

‡ For adolescents of child-bearing potential only.

§ Limited to medical interview if visit replaced by ‘phone call.

** MA2 to be returned by mail if visit replaced by ‘phone call.

Figure 2: Phase II Trial, 10 Visits, 2 Vaccinations with a 12-month Booster Vaccination, 7 or 8 Blood Samplings, 18 Months Duration/Subject

**Visit**	**V01**	**V02**	**V03**	**V04**	**V05**	**V06**	**V07**	**V08**	**V09**	**6-month follow-up***
**Visit interval**	**VAC1**	**VAC1 +8D**	**VAC2 = VAC1 +21D**	**VAC2 +8D**	**VAC2 +21D**	**VAC1 +90D**	**VAC1 +180D**	**VAC3 = VAC1 +365D**	**VAC3 +21D**	**VAC3 +180D**
**Indicative Days (D)**	**D0**	**D8 +1D**	**D21 ±3D**	**D29 +1D**	**VAC2+ 21D±3D**	**D90± 8D**	**D180± 8D**	**D365± 15D**	**VAC3+ 21D±3D**	**D545± 15D**
**Indicative Months (M)**						**M3**	**M6**	**M12**		**M18**
**Informed consent signed†**	√									
**Demography**	√									
**Body stature**	√									
**Urine pregnancy test‡**	√		√					√		
**Physical examination**	√	√	√	√	√	√	√	√	√	√§
**Influenza-like illness and flu vaccination history**	√							√		
**Past and current significant medical history**	√									
**Inclusion & exclusion criteria**	√									
**Randomization**	√							√ (if applic-able)		
**Temporary and definite contraindications**			√					√		
**Blood sampling for serology (7 mL for Groups 1 and 2, 2 mL for Group 3)**	√		√		√	√	√	√	√	
**Blood sampling for CMI response (2 mL) (Group 3 only)**	√			√						
**Axillary temperature**	√		√					√		
**Vaccine injection**	√		√					√		
**30-minute observation period**	√		√					√		
**Diary Card (DC)/ Memory Aid (MA):**<br>Provided:<br>Checked:<br>Collected:	DC1	DC1	DC2<br>DC1	DC2	MA1<br>DC2	MA1	MA2<br>MA1	DC3<br>MA2	MA3<br>DC3	MA3**
**Injection site reactions and Systemic Event Assessment**	√	√	√	√	√				√	
**Reportable Concomitant Medication**	√	√	√	√	√			√	√	
**Termination Record**					√		√		√	
**Serious Adverse Events**	Collected through the whole study period									

* A telephone call or a visit at the site is to be arranged 6 months after the booster vaccination to collect information on SAEs. In the event of an SAE, the Investigator may arrange a visit with the subject to obtain further information.

† In addition, one assent form to be signed by subjects aged 7 to 12 years and another assent form to be signed by subjects aged 13 to 17 years.

‡ For adolescents of child-bearing potential only.

§ Limited to medical interview if visit replaced by ‘phone call.

** MA3 to be returned by mail if visit replaced by ‘phone call.

Table of Contents

Synopsis **2**

List of Abbreviations **17**

1 Introduction **19**

1.1 Background 19

1.2 Subject Benefits / Potential Risks 21

1.3 Rationale 22

2 Trial Objectives **24**

2.1 Endpoints 25

2.2 Summary of Statistical Methodology for the Objectives 27

2.3 Observational Objective 27

3 Investigators and Trial Organization **27**

4 Independent Ethics Committee (IEC)/Institutional Review Board (IRB) **27**

5 Investigational Plan **28**

5.1 Description of the Overall Trial Design and Plan 28
5.1.1 Trial Design 28
5.1.2 Trial Plan 28
5.1.3 Visit Procedures 31
5.1.4 Planned Trial Calendar 38

5.2 Discussion of Trial Design 39

5.3 Selection of Trial Population 40
5.3.1 Recruitment Procedures 40
5.3.2 Participant Information and Consent 40
5.3.3 Inclusion Criteria 41
5.3.4 Exclusion Criteria 42
5.3.5 Temporary Contraindications 43
5.3.6 Removal of Participants from Treatment or Assessment 43
5.3.6.1 Conditions for Withdrawal 43
5.3.6.2 Lost to Follow-up Procedures 44
5.3.6.3 Termination Classification 44
5.3.6.4 Follow-up of Discontinuations 44
5.3.6.5 Follow-up and Reporting of Pregnancies 44
5.3.7 Medical History 45

5.4 Modification of the Trial and Protocol 45

5.5 Interruption of the Trial 45

6 Treatments 46

6.1 Vaccines Administered 46

6.2 Identity of Investigational Product(s) 46
6.2.1 Composition............ 46
6.2.2 Preparation and Administration............ 46
6.2.3 Precautions for Use............ 47
6.2.4 Dose Selection and Timing............ 47

6.3 Identity of Control Product 47

6.4 Identity of Other Product 47

6.5 Product Logistics 47
6.5.1 Labeling and Packaging............ 47
6.5.2 Storage and Shipment Conditions 48
6.5.3 Product Accountability............ 48
6.5.4 Replacement Doses............ 48
6.5.5 Return of Unused Products............ 49

6.6 Randomization/Allocation Procedures 49

6.7 Blinding and Code Breaking Procedures............ 49

6.8 Concomitant Therapy............ 49

6.9 Treatment Compliance............ 50

7 Specimens and Clinical Supplies............ 50

7.1 Management of Samples for Humoral Immune Response 51
7.1.1 Sample Collection for Serological Assessments 51
7.1.2 Sera Preparation of Blood Samples for Serological Assays............ 51
7.1.3 Sera Storage and Shipment of Samples for Serological Assays............ 51
7.1.4 Retention of Unused Serum Samples for Repository (Humoral)............ 52

7.2 Management of Samples for Cellular Mediated Immune Response 52
7.2.1 Sample Collection for Cellular Mediated Immune Response Assessments............ 52
7.2.2 Cellular Mediated Immune Response Assessment............ 52
7.2.3 Storage and Shipment of Samples for Cellular Mediated Immune Response Assessment 53

7.3 Clinical Supplies 53

8 Assessments Methods and Endpoints 53

8.1 Immunogenicity Endpoints and Assessments Methods............ 53
8.1.1 Endpoints............ 53
8.1.1.1 Observational Endpoints 55

8.1.2 Assessment Methods 55
8.1.2.1 Hemagglutination Inhibition Test 55
8.1.2.2 Seroneutralization 56
8.1.2.3 Cellular Mediated Immune Response 56
8.1.3 Handling of Missing Data and Outliers 56
8.2 Efficacy Endpoints and Assessments Methods 56
8.3 Safety Endpoints and Assessments Methods 56
8.3.1 Definitions 56
8.3.2 Endpoints 59
8.3.2.1 Exploratory Endpoints 59
8.3.3 Safety Assessment methods 59
8.3.3.1 30-Minute Observation Period 59
8.3.3.2 Reactogenicity (Solicited Reactions from D0 to D7 after each Vaccination) . 60
8.3.3.3 Adverse Events from D0 to D21 after each Vaccination 65
8.3.3.4 Transcription of Safety Information to the CRF; Causality Assessment 65
8.3.4 Handling of Missing Data and Outliers 66
9 Serious Adverse Events 67
9.1 Reporting of Serious Adverse Events 67
9.1.1 Initial Reporting by the Investigator 67
9.1.2 Follow-up Reporting by the Investigator 67
9.1.3 Reporting of SAEs Occurring After Subject Trial Termination 68
9.1.4 Causal relationship 68
9.1.5 Reporting SAEs to Health Authorities and IECs/IRBs 68
9.1.6 Additional Information 68
10 Data Collection and Management 69
10.1 Data Collection, CRF Completion 69
10.2 Data Management 69
11 Statistical Methods and Determination of Sample Size 70
11.1 Statistical Methods 71
11.1.1 Hypotheses and Statistical Methods for the Safety Endpoints 71
11.1.1.1 Hypotheses 71
11.1.1.2 Safety Analysis 71
11.1.2 Hypotheses and Statistical Methods for the Immunogenicity Endpoints 72
11.1.2.1 Hypotheses 72
11.1.2.2 Statistical Analysis 72
11.1.2.2.1 Immunogenecity Analysis of the Primary Series Vaccination 72
11.1.2.2.2 Methods for Statistical Analysis 73
11.1.2.2.3 Exploratory Statistical Analyses 73

11.1.2.2.4 Pre-booster Antibody Persistence 73
11.1.2.2.5 Booster Vaccination Immunogenicity 74
11.1.2.2.6 Cellular Mediated Immune Response 74
11.2 Population to be Analyzed 74
11.2.1 Definition of Populations 74
11.2.1.1 Immunogenicity Analysis Set 74
11.2.1.2 Per-Protocol Analysis Set 74
11.2.1.3 Safety Analysis Set 74
11.2.2 Populations Used in Analyses 75
11.3 Determination of Sample Size and Power Calculation 75
11.4 Interim Analysis 75
12 Ethical and Legal Issues and Investigator/Sponsor Responsibilities 76
12.1 Ethical Conduct of the Trial / Good Clinical Practice 76
12.2 Source Documents and Source Data 76
12.3 Confidentiality of Data and Access to Subject Records 76
12.4 Monitoring, Auditing, and Archiving 77
12.4.1 Monitoring 77
12.4.2 Audits and Inspections 78
12.4.3 Archiving 78
12.5 Financial Contract and Insurance Coverage 78
12.6 Stipends for Participation 78
12.7 Publication Policy 78
13 Reference List 79

List of Abbreviations

Ab	Antibody
Ad	Adjuvant
ADR	Adverse Drug Reaction
AE	Adverse Event
AR	Adverse Reaction
CHMP	Committee for Medicinal Products for Human Use
CI	Confidence Interval
CMI	Cellular Mediated Immune
CPMP	Committee for Proprietary Medicinal Products
CRA	Clinical Research Associate
CRF	Case Report Form
D	Day
DC	Diary Card
°C	Degrees Celsius
dil	Dilution
ELISA	Enzyme Linked Immunosorbent Assay
EMEA	European Medicines Agency
FAS	Full Analysis Set
FVFS	First Visit First Subject
FVLS	First Visit Last Subject
GCI	Global Clinical Immunology
GCP	Good Clinical Practice
GM	Geometric Mean
GMT	Geometric Mean Titer
GMTR	Geometric mean of individual titer ratio
GPVD	Global Pharmacovigilance Department
HA	Hemagglutinin
HAU	Hemagglutination unit
HI	Hemagglutination inhibition
IAS	Immunogenicity Analysis Set
ICH	International Conference on Harmonization
IEC	Independent Ethics Committee
IL	Interleukin
ILI	Influenza-like illness
IM	Intramuscular

INFα	Interferon-alpha
IRB	Institutional Review Board
LIMS	Laboratory Information Management Systems
LLOQ	Lower limit of quantification
LVLS	Last Visit Last subject
M	Month
MA	Memory Aid
MedDRA	Medical Dictionary for Regulatory Activities
MDCK	Madin-Darby canine kidney
µL	Microliter
NA	Neuraminidase
NIBSC	National Institute for Biological Standards and Control
NSAID	Non Steroidal Anti-Inflammatory Drugs
OD	Optical Density
PBMC	Peripheral blood mononuclear cell
Pg	Picogram
QA	Quality Assurance
RBC	Red blood cell
SAE	Serious Adverse Event
SafAS	Safety Analysis Set
SN	Seroneutralization
SRH	Single Radial Hemolysis
TNFα	Tumor Necrosis Factor-alpha
UK	United Kingdom
ULOQ	Upper limit of quantification
USA	United States of America
V	Visit
VAC	Vaccination
WHO	World Health Organization

1 Introduction

1.1 Background

Influenza viruses belong to the *Orthomyxoviridae* family and are separated into three types of strains, A, B, and C, according to antigenic differences. Only influenza A and B cause overt disease and result in regular outbreaks or epidemics.

Influenza viruses get their names from two sets of protein spikes that jut from the outer surface of the viruses. The hemagglutinin, or HA, spike governs virus binding and entry into cells, where copies of the virus are produced. The neuraminidase, or NA, spike governs the release of newly formed virus from infected cells into the host's body.

Wild waterfowl, gulls, and shore-birds are the natural reservoir of all influenza A viruses. All 16 HA subtypes (H1-H16) and 9 NA subtypes (N1-N9) have been detected in free-flying birds. While some birds have been symptomatic, others have carried the viruses without developing symptoms and provide a huge and highly mobile pool of genetic diversity. The disease can spread from country to country through migratory birds, including wild waterfowl, sea birds, and shore-birds.

In poultry, avian influenza causes two distinctly different forms of disease. In the common and mild form of avian influenza, signs of illness range from ruffled feathers and reduced egg production to typical respiratory symptoms. In contrast, in the second form, the outbreak of the highly pathogenic strain of avian influenza is characterized by a sudden onset of severe disease and rapid spread, devastating the poultry and farming industry with a mortality that can approach 100% within 48 hours. In addition, porcine infections have been reported.

During the last 8 years, a growing number of Asian countries have reported outbreaks of highly pathogenic avian influenza in chickens and ducks, and infections in several species of wild birds and in pigs have also been reported. Cases of human infection with avian influenza viruses of 3 subtypes (H5, H7 and H9) have been described in individuals who have been in direct contact with infected birds.

For the H7 subtype, an outbreak of avian influenza in poultry farms with a highly pathogenic H7N7 virus caused 89 human infections including one death in the Netherlands in 2003 (1). In British Columbia in 2004 human infections with an H7N3 virus were reported in association with an outbreak of both low and high pathogenic viruses in poultry (2). With several outbreaks of influenza in poultry in Italy between 1999 and 2003, a serological survey was conducted of poultry workers in affected regions. This showed that during the period in 2003 when a low pathogenic avian H7N3 strain was circulating, 3.8% of the associated poultry workers showed evidence of anti-H7 antibodies (3).

In 1997 an outbreak of a highly pathogenic chicken H5N1 virus caused serious human illness in Hong Kong where 6 people died (33% mortality). Two years later a second avian virus (H9N2) transmitted to man causing illness in two children in Hong Kong.

Between December 2003 and mid-July 2006, A/H5N1 viruses have caused at least 230 confirmed cases (including 132 deaths) in human adults and children in Vietnam, Thailand, Indonesia, China, Cambodia, Turkey, Iraq Azerbaijan, Djibouti and Egypt (4). To date, people who have been infected with the A/H5N1 virus have been in direct contact with infected and/or sick birds. However, a bird-flu outbreak in an Indonesian village where seven family members have now died, combined with the fact that the virus may have spread directly from a woman to her nephew and then from the nephew to his father, has raised the level of concern that the virus may be able to pass directly between people. With no animal identified as yet as the source of infection, the family cluster in Indonesia raises the suspicion of human-to-human transmission. This would be the first known three-person chain of human-to-human transmission. The virus has not spread beyond the members of this single extended family.

The clinical spectrum of influenza A (H5N1) in humans is based on descriptions of hospitalized patients. The frequencies of milder illnesses, subclinical infections, and atypical presentations (e.g., encephalopathy and gastroenteritis) have not been determined, but case reports indicate that each occurs. Most patients have been previously healthy young children (40 laboratory-confirmed cases between 0-9 years and 33 in 10-19 years) or adults (40 laboratory-confirmed cases between 20-29 years, 28 in 30-39 years and 10 in 40-49 years).

Avian influenza A (H5N1) infection in humans is characterized by the sudden onset of high fever and an influenza-like illness with lower respiratory tract symptoms. Diarrhea, vomiting, abdominal pain, pleuritic pain, and bleeding from the nose and gums have also been reported early in the course of illness in some patients. Although many patients recover, some die rapidly due to tracheo-bronchitis associated with dyspnoea. The fatality rate among hospitalized patients is high among infants and young children (89% in those younger than 15 years of age in Thailand). Death occurred an average of 9 to 10 days after the onset of illness (range: 6 to 30 days) (5).

While to date the evidence that H5N1 is able to spread from human to human is limited (6), continued circulation of the H5N1 strain, which now has reached levels of endemicity among poultry in several southeast Asian countries increases the possibility that avian and human influenza viruses could exchange genes if an individual was simultaneously infected with viruses from both species (7).

This could give rise to a new antigenic variant (antigenic shift) caused by substitution of the hemagglutinin antigen on the surface of the virus, with or without a concomitant change in neuraminidase, the other surface antigen. If such a virus demonstrates the ability to infect and spread in an immunologically naïve human population, the result is a global outbreak of the disease (a pandemic) that could affect a large number of individuals in a short period of time and is likely to cause substantially increased morbidity and mortality in all countries of the world.

Therefore, pandemic preparedness has become an important worldwide issue. Although anti-viral drugs exist, vaccines will form the main prophylactic measure against pandemic influenza, and will play an important role in the plans to prepare for a pandemic. Such preparation includes stock-piling of antivirals, as well as the development of candidate vaccines by an increasing number of countries worldwide, and alternative methods for rapid vaccine production and dose reduction of vaccines (8) (9) (10) (11).

In 2002, a World Health Organization (WHO) Global Agenda for Influenza Surveillance and Control was adopted, whose main objectives are to enhance surveillance for the identification of emerging viruses, improve knowledge of the disease burden, and improved public health infrastructure to manage and coordinate control efforts to accelerate pandemic prepardness (12). Vaccination is becoming a public health priority and, alongside other measures (such as antiviral medications for prevention and treatment of infections caused by pandemic viruses), will play a key role in the combat against pandemic influenza (13).

In April 2004, the Committe for Medicinal Product for Human Use (CHMP) issued guidelines for licensing pandemic influenza vaccines (14) (15) in order to accelerate the development of the pandemic vaccine. The guidelines recommend the development of a "mock-up" pandemic vaccine or "prototype pandemic influenza vaccine" which should be produced in the same way, have the same antigen content and same adjuvant system (if used) and use the same administration route as the intended pandemic vaccine during the inter-pandemic period. The licensing procedure of this "mock up" vaccine involves the submission and approval of a core pandemic dossier.

In the event of a pandemic with a new viral strain, a fast-track procedure for registration of the final pandemic vaccine will be implemented, based on the submission of a dossier for the pandemic variation.

In view of the occurrence of human H5 virus infection with the highly pathogenic A/Vietnam/1194/2004 strain in recent years, this has been identified as a potential pandemic strain.

In this context, sanofi pasteur's aim through the Flu Pandemic H5N1 Eggs project is to develop a safe and immunogenic monovalent inactivated split influenza virus vaccine produced in eggs according to the Vaxigrip production process, using an H5N1 reassortant derived from the A/Vietnam/1194/2004 strain produced by reverse genetic and provided by a WHO reference center (A/Vietnam/1194/NIBRG-14 [H5N1]) for the core pandemic dossier according to EMEA's program. The pandemic split influenza vaccine may be administered in combination with a well-established adjuvanting system (aluminum hydroxide).

1.2 Subject Benefits / Potential Risks

There are no definite benefits following vaccination in this trial. Subjects may develop an antibody response to the A/Vietnam/1194/NIBRG-14 (H5N1) vaccine, although this can not be guaranteed. Furthermore, in the event that an H5 strain is found in birds or circulates as a pandemic, cross-protection of such an antibody response cannot be guaranteed.

The potential risks of receiving the investigational vaccine are listed in the current Investigator's Brochure. Influenza vaccines are usually well tolerated when injected into a muscle. The most frequently observed injection site reactions in a Phase I adult study, GPA01 (16), were pain, erythema and induration and the most frequently reported solicited systemic reactions in all groups and for each injection were headache and myalgia. Most injection site and systemic reactions generally appeared on the day following vaccination and spontaneously disappeared within 3 days. Additionally, blood sampling can cause pain and bruising at the puncture site.

Since influenza vaccines have been marketed, some other reactions such as itching, urticaria, or redness over the entire body have been observed infrequently (between 1/100 and 1/1 000

vaccinees). Nerve pain, paresthesia, convulsions, and temporary low blood platelet count have occurred rarely (between 1/1 000 and 1/10 000 vaccinees). Very rare reactions such as neurological disorders have been reported (in less than 1 out of 10 000 vaccinees). As with any vaccination, there is a rare possibility of an allergic reaction. This may cause a severe narrowing of the air passages and breathing difficulties.

Data from interpandemic influenza vaccines suggest that other effects may include:

- In rare cases (between 1/100 and 1/1 000 vaccinees): urticaria, pruritus, erythematous rash, dyspnea, leading to shock.
- In very rare cases (between 1/1 000 and 1/10 000 vaccinees): neuralgia, paresthesia, convulsions, transient thrombocytopenia, vasculitis with transient renal involvement.
- In less than 1/10 000 vaccinees, neurological disorders, such as encephalomyelitis, neuritis and Guillain-Barré syndrome. In particular, an increased incidence of Guillain-Barré syndrome was associated with vaccination against swine influenza in the USA in the late 1970s (17).

Aluminum in the form of aluminum hydroxide has been used as an adjuvant in many licensed vaccines. Aluminum has a demonstrated safety profile (over six decades), but has been associated with local reactions such as erythema, subcutaneous nodules and contact hypersensitivity.

Due to the multidose presentation of the vaccine, thimerosal is used as a preservative in the vaccines used in this study. Although there is no evidence of harm caused by the thimerosal in the vaccines, there is a theoretical risk that hypersensitivity (allergic) reactions could be induced (European Medicines Agency [EMEA] public statement of thimerosal in vaccines for human use EMEA/CPMP/VEG/1194/04).

1.3 Rationale

An initial two-study approach has been used for the development of the core pandemic dossier, according to the CHMP Note for Guidance (14). A first, dose-finding study is to be followed by a second, larger study performed in adults and elderly and using two doses selected from the first study (7.5 µg HA without adjuvant and 30 µg HA with adjuvant).

In April 2005, a Phase I, multicenter, randomized, open study (GPA01 (16)) was initiated to test several adjuvanted and non-adjuvanted A/H5N1 vaccine formulations (7.5, 15 and 30 µg with or without aluminum hydroxide as an adjuvant) as a two-dose schedule in immunologically naïve adults aged 18 to 40 years. In this study, 300 subjects received two injections (on D0 and D21) of one of the six investigational products and are currently being followed-up for a total of one year. Immunogenicity 21 days after the first and the second vaccinations has been determined by hemagglutination inhibition (HI) using turkey and horse erythrocytes, single radial hemolysis (SRH) and seroneutralization (SN). Antibody (Ab) persistence is also being evaluated 6 and 12 months after the first vaccination, and a booster is to be administered at 12 months after the first vaccination.

The vaccine appeared to be generally safe, well tolerated, and immunogenic following two administrations of each formulation/dose with solicited injection site and systemic reactions

occurring at a similar frequency to that seen using the interpandemic trivalent inactivated influenza vaccine in terms of the CHMP safety criteria within 3 days post-vaccination.

In conclusion, this previous clinical trial (16) showed that there is a positive benefit-risk ratio (see Section 1.2) for a potential pandemic vaccine, in particular with the 30 µg with adjuvant formulation. In addition, as an immune response was observed in a number of subjects receiving the lowest dose, it is of interest from a dose sparing perspective to further investigate the 7.5 µg without adjuvant dose/formulation (the unadjuvanted rather than the adjuvanted formulation has been selected since the immune response was similar for both and the production of the unadjuvanted formulation will be simpler in an emergency pandemic situation).

The second trial (GPA02 (18)), a Phase II, multicenter, open trial, in a total of 600 subjects (in two vaccine formulation groups [30 µg HA with adjuvant and 7.5 µg HA without adjuvant] and with two age groups [18 to 60 years and >60 years] per vaccine formulation group) started in May 2006 to assess safety and immunogenicity in a larger population than studied in GPA01. As for GPA01, two vaccinations were performed, separated by 21 days, and a booster will be administered at either 6 or 12 months following the first vaccination.

In this second trial, one subset of subjects will receive a booster vaccination with low dosage of the same vaccine strain as those used in the priming vaccination (A/H5N1 Clade 1: A/Vietnam/1194/NIBRG-14) at 6 months after the first vaccination. Another subset of subjects will receive a booster vaccination of either 7.5 µg HA without adjuvant or 30 µg HA with adjuvant of the new vaccine strain (A/H5N1 Clade 2) of the one used in the priming vaccination (A/H5N1 Clade 1: A/Vietnam/1194/NIBRG-14) at 12 months after the first vaccination.

In parallel, 100 additional subjects aged from 18 to 60 years will be enrolled to receive two administrations of the new vaccine strain (A/H5N1 Clade 2) 21 days apart in order to assess the immunogenicity response to priming vaccinations of this new strain.

In a pandemic situation, children may be very vulnerable to infection and they constitute a special target group for vaccination. Therefore, the CHMP guideline recommends that at least limited safety data should be obtained from healthy children to support the initial core pandemic dossier data have been obtained from adults. The dose to be used for young children may be different to those which have been shown to be safe and immunogenic in adults, and so half-doses are also to be used in the present trial in Group 3, as described in the following paragraph.

At the start of the present trial all the data (from both the primary series and the booster vaccination) from GPA01 and the D0 to D42 (primary series) data from GPA02 will be available, and the present trial will investigate the safety and immunogenicity of the selected doses (based on adult data) in children aged between 6 months and 17 years. Subjects will be split into three groups (aged 9 to 17 years [Group 1], 3 to 8 years [Group 2], and 6 to 35 months [Group 3]) and the formulations/doses used will be the same as those used in GPA02, with the exception of two half doses that will be used in Group 3. A step-down design based on age will be used, with Day 0 to 7 safety data being reviewed from the previous group prior to progression to the next age group. The immunogenicity response 21 days after each vaccination and Ab persistence until the booster vaccination at 6 or 12 months will be evaluated by HI and SN as recommended by the CHMP guideline.

HI will be evaluated using horse erythrocytes for all subjects, and in addition the HI analysis may be performed using turkey erythrocytes for subjects in Groups 1 and 2 only. Although the method using turkey erythrocytes is the reference method for immunogenicity assessment for inter-pandemic vaccines, this has not been very sensitive in the previous studies and the use of horse erythrocytes has provided better data due to a higher proportion of α 2,3 Gal receptors (which fix the avian HA) (9).

A booster vaccination will be performed at 6 or 12 months after the first vaccination according to the Ab persistence and booster response results observed in adults. Previous influenza pandemics have spread in two to three waves over a period of 13 to 23 months, with the first wave being less intense than the second, which may occur 3 to 9 months later. A booster vaccination may be required to induce a sufficient quantity of Ab to ensure protection throughout the pandemic period (19); Ab persistence and booster response data in adults will facilitate the assessment of the requirement for, and timing of, a booster vaccination in the present trial.

Rationale for Amendment 1:

Following the protocol review by the Ethic Committee of the Ministry of Public Health (IEC for the Queen Sirikit National Institute of Child Health), some clarifications have been made:

- Vaccine dosage received at the booster time will be determined according to the on-going study in adults,
- Functioning of the D0-D7 safety review,
- In case of related seizure after a vaccination, the subject will not receive further vaccination,
- Reason to perform CMI analysis only in Group 3,
- IEC approval will be obtained in case unused blood samples will be retained for more than 5 years as well as if they will be used for other evaluation studies.

In addition, volume to be injected according to dosage is clarified.

2 Trial Objectives

- To describe the safety profiles (injection site reactions and systemic events) during the 21 days following each injection (including the booster injection).
- To describe the immune response 21 days after the first and the second injection of each formulation.
- To describe the antibody persistence until the time of the booster vaccination at 6 or 12 months[a] after the first vaccination.

---

[a] The timing and dosage of the booster vaccination is dependent on the results of an ongoing study (GPA01). The possibilities of a booster at 6 or 12 months (and two flow-charts) are included as options, although by the time of protocol finalization the data from the ongoing study should be available and so it should be possible to decide on the booster timing and type during the protocol writing.

- To describe the immune response 21 days after a booster vaccination administered at 6 or 12 months after the first vaccination.

2.1 Endpoints

Safety

- Occurrence, nature, (Medical Dictionary for Regulatory Activities [MedDRA]), duration, severity, and relationship to vaccination for any unsolicited systemic adverse events (AEs) reported in the 30 minutes after each injection.
- The occurrence, time to onset, number of days of occurrence, severity, and seriousness of solicited (prelisted in the subject diary and Case Report Form [CRF]) injection site reactions and systemic reactions occurring within 7 days following each injection will be reported (including the booster injection).
- The occurrence, nature (MedDRA preferred term), severity, relationship to vaccination and seriousness of unsolicited (spontaneously reported) adverse events within 21 days following each injection will be reported (including the booster injection).
- The occurrence, nature, time to onset, and relationship to vaccination of serious adverse events (SAEs) during the whole study period will be reported.
- The occurrence of the following reactions (MedDRA Preferred Terms given in parentheses) in the three days following each injection will be more especially reported (as defined by the EMEA Note for Guidance [CPMP/BWP/214/96]) (including the booster injection)[a].
    - Injection site induration >5 cm observed for more than 3 days.
    - Injection site ecchymosis (injection site hemorrhage).
    - Rectal equivalent temperature >38°C for 24 hours or more (pyrexia).
    - Malaise.
    - Shivering (chills).

Immunogenicity

Anti-HA Ab titers against the A/H5N1 strain will be expressed as described below (the assay will be performed using horse erythrocytes for all subjects and using turkey erythrocytes for subjects in Group 1 and Group 2; anti-HA titers will not be measured using turkey erythrocytes for Group 3 due to the extra blood sample volume that would be required):

- HI titer obtained in duplicate on D0, D21, and D42, and summarized at the subject level by individual geometric means (GMs) of duplicates at each timepoint. The following endpoints will be derived:
    - Individual titer ratios D21/D0, D42/D0, and D42/D21.
    - Proportion of subjects with HI titer ≥40 (turkey) or ≥32 (horse) 1/dilution (dil) on D0, D21 and D42.

---

[a] Note that it may be difficult to collect certain of these for Group 3 (e.g. malaise)

- Seroconversion (for subjects with a titer <10 [turkey] or <8 [horse] [1/dil] on D0: post-injection titer ≥40 [turkey] or ≥32 [horse] [1/dil])

or

- Significant increase (for subjects with a titer ≥10 [turkey] or ≥8 [horse] [1/dil]: ≥four-fold increase of the titer) on D21 and D42.

- Seroneutralization (SN) titer obtained in duplicate on D0, D21, and D42 and summarized at the subject level by individual GMs of duplicates at each timepoint. The following endpoints will be derived:
  - Individual titer ratios D21/D0, D42/D0, and D42/D21.
  - Two- and four-fold increase from D0 to D21, from D0 to D42, and from D21 to D42.

Antibody persistence

Anti-HA titer (HI method) and neutralizing Ab titer (SN method) in duplicate at M3 and prior to the booster vaccination at M6 (D180), or at M3 and M6 and prior to the booster vaccination at M12 (D365).

Booster vaccination response

- HI titer obtained in duplicate at V07 (M6) (for 6-month booster design) or V08 (M12) (for 12-month booster design), V08 (M6+21 days) (for 6-month booster design) or V09 (M12+21 days) (for 12-month booster design), and summarized at the subject level by individual GMs of duplicates at each timepoint. The following endpoints will be derived:
  - Individual titer ratios V08/V07 (for 6-month booster design) or V09/V08 (for 12-month booster design).
  - Proportion of subjects with HI titer ≥40 (turkey) or ≥32 (horse) (1/dil) at V07 and V08 (for 6-month booster design)or V08 and V09 (for 6-month booster design).
  - Seroconversion (for subjects with a titer <10 [1/dil] at V07 (for 6-month booster design) or V08 (for 12-month booster design): post-injection titer ≥40 [turkey] or ≥32 [horse] [1/dil]) at V08 (for 6-month booster design) or V09 (for 12-month booster design),

  or

  - Significant increase (for subjects with a titer ≥10 [1/dil]: ≥four-fold increase of the titer) at V08 (for 6-month booster design) or V09 (for 12-month booster design).
- SN titer obtained in duplicate at V07 and V08 (for 6-month booster design) or V08 and V09 (for 12-month booster design) and summarized at the subject level by individual GMs of the duplicates at each timepoint. The following endpoints will be derived:
  - Individual titer ratios V08/V07 (for 6-month booster design) or V09/V08 (for 12-month booster design).
  - Two- and four-fold increase from V07 to V08 (for 6-month booster design) or V08 to V09 (for 12-month booster design).

2.2 Summary of Statistical Methodology for the Objectives

The analyses will be mainly descriptive.

- The safety analysis will describe the incidence of solicited reactions within 7 days after each injection for each injection and for the pooled data from both injections, and of unsolicited AEs within 21 days after each injection for each injection and for the pooled data from both injections. The 95% confidence intervals (CI) of point estimates of pre-defined solicited reactions will be calculated using the exact binomial distribution (Clopper-Pearson method) for proportions. Additionally, for each injection, the safety data will be presented according to the CHMP safety criteria.
- For the immunological parameters, 95% CIs of GM of titers at each timepoint and GM of titers ratios between timepoints will be calculated using normal approximation of the Log_{10}-transformed titers and then back-transformation (primo and booster vaccinations). Additionnaly, the proportion of subjects with HI titer ≥40 (turkey) or ≥32 (horse) (1/dil) and seroconversion (or significant increase) rate will be presented for HI titers and two- and four-fold increases will be presented for SN. 95% CIs for these rates will be calculated using the exact binomial distribution (Clopper-Pearson method) for proportions. HI and SN antibody titers will also be presented by frequency.

2.3 Observational Objective

- To describe in Group 3 the cellular mediated immune (CMI) response before the first vaccination and 8 days after the second vaccination

3 Investigators and Trial Organization

The trial will be conducted at two sites in Thailand under the responsibility of two Principal Investigators, Associate Prof. Tawee CHOTPITAYASUNONDH, M.D (Coordinating Investigator) and Prof. Usa THISYAKORN, M.D.

The complete list of Investigators and other personnel involved in the trial, and the trial site locations is provided in Appendix 1.

The two Principal Investigators will be the Signatory Investigators for the Final Clinical Trial Report, and the Sponsor's Responsible Medical Officer (person authorized to sign this protocol and any amendments on behalf of the Sponsor) is PD Dr. med. Josef WEIGL, Clinical Team Leader.

4 Independent Ethics Committee (IEC)/Institutional Review Board (IRB)

Before the investigational product can be shipped to the investigational site and before the inclusion of the first subject, this protocol, the written informed consent forms, consent form

updates, subject recruitment procedure (e.g. advertisement) and any other written information to be provided to subjects/parent(s)/legal representative, must be approved by, or receive favorable opinion from, the appropriate IEC(s)/IRB(s).

Each Investigator is responsible for obtaining this approval or favorable opinion before the start of the trial and any subsequent amendments in compliance with Good Clinical Practice (GCP) and local regulations. Copies of these approvals, along with information on the type, version number and date of document, and the date of approval, must be forwarded by the Investigator to the Sponsor together with the composition (names and qualifications of the members attending and voting at the meetings) of the IEC/IRB.

The Coordinating Investigators will submit written summaries of the status of the trial to the IEC/IRB annually, or more frequently if requested. All serious adverse reactions occurring during the trial will be reported to the IEC/IRB, according to IRB policy.

5 Investigational Plan

5.1 Description of the Overall Trial Design and Plan

5.1.1 Trial Design

The trial is a Phase II, open, randomized, multi-center trial that will be conducted in 240 subjects in three groups. Sixty subjects aged 9 to 17 years (i.e. ≥9 to <18 years), 60 subjects aged 3 to 8 years (i.e. ≥3 to <9 years) and 120 subjects aged 6 to 35 months (i.e. ≥6 to <36 months) will be enrolled in a step-down design, with subjects being enrolled only after a satisfactory review of the Day 0 to 7 safety data from the previous group, as applicable.

Each subject will receive a primary series of two vaccinations (same dose for both vaccinations) separated by 21 days, and a booster vaccination at either 6 or 12 months after the first vaccination. The timing of the booster vaccination is to be determined after the results of previous trials (GPA01 and GPA02) are available. The dosage to be given as booster could be different from the primary series based on new data available from on going study on these two formulations in adult age >18 years. The dosage to be received will be submitted for approval to IECs.

5.1.2 Trial Plan

The trial plan is summarized in Figure 1 (Trial Flow-Chart) and in Sections 1.3 and 5.1.3.

Recruitment and information of subjects:

Before the inclusion, the Investigator may orally inform potentially eligible subjects or their parents, as applicable, about the trial. They may be given an oral description of the trial design, presenting the general benefits and discomforts related to the trial. They may be informed that they may return and receive further information and sign the full informed consent during the recruitment period. The process of subject recruitment and any oral or written information that will be provided to the subjects must be documented. This will be available in the Investigator's file and the Trial Master File. It is highly recommended to document in the subject's medical file

(source documents) the transmission of oral information to the subject or parent at the time of a medical visit to the Investigator.

It should be noted that the subjects who may have been pre-screened may not necessarily be included in the trial if the required number of subjects has already been recruited.

Trial description:

After the Informed Consent Form (ICF) (to be signed by parent/legal representative for all subjects) and assent form (one for subjects aged 7 to 12 years [i.e. ≥7 to <13 years] and another for subjects aged 13 to 17 years [i.e. ≥13 to <18 years]) have been signed, eligible subjects will be randomized to receive primary vaccinations of 7.5 µg HA without adjuvant or 30 µg HA with adjuvant (Group 1 [9 to 17 years] and Group 2 [3 to 8 years]) or 3.75 µg HA without adjuvant, 7.5 µg HA without adjuvant, 15 µg HA with adjuvant or 30 µg HA with adjuvant (Group 3 [6 to 35 months]).

Both formulations (30µg HA + Ad and 7.5µg HA) are well tolerated with no safety signals raised following administration of a first and second dose in adults and the elderly subjects. Based on the available data, no safety concerned is anticipated in children. However, as a precaution, subjects will only be enrolled after a satisfactory review of the Day 0 to 7 safety data for the previous vaccination and/or the previous age group. This process is represented in the following diagram:

Figure 3: Diagram to Show the Safety Data Review Process Prior to Inclusion of Subsequent Groups

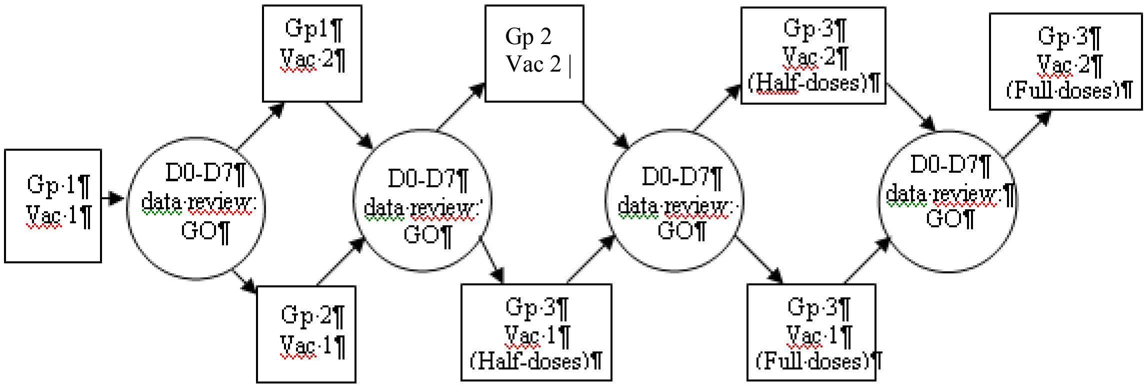

Sanofi pasteur safety committee will perform a formal review of safety within 7 days after the first vaccination of each cohort of subjects. The 2nd vaccination of the cohort and the first vaccination of the next cohort of age will depend on the recommendation given by sanofi pasteur after discussion with the Principal Investigators. Safety reports and conclusion from the sanofi pasteur safety committee will be forwarded to the IECs after each go/no go decision.

During the safety review meeting, sanofi pasteur safety committee will evaluate each SAE and examine the severity, duration of symptoms and the causal relationship according to the Investigator assessment. In addition, the safety committee will also focus on severe fever.

The safety profile will be compared to that observed in adult population.

This review might be escalated to the Product Safety Board for scientific and medical advice on any severe medical event in case significant findings are pointed out following safety review.

The safety committee will share the conclusions of safety review with the Principal Investigators. Within 24h following the meeting, the safety committee will send to the Principal Investigators and to the IECs the main conclusions of the meeting, and especially the decision for next cohort vaccination.

Then, the sanofi pasteur safety committee will provide a comprehensive report on safety data collected with its recommendations to Principal Investigators and IECs to pursue or not the vaccinations.

In case of trial modification/discontinuation, a meeting will be organized between the Principal Investigators and the Sponsor, to review recommendations and decide how the trial can be modified (modification of the subject follow-up, pause/stop the trial).

A booster vaccination will be performed at 6 or 12 months[a] after the first vaccination. Each subject will be followed up for 6 months after the booster vaccination, and will provide six, seven or eight blood samples (see Figure 1 and Figure 2).

Subjects will be observed for safety for 30 minutes after each vaccination, and any AE/reaction occurring during the 21 days after each vaccination will be recorded using a Diary Card (DC). In addition, all SAEs will be recorded during the entire trial (including the 6 month follow-up period).

Immunogenicity will be evaluated 21 days after the first and second vaccinations, prior to the booster vaccination and 21 days after the booster vaccination. HI will be assessed using horse erythrocytes (for all subjects) and using turkey erythrocytes (for subjects in Groups 1 and 2 only). SN will be assessed in all subjects, and the CMI response will be assessed for subjects in Group 3 only.

The cell mediated immune system in children aged more than 3 years is usually more mature and similar to that in adults (20) (21) (22). As CMI response in adults has been evaluated in previous trials, CMI was chosen to be studied in subjects aged 6 – 35 months.

5.1.3 Visit Procedures

V01: First vaccination (D0) and blood sampling:

- The Investigator or delegate will:

1) Obtain informed consent and/or assent (as described in Section 5.1.2) by presenting the trial to the subject and/or subject's parent/legal representative and/or independent witness, as applicable, answer any questions, and ensure that the subject and/or subject's parent/legal representative has been informed of all aspects of the trial that are relevant to the decision to participate.
2) Date and sign the ICF, after it has been dated and signed by the subject and/or subject's parent/legal representative and/or independent witness, as applicable (the Investigator will retain the original of this document and will give the copy to the subject or subject's parent/legal representative).
3) Take the subject's axillary temperature and record in the source document and CRF.
4) Perform a urine pregnancy test for subjects of child-bearing potential (as applicable).
5) Check all inclusion and exclusion criteria through physical examination and medical interview.
6) Allocate a trial inclusion number to the subject (see Section 6.6).
7) Record the subject's initials, subject number, date of birth, height, weight, sex, ethnic origin (Caucasian, Asian, Black, or other), past and current significant medical history, and all information related to points 3 to 5 (above) in the source document and then in the CRF.

---

a The timing and type of booster to be determined following results from GPA01 and GPA02

8) Record details of any influenza-like illness (ILI) and inter-pandemic influenza vaccination (including dates of occurrence) in the CRF.
9) Take a blood sample in a dry silicone tube from all subjects for serology evaluation (7 mL for Groups 1 and 2; 2 mL for Group 3). Take a further 2 mL sample in a sodium heparin tube from subjects in Group 3 only for CMI response analysis.
10) If blood sampling for serology cannot be performed, the subject will not be vaccinated. In this case, his/her subject number will not be reassigned.
11) Scratch the randomization list to define the vaccine dose corresponding to the subject's number.
12) Administer the vaccine dose corresponding to the subject's treatment group, and preferably on the side opposite to that of blood sampling (for subjects receiving the vaccination in the deltoid muscle only – see Section 6.2.2).
13) Affix the corresponding detachable label in the source document and in the CRF.
14) Ensure the 30-minute observation period following vaccination is observed.
15) Complete the source document and CRF.
16) Provide the subject and/or subject's parent/legal representative with the DC1 to be filled in until V03 (and checked at V02), a digital thermometer and a ruler, and instruct the subject how to use these tools and how to complete the first DC.
17) Remind the subject and/or subject's parent/legal representative to contact the Investigator in the event of any SAE between V01 and V02.
18) Make an appointment 8 days (+ 1 day maximum) after the first vaccination.

V02: Safety follow-up visit (D8):

- The Investigator or delegate will:

1) Interview the subject and/or subject's parent/legal representative, requesting information concerning any SAE that may have occurred since V01.
2) Perform a physical examination and medical interview based on the information contained in DC1 and complete the source document.
3) Review DC1 filled in by the subject and/or subject's parent/legal representative since V01 and record any additional information and/or discrepancies in the source documents.
4) Record any solicited reactions/events and prohibited concomitant medications and any analgesics/antipyretics/non-steroidal anti-inflammatory drugs (NSAIDs) taken (see Section 6.8) in the CRF.
5) Remind the subject and/or subject's parent/legal representative to contact the Investigator in the event of any SAE between V02 and V03.
6) Make an appointment 21 days ± 3 days after the first vaccination.

V03: Second vaccination (D21) and blood sampling:

- The Investigator or delegate will:

1) Interview the subject and/or subject's parent/legal representative, requesting information concerning any SAE that may have occurred since V02.
2) Collect and review DC1 filled in by the subject and/or subject's parent/legal representative since D8 and record any additional information and/or discrepancies in the source documents.
3) Perform a physical examination and medical interview based on the information contained in DC1 and complete the source document.
4) Record any unsolicited injection site reactions, systemic reactions/events, and prohibited concomitant medications and any analgesics/antipyretics/NSAIDs taken (see Section 6.8) in the CRF.
5) Take the subject's axillary temperature and record in the source document and CRF.
6) Perform a urine pregnancy test for subjects of child-bearing potential (as applicable).
7) Check all temporary and definite contraindications through physical examination and medical interview (in the event of temporary contraindications, the vaccination will be delayed; in the event of definite contraindications, the vaccination will not be performed).
8) Take a blood sample in a dry silicone tube from all subjects for serology evaluation (7 mL for Groups 1 and 2; 2 mL for Group 3).
9) Administer the vaccine dose corresponding to the subject's treatment group, and preferably on the side opposite to that of blood sampling (for subjects receiving the vaccination in the deltoid muscle only – see Section 6.2.2).
10) Affix the corresponding detachable label in the source document and CRF.
11) Ensure the 30-minute observation period following vaccination is observed.
12) Complete the source document and CRF.
13) Provide the subject and/or subject's parent/legal representative with DC2 to be filled in until V05.
14) Remind the subject and/or subject's parent/legal representative to contact the Investigator in the event of any SAE between V03 and V04.

Make an appointment 8 days (+ 1 day maximum) after the second vaccination.

V04: Safety follow-up visit for all groups and blood sampling for Group 3 only (D29):

- The Investigator or delegate will:

1) Interview the subject and/or subject's parent/legal representative, requesting information concerning any SAE that may have occurred since V03.
2) Perform a physical examination and medical interview based on the information contained in DC2 and complete the source document.
3) Review DC2 filled in by the subject and/or subject's parent/legal representative since V03 and record any additional information and/or discrepancies in the source documents.
4) Examine the subject and interview the subject and/or subject's parent/legal representative and record new findings in the source documents.

5) Record any solicited reactions/events and prohibited concomitant medications and any analgesics/antipyretics/NSAIDs taken (see Section 6.8) in the CRF.
6) Take a blood sample (2 mL) in a sodium heparin tube from subjects in Group 3 only for response analysis.
7) Remind the subject and/or subject’s parent/legal representative to contact the Investigator in the event of any SAE between V04 and V05.
8) Make an appointment 21 days ± 3 days after the second vaccination.

V05: Safety follow-up visit and blood sampling (D42):

- The Investigator or delegate will:

1) Interview the subject and/or subject’s parent/legal representative, requesting information concerning any SAE that may have occurred since V04.
2) Collect and review DC2 filled in by the subject since D21 and record any additional information and/or discrepancies in source documents.
3) Perform a physical examination and medical interview based on the information contained in DC2 and complete the source document.
4) Record any unsolicited injection site reactions, systemic reactions/events, and prohibited concomitant medications and any analgesics/antipyretics/NSAIDs taken (see Section 6.8) in the CRF.
5) Take a blood sample in a dry tube from all subjects for serology evaluation (7 mL for Groups 1 and 2; 2 mL for Group 3).
6) Complete the source document and CRF.
7) Provide the subject and/or subject’s parent/legal representative with Memory Aid 1 (MA1) to be used as necessary until V07.
8) Complete and sign the primary series termination record in the CRF.
9) Make an appointment for the next visit (M3).

V06: Safety follow-up visit and blood sampling (M3):

- The Investigator or delegate will:

1) Interview the subject and/or subject’s parent/legal representative, requesting information concerning any SAE that may have occurred since V05.
2) Perform a physical examination and medical interview based on the information contained in MA1 and complete the source document.
3) Review MA1 filled in by the subject and/or subject’s parent/legal representative since V05 and record any additional information and/or discrepancies in the source documents.
4) Take a blood sample in a dry silicone tube from all subjects for serology evaluation (7 mL for Groups 1 and 2; 2 mL for Group 3).
5) Complete the source document and CRF.

6) Make an appointment for the next visit (D180 days ± 8 days).

----IF BOOSTER AT 6 MONTHS----

V07: Booster vaccination and blood sampling (D180, M6):

- The Investigator or delegate will:

1) Interview the subject and/or subject's parent/legal representative, requesting information concerning any SAE that may have occurred since V06.
2) Collect and review MA1, used by the subject and/or subject's parent/legal representative since V05 and record any additional information and/or discrepancies in source documents.
3) Take the subject's axillary temperature and record in the source document and CRF.
4) Perform a urine pregnancy test for subjects of child-bearing potential (as applicable).
5) Check all temporary and definite contraindications through physical examination and medical interview (in the event of temporary contraindications, the vaccination will be delayed; in the event of definite contraindications, the vaccination will not be performed).
6) Perform a physical examination and complete the source document.
7) Record details of any ILI and inter-pandemic influenza vaccination (including dates of occurrence) in the CRF.
8) Take a blood sample in a dry silicone tube from all subjects for serology evaluation (7 mL for Groups 1 and 2; 2 mL for Group 3).
9) Scratch the randomization list to define the booster dose corresponding to the subject's number (if applicable)[a].
10) Administer the vaccine dose corresponding to the randomization (if applicable) and preferably on the side opposite to that of blood sampling (for subjects receiving the vaccination in the deltoid muscle only – see Section 6.2.2).
11) Affix the corresponding detachable label in the source document.
12) Ensure the 30-minute observation period following vaccination is observed.
13) Complete the source document and CRF.
14) Record any injection site reactions, systemic reactions/events, and prohibited concomitant medications and any analgesics/antipyretics/NSAIDs taken (see Section 6.8) in the CRF.
15) Provide the subject and/or subject's parent/legal representative with the DC3 to be filled in until V08, a digital thermometer and a ruler, and instruct the subject how to use these tools and how to complete the DC.
16) Remind the subject and/or subject's parent/legal representative to contact the Investigator in the event of any SAE between V07 and V08.
17) Make an appointment 21 days ± 3 days after the booster vaccination.

---

[a] If more than one type of booster is used, this is to be defined following results from GPA01 and GPA02.

V08: Blood sampling (M6 + 21 days):

- The Investigator or delegate will:

1) Interview the subject and/or subject's parent/legal representative, requesting information concerning any SAE that may have occurred since V07.
2) Collect and review DC3 filled in by the subject since V07 and record any additional information and/or discrepancies in source documents.
3) Perform a physical examination and medical interview based on the information contained in the DC3 and complete the source document.
4) Record any injection site reactions, systemic reactions/events, and prohibited concomitant medications and any analgesics/antipyretics/NSAIDs taken (see Section 6.8) in the CRF.
5) Take a blood sample in a dry silicone tube from all subjects for serology evaluation (7 mL for Groups 1 and 2; 2 mL for Group 3).
6) Complete the source document.
7) Provide the subject and/or subject's parent/legal representative with MA2 to be used as necessary until the 6-month follow-up.
8) Complete and sign the termination record in the CRF.
9) Make an appointment for the 6-month follow-up visit/phone call (D365 ± 15 days).

6 month follow-up visit/phone call (D365 ± 15D)

The Investigator or delegate will:

1) Make at least 3 telephone calls (or arrange for a visit at the investigational site if it is not possible to contact the subject and/or subject's parent/legal representative by telephone), documented in the source documents; at least 6 months after the last injection, to collect information on SAEs.
2) In the event of an SAE, arrange a visit with the subject to obtain further information.
3) Collect and/or review MA2, used by the subject and/or subject's parent/legal representative since V08 and record any additional information and/or discrepancies in source documents.

----IF BOOSTER AT 12 MONTHS----

V07: Blood sampling (D180, M6):

- The Investigator or delegate will:

1) Interview the subject and/or subject's parent/legal representative, requesting information concerning any SAE that may have occurred since V06.
2) Collect and review MA1, used by the subject and/or subject's parent/legal representative since V05 and record any additional information and/or discrepancies in source documents.
3) Perform a physical examination and complete the source document.
4) Take a blood sample in a dry silicone tube from all subjects for serology evaluation (7 mL for Groups 1 and 2; 2 mL for Group 3).

5) Complete the source document and CRF.
6) Provide the subject and/or subject's parent/legal representative with MA2 to be used as necessary until V08.
7) Make an appointment for the next visit (D365 ± 15 days).
8) Complete and sign the termination record in the CRF.

V08: Booster vaccination and blood sampling (D365, M12):

- The Investigator or delegate will:

1) Interview the subject and/or subject's parent/legal representative, requesting information concerning any SAE that may have occurred since V07.
2) Collect and review MA2, used by the subject and/or subject's parent/legal representative since V07 and record any additional information and/or discrepancies in source documents.
3) Perform a urine pregnancy test for subjects of child-bearing potential only.
4) Check all temporary and definite contraindications through physical examination and medical interview (in the event of temporary contraindications, the vaccination will be delayed; in the event of definite contraindications, the vaccination will not be performed).
5) Perform a physical examination and complete the source document.
6) Record details of any influenza-like illness (ILI) and inter-pandemic influenza vaccination (including dates of occurrence) in the CRF.
7) Take a blood sample in a dry silicone tube from all subjects for serology evaluation (7 mL for Groups 1 and 2; 2 mL for Group 3).
8) Take the subject's axillary temperature and record it in the source document and CRF.
9) Scratch the randomization list to define the booster dose corresponding to the subject's number (if applicable)[a].
10) Administer the vaccine dose corresponding to the randomization (if applicable) and preferably on the side opposite to that of blood sampling (for subjects receiving the vaccination in the deltoid muscle only – see Section 6.2.2).
11) Affix the corresponding detachable label in the source document and CRF.
12) Ensure the 30-minute observation period following vaccination is observed.
13) Complete the source document and CRF.
14) Record any injection site reactions, systemic reactions/events, and prohibited concomitant medications and any analgesics/antipyretics/NSAIDs taken (see Section 6.8) in the CRF.
15) Provide the subject and/or subject's parent/legal representative with the DC3 to be filled in until V09, a digital thermometer and a ruler, and instruct the subject how to use these tools and how to complete the DC.

[a] If more than one type of booster is used, this is to be defined following results from GPA01 and GPA02.

16) Remind the subject and/or subject's parent/legal representative to contact the Investigator in the event of any SAE between V08 and V09.

17) Make an appointment 21 days ± 3 days after the booster vaccination.

V09: Blood sampling (M12 + 21 days]):

- The Investigator or delegate will:

1) Interview the subject and/or subject's parent/legal representative, requesting information concerning any SAE that may have occurred since V08.
2) Collect and review DC3 filled in by the subject and/or subject's parent/legal representative since V08 and record any additional information and/or discrepancies in source documents.
3) Perform a physical examination and medical interview based on the information contained in the DC3 and complete the source document.
4) Record any injection site reactions, systemic reactions/events, and prohibited concomitant medications and any analgesics/antipyretics/NSAIDs taken (see Section 6.8) in the CRF.
5) Take a blood sample in a dry silicone tube from all subjects for serology evaluation (7 mL for Groups 1 and 2; 2 mL for Group 3).
6) Complete the source document and CRF.
7) Provide the subject and/or subject's parent/legal representative with MA3 to be used as necessary until the 6-month follow-up.
8) Complete and sign the termination record in the CRF.
9) Make an appointment for the 6-month follow-up visit/phone call (D545 ± 15 days).

6 month follow-up visit/phone call (D545):

- The Investigator or delegate will:

1) Make at least 3 telephone calls (or arrange for a visit at the investigational site if it is not possible to contact the subject and/or subject's parent/legal representative by telephone), documented in the source documents; at least 6 months after the last injection, to collect information on SAEs.
2) In the event of an SAE, arrange a visit with the subject to obtain further information.
3) Collect and/or review MA3, used by the subject/parent/guardian since V09 and record any additional information and/or discrepancies in source documents.

5.1.4 Planned Trial Calendar

The calendar presented in the following table is indicative. The real dates may differ as, for example, the trial will not start until all the appropriate regulatory and ethical approvals have been obtained.

Table 5.1: Planned Trial Calendar for 6 and 12 Month Booster Designs

	**6 month booster design**	**12 month booster design**
**Planned trial period (FVFS* to LVLS*)**	June 07 to September 08	June 07 to March 09
**Planned Inclusion period (FVFS* to FVLS*)**	June 07 to September 07	June 07 to September 07
**Planned primary vaccination period**	June 07 to September 07	June 07 to September 07
**Planned booster vaccination period**	December 07 to March 08	June 08 to September 08
**Planned end of trial†**	September 08	April 09
**Planned date of final clinical trial report**	November 08	May 09
**Expected trial duration for each subject**	12 months	18 months

* First visit of first subject; LVLS: Last visit of last subject; FVLS: First visit of last subject (last first visit).

† End of trial is defined as the date of the last contact with a trial subject within the scope of the trial.

5.2 Discussion of Trial Design

In preparation for an influenza pandemic, the CHMP has developed licensing guidelines for pandemic influenza vaccines (14) (15). These guidelines describe an initial two-study approach for the development of a core dossier to facilitate the granting of a provisional license in the event that a pandemic occurs. The first study in this core dossier is a dose/formulation finding and the second is a larger study performed in adults and the elderly using dose(s) selected from the first study. These two studies are ongoing and will be included in the initial dossier as required by the CHMP guidelines.

Since children may be very vulnerable to infection, the CHMP guidelines also suggest that at least limited safety data should be obtained from healthy children to support the initial dossier of adult/elderly data. The present study has therefore been designed and will be conducted to address safety in this population and to gain the first information regarding the immune response of children to the dosages/formulations to be tested.

As such, this is a Phase II study, in which three age-groups (9 to 17 years, 3 to 8 years, and 6 to 35 months) will be studied. For safety reasons, groups will be studied in reverse order of age, with a thorough and satisfactory review of the Day 0 to 7 safety data for each primary series vaccination in Group 1 and Group 2 data being performed prior to the inclusion of subjects in Group 2 and Group 3, respectively. This safety review will be performed by the Sponsor’s Clinical Team and will focus on clinically relevant events (severe fever in the 7 days following vaccination and SAEs). These data (excluding SAEs) will be provided unclean as a data listing by the Trial Data Manager and the SAEs will be provided by the Sponsor's Global Pharmacovigilance Department, and will be shared with the Investigators. The review will only be

escalated to a Product Safety Board if there are significant findings following the Clinical Team review.

The age groups chosen were based on previous influenza studies performed by the Sponsor and the inter-pandemic influenza guidelines (7), and the formulations/doses were chosen based on the previous (GPA01 and GPA02) trials. The dose levels and formulations to be used in the primary series of the present trial were chosen using the results of a first, formulation/dose-finding study (GPA01) (16). In addition, half-doses will be administered to subjects in Group 3 as these dose levels could potentially be used in practice in this age group as is the case for the inter-pandemic vaccine. The timing and type of booster to be used in the present trial will be decided following review of the data from GPA01 and GPA02. Thailand was chosen as the country in which to perform the present trial as it is likely that children in this geographical region have been previously infected with H5N1 avian strains and it is a region for a source of potential pandemic influenza virus.

Although the blood sample volume in subjects aged 6 to 35 months (Group 3) will be restricted to 2 mL due to the young age of these subjects, it is planned that subjects in Groups 1 and 2 provide a blood sample volume of 7 mL. The extra volume provided by subjects in Groups 1 and 2 could, following informed consent and approval of IEC, be used in other evaluation studies, e.g. studies of the immune response to influenza vaccines or cross-reactivity with other H5N1 vaccine strains circulating, although no genetic tests will be performed using these samples.

The CMI response will only be evaluated in Group 3 as the evaluation in adults has been included in previous protocols and subjects in Groups 1 and 2 would be expected to show a similar response to that observed in adults.

5.3 Selection of Trial Population

5.3.1 Recruitment Procedures

Recruitment procedures and materials will be submitted for Ethics Committee (EC) approval before implementation.

5.3.2 Participant Information and Consent

Informed consent is the process by which a subject/parent/legal representative voluntarily confirms his or her willingness to participate in a particular trial, after being informed of all relevant aspects of the trial. Informed consent is documented by means of a written, signed and dated ICF.

Written informed consent must be provided for each subject before being included in the trial. For all subjects, an ICF will be signed by one parent/legal representative and by an independent witness if the parent/legal representative is illiterate. In addition, one separate assent form will be signed by subjects aged between 7 and 12 years and another will be signed by subjects aged between 13 and 17 years.

Prior to signing the assent/consent form, the subject/parent/legal representative must be informed by the appropriate study personnel about the nature and purpose of the trial in accordance with GCP and must have sufficient time and opportunity to ask any questions.

In addition, if new information becomes available that may be relevant to the subject's willingness to continue participation in the trial, this will be communicated in a timely manner to the subject. The communication of this information will be provided and documented via a revised consent form or addendum to the original consent form.

A sample ICF (for all subjects) and a sample assent forms (for subjects aged 7 to 12 years and subjects aged 13 to 17 years) are provided in Appendix 2.

ICFs and assent forms will be provided in duplicate (the original will be kept by the Investigator and the copy kept by the subject and/or the subject's legally acceptable representative).

5.3.3 Inclusion Criteria

All subjects:

1) Able to attend all scheduled visits and to comply with all trial procedures.

Children/Adolescents aged ≥2 years to <18 years:

2) Aged ≥2 years to <18 years on the day of inclusion.
3) Informed Consent Form signed by one parent or another legal representative and by an independent witness if the parent/legal representative is illiterate. In addition, two assent forms signed: one signed by subjects aged between 7 and 12 years and the other signed by subjects aged between 13 and 17 years.
4) For a female, inability to bear a child or negative urine pregnancy test (as applicable)[a].
5) For a female of child-bearing potential, use of an effective method of contraception or abstinence for at least 4 weeks prior to the first vaccination and at least 4 weeks after the last vaccination (as applicable)[b].

Infants/toddlers aged ≥6 months to <2 years:

6) Aged ≥6 months to <2 years on the day of inclusion.
7) Born at full term of pregnancy (≥37 weeks) with a birth weight ≥2 kg.
8) Informed consent form signed by one parent/legally acceptable representative and an independent witness if the parent/legally acceptable representative is illiterate.
9) Subject who completed vaccination according to the national immunization schedule.

---

[a] To be considered as unable to bear a child, a subject must be pre-menauposal or have undergone surgical sterilization (tubal ligation or hysterectomy).

[b] Effective methods of contraception include oral contraception (pill), Intra-Uterine Device, diaphragm or condoms used in conjunction with sponge, contraceptive foam or cream, hormonal implants, or transdermal patch.

5.3.4 Exclusion Criteria

All subjects:

1) Participation in another clinical trial in the 4 weeks preceding the first trial vaccination[a].
2) Planned participation in another clinical trial during the present trial period.
3) Congenital or acquired immunodeficiency, immunosuppressive therapy such as anti-cancer chemotherapy or radiation therapy within the preceding 6 months, or long-term systemic corticosteroid therapy[b].
4) Systemic hypersensitivity to any of the vaccine components or history of life-threatening reaction to the trial vaccine or a vaccine containing the same substances (egg proteins, chick proteins, thimerosal, aluminum, neomycin, formaldehyde, and octoxinol 9)[c].
5) Chronic illness at a stage that could interfere with trial conduct or completion[d].
6) Blood or blood-derived products received in the past 3 months.
7) Any vaccination in the 4 weeks preceding the first trial vaccination.
8) Vaccination planned in the 4 weeks following any trial vaccination.
9) History of the H5N1 infection (confirmed either clinically, serologically or virologically).
10) Known HIV, Hepatitis B (HBsAg) or Hepatitis C seropositivity.
11) Previous vaccination with an avian flu vaccine.
12) Subject at high risk[e] of the H5N1 infection during the trial.
13) Thrombocytopenia or bleeding disorder contraindicating IM vaccination.
14) Febrile illness (rectal equivalent temperature ≥38.0°C) on the day of inclusion.

Children/Adolescents aged ≥2 years to <18 years:

15) Breast-feeding mothers.
16) Current alcohol abuse or drug addiction that may interfere with the subject's ability to comply with trial procedures (if applicable, depending on the subject's age).

Infants/toddlers aged ≥6 months to <2 years:

17) History of seizures.

---

[a] Participation in another clinical trial investigating a vaccine, a drug, a medical device, or a medical procedure.

[b] Systemic corticosteroid therapy (prednisolone or equivalent) for more than two consecutive weeks within the past 3 months.

[c] The list of vaccine components is included in the Investigator's Brochure.

[d] Chronic illness may include, but is not limited to, cardiac, renal or auto-immune disorders or diabetes.

[e] Subject likely to be in reccurrent close contact with poultry (including back-yard chickens and fighting cocks) or wild birds.

5.3.5 Temporary Contraindications

Should the following condition occur, the Investigator will postpone the second or booster vaccination until the condition is resolved.

- Acute febrile illness within the 72 hours preceding the vaccination or rectal equivalent temperature ≥38.0°C on the day of the vaccination.

Vaccination should be postponed within the timeframe for vaccination indicated in the trial flowchart (see Figure 1 and Figure 2).

For example, in case a subject experiences fever (rectal equivalent temperature ≥38°C) 75 hours before vaccination until 2 hours before vaccination, this subject complies with the temporary contraindication (fever within 72 hours preceding the vaccination). Vaccination will be postponed until there is no fever for >72 hours.

5.3.6 Removal of Participants from Treatment or Assessment

Withdrawn subjects will not be replaced.

5.3.6.1 Conditions for Withdrawal

A subject may decide or a subject's parent/legal representative may decide for the subject to stop participating in the trial for any reason and at any time. In this case, the Investigator must determine that the reason for withdrawal is not related to an AE. The reason for a subject withdrawing during the trial will be specified in the source document and in the relevant section of the CRF.

Subjects/parents/legal representatives will be informed that they have the right to withdraw from the trial at any time.

The Investigator will discontinue the second or booster vaccination in the case of an event considered to be a definite contraindication (see following list). The vaccination will be delayed in the event of a temporary contraindication (see Section 5.3.5).

Definite Contraindications

The following contraindications will be verified before the second and booster vaccinations:

1) Subject allergic to one of the constituents of the vaccine (particularly egg and chicken protein).
2) Blood or blood derived products received in the past 3 months or ongoing at the visit.
3) Immunosuppressive therapy such as anti-cancer chemotherapy or radiation therapy in the past 6 months or ongoing at the visit, or long-term systemic corticosteroid therapy (for more than two consecutive weeks in the past 3 months before the visit).
4) Pregnancy (positive urine test).
5) Vaccination other than the trial vaccine 4 weeks prior to and 4 weeks after any administration of the trial vaccine.

6) A SAE related to the trial vaccine following a vaccination.

5.3.6.2 Lost to Follow-up Procedures

In the case of subjects who fail to attend a follow-up visit, documented reasonable effort (e.g. documented telephone calls and certified mail) should be undertaken to locate or recall the subject, or at least to determine his/her health status while fully respecting his/her rights. These efforts (e.g. letter, telephone calls, home-visit) should be documented in the subject's source document and CRF.

5.3.6.3 Termination Classification

The Investigator will classify the termination status of each subject at the end of the trial in the termination page of the CRF according to the following:

- SAE.
- Other AE.
- Non-compliance with protocol.
- Lost to follow up.
- Voluntary withdrawal not due to an AE[a].

5.3.6.4 Follow-up of Discontinuations

Any subject who has been discontinued from the trial because of an AE considered to be related to the vaccine will be followed as deemed appropriate by the Investigator until resolution of the event or until chronicity of the event has been established. This will be documented in the source document and in the CRF.

5.3.6.5 Follow-up and Reporting of Pregnancies

Pregnancy is an exclusion criterion for enrolment in this trial, but subjects of child-bearing age could potentially become pregnant during their period of participation. Although pregnancy is a normal state, pregnancy occurring during the trial is to be recorded as an AE (but not as a SAE). Any complications during pregnancy (e.g. gestational diabetes or eclampsia) are also to be considered as AEs, however, these complications could result in the event being a SAE.

The Investigator is required to complete Part 1 of the "Initial Pregnancy Report Form" as soon as he/she is aware that the subject is pregnant. Part 1 contains information that includes the date of the last menstrual period, expected date of delivery, and the dates of vaccination. Part 2 contains follow-up information about delivery and newborn or pregnancy termination and is required to be completed at a follow-up after the due date.

[a] Voluntary withdrawal is also referred to as "withdrawal of consent" or "drop-out"

5.3.7 Medical History

Significant medical history (past and ongoing conditions, including influenza-like illness and vaccination history) will be documented in the CRF. For each condition, the following data will be collected:

- Diagnosis.
- Presence of the condition at enrollment.

The reporting of diagnosis (rather than signs or symptoms) will be encouraged.

Routine health care visits for pre-existing conditions, routine check-ups, medication prescription renewals, and stable pre-existing conditions as well as planned hospitalizations for elective surgery will not be recorded as AEs. Worsening of any documented pre-existing condition will become a reportable AE.

5.4 Modification of the Trial and Protocol

No amendments to this trial plan and protocol will be made without consultation with, and agreement of, the Sponsor. Any amendment to the trial that appears necessary during the course of the trial must be discussed by the Investigator and Sponsor concurrently. If agreement is reached concerning the need for an amendment, it will be produced in writing by the Sponsor and/or the Investigator and will become a formal part of the protocol. An amendment requires IEC/IRB approval. All amendments must also be forwarded to any applicable Regulatory Authorities.

An administrative change to the protocol is one that modifies administrative and logistical aspects of a protocol but does not affect the subjects' safety, the objectives or the progress of the trial. An administrative change does not require IEC/IRB approval. However, the IEC/IRB and the relevant Health Authorities must be notified whenever an administrative change is made.

The Investigator is responsible for ensuring that changes to an approved trial, during the period for which IEC/IRB approval has already been given, is not initiated without IEC/IRB review and approval except to eliminate apparent immediate hazards to the subject.

5.5 Interruption of the Trial

The trial may be discontinued for administrative reasons or if new data about the investigational product resulting from this or any other trials become available, and/or on advice of the Sponsor, the Investigators, and/or the IEC/IRBs. If a trial is prematurely terminated or suspended, the Sponsor shall promptly inform the Investigators, the Regulatory Authorities and the IEC/IRBs of the reason for termination or suspension.

If the trial is prematurely terminated for any reason, the Investigator should promptly inform the trial subjects and should assure appropriate therapy and follow-up for subjects.

6 Treatments

6.1 Vaccines Administered

Each subject will receive two doses of vaccine in the primary vaccination series (D0 [V01] and D21 [V03]) and a booster vaccination at 6 or 12 months after the first vaccination (see Trial flow-chart [see Figure 1 and Figure 2]).

6.2 Identity of Investigational Product(s)

6.2.1 Composition

The vaccines for primo vaccination (7.5 µg HA without adjuvant/0.6 mL or 30 µg HA with aluminum hydroxide as adjuvant [600 µg aluminum]/0.5 mL) will be presented in ready-to-use multidose vials.

All vaccines contain 45 µg of Thimerosal per dose.

6.2.2 Preparation and Administration

Preparation

Before injection, the products will be placed at room temperature for 5 to 10 minutes and must be gently shaken before use to obtain homogeneous suspensions. Volumes to be injected to the subjects according to the vaccine used are specified in Table 6.1.

The product should be withdrawn from the multidose vial with a sterile syringe, and the needle must be changed before injection.

Table 6.1: Volume to be Injected to Subjects According to Dosage

	**Formulations**			
	**30µg HA + Ad/0.5mL**		**7.5µg HA/0.6mL**	
**Volume injected per vaccination**	0.5 mL	0.25 mL	0.6 mL	0.3 mL
**Quantity of HA injected**	30µg	15µg	7.5µg	3.75µg

Note: Ad = aluminum hydroxide adjuvant

Administration

The vaccines will be injected intramuscularly into the deltoid region (for subjects aged 1 to 17 years; it is recommended to use the opposite side to that of the blood sampling) or thigh (for subjects aged less than 1 year) using a sterile syringe.

The IM injection is made at the center of the deltoid muscle between the shoulder and axilla and between the back and front of the arm (for subjects aged 1 to 17 years), or into the anterolateral aspect of the thigh (for subjects aged less than 1 year). Before injection, the syringe plunger will be drawn back in order to ensure that the injection is not administered intravascularly.

The site and side of injection will be recorded in the source document and in the CRF.

6.2.3 Precautions for Use

Vaccination must not be performed in subjects allergic to one of the constituents of the vaccine (particularly egg and chicken proteins).

As after any vaccination, subjects must be kept under observation for 30 minutes after each injection to ensure their safety. Appropriate equipment (e.g. adrenaline, corticosteroids) must be available on site in case of immediate allergic reactions.

All study products should be inspected visually for cracks, broken seals, correct label content (see Section 6.5.1) and extraneous particulate matter and/or discoloration prior to administration whenever solution and container permit. If any of these conditions exists, the vaccine should not be administered and a replacement dose may be used.

6.2.4 Dose Selection and Timing

The selection of formulation, dose and schedule of administration are based on the results of GPA01 (16), as described in Section 1.3 and Section 5.2.

6.3 Identity of Control Product

Not applicable.

6.4 Identity of Other Product

Not applicable.

6.5 Product Logistics

6.5.1 Labeling and Packaging

Multidose vials will be presented in an individual box. The vial and the box will bear the following information: study name, product name and dosage, storage temperature, injection route, Sponsor’s name, expiry date and legal information. Additional detachable labels bearing the same information will be provided. One label will be affixed to the source document and CRF of each subject vaccinated with the vial.

6.5.2 Storage and Shipment Conditions

Storage:

Products must be kept in a secure place with restricted access. Vaccines will be stored at a temperature ranging from +2°C to +8°C (in a refrigerator). The temperature must be monitored and documented on the appropriate form (see the Operating Guidelines) during the entire duration of the trial. In the event of accidental deep freezing or disruption of the cold chain, vaccines must not be administered; and the Investigator or the responsible person should contact the Clinical Research Associate (CRA) for further instructions.

Shipment:

The CRA will determine with the Investigator or the designee in charge of product management, the dates and times of delivery of products and forward the information to the Sponsor's Packaging Department for Clinical Trial Lots. Products will then be shipped to the center according to the predetermined schedule.

The person in charge of the Packaging Department for Clinical Trial Lots will issue a dispatch note with acknowledgement of receipt attached to the package.

On delivery of the products to the site, the Investigator or designee in charge of product management will check that the cold chain was maintained during shipment (verification of TESTO temperature recorder, freeze watch, and cold chain monitoring card). In the event of a problem, he/she should alert the CRA immediately.

The acknowledgement of receipt will be signed and dated by the person in charge of product management and will be sent according to the instructions given in the Operating Guidelines, together with the TESTO recorder.

6.5.3 Product Accountability

The Investigator will be personally responsible for product management or will designate a person who will be responsible for product management.

The Sponsor's monitoring staff will verify each trial site's product accountability records versus the record of administered doses in the CRFs.

The Investigator or the designee in charge of product management will maintain records of product delivery to the trial site, product inventory at the site, doses given to each subject, and the return of unused doses to the Sponsor.

The Investigator should alert the Sponsor as soon as possible of any expected or potential shortage of product doses during the trial so that the Sponsor can organize the shipment of extra doses.

6.5.4 Replacement Doses

Not applicable.

6.5.5 Return of Unused Products

Unused products will be returned to the Sponsor at the end of the vaccination period together with the form "Return Slip of Investigational Products from the Investigator Site", in accordance with the CRA's instructions. Empty boxes and partially used products will be destroyed on site after monitoring by the CRA. The destruction will be documented on site.

6.6 Randomization/Allocation Procedures

At V01 (D0), after verification of inclusion and exclusion criteria, each subject will be assigned an inclusion number consisting of 8 digits. The first 3 digits will correspond to the center number, and the last 5 digits will correspond to the chronological order of enrolment **within each age group** (9 to 17 years, 3 to 8 years and 6 to 35 months); the two groups of digits will be separated by a hyphen.

For example, the inclusion number of the first subject in Center 1, in the 9 to 17 year age group will be 001-00001. For subjects in the 3 to 8 year age group, the order of enrolment will commence at 100 and for subjects in the 6 to 35 month age group the order of enrolment will commence at 200, and so, for example, the inclusion number of the first subject at Center 2 in the 6 to 35 month age group will be 002-00201.

A randomization list will be prepared by the Sponsor's Biostatistics platform, for the two vaccine formulations.

The list will be created using the block permutation method. This guarantees, at any time, a similar number of subjects between the two vaccine formulation groups in each center.

One scratchable randomization list will be provided for each age group and each center.

Once assigned, the subject number cannot be reassigned to another subject, even if the subject discontinues the trial before vaccination.

If necessary to randomize the booster vaccination, a second randomization will be prepared at that time and the protocol will be amended appropriately.

6.7 Blinding and Code Breaking Procedures

Not applicable.

6.8 Concomitant Therapy

Ongoing medication, i.e. medication started before enrolment and continuing at the time of enrolment, will not be recorded in the CRF at V01.

At V01, V02, V03, V04, V05, and at V07 and V08 or V08 and V09, the following two categories of medication (non-allowed therapies and antipyretics/analgesics/ NSAIDs) taken since V01 and the previous visit will be recorded in the CRF, in the concomitant medication module.

- Non-allowed therapies:
  - Immunosuppressive therapy such as anti-cancer chemotherapy or radiation therapy or long-term systemic corticosteroid therapy.
  - Blood or blood derived products.
  - Vaccination other than with the trial vaccine.
- Antipyretics/analgesics/ NSAIDs (considered as a single category).

For each reported medication, the following will be documented:

- Trade name.
- Medication category (one of the above categories will be ticked).
- Given as treatment or as prophylaxis.
- Start and stop dates.

Dosage, indication, and administration route will not be recorded in the CRF. Homeopathic medication will not be recorded in the CRF.

Medication given in response to an AE will be captured in the "Action Taken" column of the AE. No details will be recorded in the concomitant medication module of the CRF unless the medication received belongs to one of the two prelisted categories.

In the event of a SAE, all therapies taken should be reported in the SAE reporting form, including the dosage, indication and route of administration information.

All therapies taken will be collected in the source document.

6.9 Treatment Compliance

The following measures will ensure that the treatment administered complies with the treatments planned, or that any non-compliance is documented so that it can be accounted for in the data analyses. All treatments will be administered by trial personnel. The Investigator or the person in charge of product management will maintain records of product delivery to the trial site, product inventory at the site, dose given to each subject, and the return of unused doses to the Sponsor. Detachable labels will be affixed in the source document, in the CRF and on the product dispensing list if needed after administration of each dose of vaccine. If not possible, the dose number must be written by hand in the space provided for the label. The injection group will be recorded in the CRF.

7 Specimens and Clinical Supplies

See Section 5.1 and the trial flow-chart (see Figure 1 and Figure 2) for details of the sampling schedule.

Subjects will have the opportunity to authorize the use of their blood samples for tests related to this study but not for future use for any other research.

7.1 Management of Samples for Humoral Immune Response

7.1.1 Sample Collection for Serological Assessments

For serology assessments, 7 mL (Groups 1 and 2) or 2 mL (Group 3) of blood will be collected in dry tubes at each visit (except for the 8-day safety follow-up visits [V02 and V04] and the 6-month follow up visit/telephone call).

If blood is not drawn by the Investigator, then immediately prior to drawing blood, the person in charge of the procedure will verify the subject's identity. Each tube of blood will be clearly labeled with the subject identification number and the sampling stage using a self-adhesive label that will be stuck onto the tube immediately before blood sampling.

7.1.2 Sera Preparation of Blood Samples for Serological Assays

Sample preparation procedures will be described in detail in the Operating Guideline instruction manual provided to the Investigator's team.

The sampling tube should be stored at room temperature for a minimum of 1 hour and a maximum of 2 hours after sampling and before centrifugation. The tube must be stored vertically and must not be shaken.

Beyond 2 hours, the sampling tube is to be placed at a temperature between 2°C and 8°C and must be centrifuged within a maximum of 24 hours.

After being allowed to clot at room temperature, blood samples will be centrifuged before being divided into appropriate aliquots of serum. Samples will then be handled one subject at a time to avoid the mix-up of the subjects' blood tubes. Serum will be transferred to the appropriate number of tubes after the tubes have been labeled with self-adhesive labels that clearly identify the subject inclusion number and sampling stage or visit number.

The subject inclusion number, the date of sampling, and the number of primary tubes and retention tubes obtained will be specified on a sample identification list. Comments may be made on the quality of samples in the space provided on this list.

Serum aliquots will be frozen immediately at ≤-17°C until testing.

7.1.3 Sera Storage and Shipment of Samples for Serological Assays

Serum tubes will be stored frozen at ≤-17°C and shipped frozen to the appropriate laboratory. The storage temperature will be monitored and documented on the appropriate form (the operating guidelines will provide further details) during the entire trial.

Testing will be performed in the following laboratories:

- For HI (with turkey erythrocytes) – Groups 1 and 2 only: J. WOOD, National Institute for Biological Standards and Control (NIBSC), Blanche Lane, South Mimms, Potters Bar, Hertfordshire EN6 3QG, UK.
- For HI (with horse erythrocytes) and SN: Global Clinical Immunology (GCI) laboratory, Swiftwater, Pennsylvania, USA.

7.1.4 Retention of Unused Serum Samples for Repository (Humoral)

For each blood sample drawn for serum Ab response assessment, a maximum of 3 serum aliquots of 1 mL will be obtained. Of these aliquots:

- one will be used for HI (with turkey erythrocytes) testing (J. Wood's laboratory)
- one will be used for HI (with horse erythrocytes) and SN (GCI)
- the remaining serum will be kept in the serum bank at the Sponsor's laboratory in Swiftwater, Pennsylvania, USA at least 10 years after the vaccine being tested has been approved for use by the health authorities. The Investigator will ask for IEC approval in case of storage of blood sample beyond 5 years. This additional serum sample may be used in case of insufficient volume of serum in the tubes planned for the analyses. If not used for replacement, these stored samples may be used for other evaluation studies, e.g. studies of the immune response to influenza vaccines, improvement of knowledge and documentation of the safety of this vaccine by using new developed laboratory methods or applying newly discovered concepts. In such cases, the Investigator will ask for IEC approval before implementation of these evaluation studies and samples will be anonymized. No genetic studies will be performed. Subjects will have the choice in the ICF to accept or refuse the future use of their samples.

Procedure of sample destruction

Blood sample will be autoclaved and then incinerated.

7.2 Management of Samples for Cellular Mediated Immune Response

7.2.1 Sample Collection for Cellular Mediated Immune Response Assessments

For CMI assessments, 2 mL of blood will be collected in sodium heparin tubes from Group 3 only (120 subjects in total) at V01 and V04.

If blood is not drawn by the Investigator, then immediately prior to drawing blood, the person in charge of the procedure will verify the subject's identity. Each tube of blood will be clearly labeled with the subject identification number and the sampling stage using a self-adhesive label that will be stuck onto the tube immediately before blood sampling.

7.2.2 Cellular Mediated Immune Response Assessment

Sample processing will be described in detail in the Operating Guideline instruction manual provided to the Investigator's team.

The sampling tube should be stored at room temperature and gently agitated for a maximum of 4 hours before processing.

Blood stimulation will be performed according to a standard (non-validated) protocol as follows:

Blood (200 µL/well) from a single sample will be aliquoted into 8 wells in a 96 well plate. Flu or control antigens (50 µL) will be added to the blood. Twelve blood samples will be processed in

one plate. The plate will be incubated 4 days at 37°C, 5% CO_2 and then centrifuged for 10 minutes at 400g. Cell supernatant (200 µL) will be collected and aliquoted into two new plates labeled in advance (2 x 100 µL) following the same original plate layout. The plates will be sealed and stored at -80°C until shipment.

7.2.3 Storage and Shipment of Samples for Cellular Mediated Immune Response Assessment

For each blood sample for CMI assessment two plates will be obtained. Each plate will contain 12 blood samples. Plates will be kept at -80°C in a freezer. They will be transferred to the Sponsor on dry ice, at the following address:

Nolwenn Nougarède/Frederique Jantet
sanofi pasteur, Batiment X Nord
1541, Avenue Marcel Mérieux
69280 Marcy l'Etoile
France

The shipment will be organized in accordance with the requirements applicable for the air transport of infectious substances (International Air Transport Association 6.2 regulations)

7.3 Clinical Supplies

The Sponsor will provide the protocol, ICFs, assent forms, CRFs, DCs, MAs, Operating Guidelines, and trial material such as temperature recorder TESTO (which will be retrieved at the end of the trial), rulers, thermometers, and syringes/needles for vaccine administration. Either the Sponsor or the Investigator will provide the blood drawing material (except for that used for taking the samples for CMI analysis).

8 Assessments Methods and Endpoints

8.1 Immunogenicity Endpoints and Assessments Methods

8.1.1 Endpoints

Immunogenicity

Anti- HA Ab titers against the A/H5N1 strain will be expressed as described below (the assay will be performed using horse erythrocytes for all subjects and using turkey erythrocytes for subjects in Group 1 and Group 2; anti-HA titers will not be measured using turkey erythrocytes for Group 3 due to the extra blood sample volume that would be required):

- HI titer obtained in duplicate on D0, D21, and D42, and summarized at the subject level by individual GMs of duplicates at each timepoint. The following endpoints will be derived:
    - Individual titer ratios D21/D0, D42/D0, and D42/D21.

    - Proportion of subjects with a titer ≥40 (turkey) or ≥32 (horse) 1/dilution (dil) on D0, D21 and D42.
    - Seroconversion (for subjects with a titer <10 [turkey] or <8 [horse] [1/dil] on D0: post-injection titer ≥40 [turkey] or ≥32 [horse] [1/dil])

    or

    - Significant increase (for subjects with a titer ≥10 [turkey] or ≥8 [horse] [1/dil]: ≥four-fold increase of the titer) on D21 and D42.
- SN titer obtained in duplicate on D0, D21, and D42 and summarized at the subject level by individual GMs of duplicates at each timepoint. The following endpoints will be derived:
    - Individual titer ratios D21/D0, D42/D0, and D42/D21.
    - Two- and four-fold increase from D0 to D21 and to D42.

Antibody persistence

Anti-HA titer (HI method) and neutralizing Ab titer (SN method) in duplicate prior to the booster vaccination at M6 (D180) or M12 (D365).

Booster vaccination response

- HI titer obtained in duplicate at V07 (M6) (for 6-month booster design) or V08 (M12) (for 12-month booster design), V08 (M6+21 days) (for 6-month booster design) or V09 (M12+21 days) (for 12-month booster design), and summarized at the subject level by individual GMs of duplicates at each timepoint. The following endpoints will be derived:
    - Individual titer ratios V08/V07 (for 6-month booster design) or V09/V08 (for 12-month booster design).
    - Proportion of subjects with a titer ≥40 (turkey) or ≥32 (horse) (1/dil) at V07 and V08 (for 6-month booster design) or V08 and V09 (for 6-month booster design).
    - Seroconversion (for subjects with a titer <10 [1/dil] at V07 (for 6-month booster design) or V08 (for 12-month booster design): post-injection titer ≥40 [turkey] or ≥32 [horse] [1/dil]) at V08 (for 6-month booster design) or V09 (for 12-month booster design)

    or

    - Significant increase (for subjects with a titer ≥10 [1/dil]: ≥four-fold increase of the titer) at V08 (for 6-month booster design) or V09 (for 12-month booster design).
- SN titer obtained in duplicate at V07 and V08 (for 6-month booster design) or V08 and V09 (for 12-month booster design) and summarized at the subject level by individual GMs of the duplicates at each timepoint. The following endpoints will be derived:
    - Individual titer ratios V08/V07 (for 6-month booster design) or V09/V08 (for 12-month booster design).
    - Two- and four-fold increase from V07 to V08 (for 6-month booster design) or V08 to V09 (for 12-month booster design).

8.1.1.1 Observational Endpoints

Secretion of a panel of Th1 (interferon-gamma [IFN-γ], tumor necrosis factor-alpha [TNF-α], interleukin-2 [IL-2]), and Th2 (IL-5, IL-4, IL-10, IL-13) cytokines by peripheral blood mononuclear cells (PBMCs), upon in vitro re-stimulation with vaccine antigens will be quantified before the first vaccination and 8 days after the second vaccination.

8.1.2 Assessment Methods

8.1.2.1 Hemagglutination Inhibition Test

The HI test adapted to the avian strain will be performed. The principle of the HI test is based on the ability of specific anti-influenza antibodies to inhibit hemagglutination of horse or turkey red blood cells (RBCs) by influenza virus HA. The sera to be tested have to be previously treated to eliminate the non-specific inhibitors and the anti-species HAs.

Serum Pre-treatment

- Elimination of non-specific inhibitors by incubation of the unknown serum samples and quality control sera (serum of ferret or human immunized with influenza virus) with neuraminidase (18 hours at +36°C to +38°C in an incubator and 1 hour at +56°C in a water-bath).
- Absorption of spontaneous anti-species agglutinins by incubation of the serum samples and quality control sera from which non-specific inhibitors have been already removed, with the RBC suspension (2 hours at +2°C to +8°C). After these steps, the mixtures are centrifuged (10 minutes at 700g) and the supernatants are submitted to the HI method.

Preparation will be performed simultaneously for serum obtained on D0, D21, and D42.

HI Test

For HI [turkey] 12 two-fold dilutions of the 1:10 dilution of serum sample (or quality control sera) are performed and incubated with the HA antigen suspension (previously titrated to adjust the dilution at 4 HAU [HA units]/25 µL). The HA antigen is not added to the well dedicated to each serum quality control. For HI [horse] the starting dilution is 1:8.

The mixture is incubated for 1 hour at room temperature and 25 µL of the 0.4% RBC suspension are added. The reaction is left for 1 hour at room temperature before reading.

Reading

The serum titer is equal to the highest reciprocal dilution, which induces a complete inhibition of hemagglutination. The titer of each quality control serum is close to the previously assigned value (within one serial two-fold dilution limits).

The RBC control (RBC suspension without antigen) and the serum control (for each sample tested) do not produce any agglutination.

Each serum sample is titrated in duplicate. The final titer is equal to the GM of the two results, which should not differ by more than a two-fold serial dilution.

8.1.2.2 Seroneutralization

The influenza virus microneutralization test is a specific assay for antibodies to the avian influenza A (H5N1) virus in human serum and could potentially be used to detect antibodies to other avian subtypes. This microneutralization test is more sensitive than the HI assay (the traditional assay for antibodies to human influenza A and B viruses), as this assay can detect H5-specific Ab in human serum at titers that can not be detected by the HI assay.

Inactivated human serum samples are pre-inoculated with a standardized amount of virus prior to the addition of Madin-Darby canine kidney (MDCK) cells. After overnight incubation, Enzyme Linked Immunosorbent Assay (ELISA) is used to measure the viral NP protein in infected MDCK cells. Since serum Ab to the influenza virus HA inhibit the viral infection of MDCK cells, the optical density results of the ELISA are inversely proportional to the serum Ab concentration.

8.1.2.3 Cellular Mediated Immune Response

The CMI will be performed by sanofi pasteur Research Department, Marcy l'Etoile, France. The technology used is under development and therefore is not validated.

Cell supernatants from V01 and V04 for each subject will be thawed and tested for cytokines (IL-2, IL-4, IL-5, IL-10, IL-13, IFN-γ, and TNF-α) content using standard commercially available kits based on Luminex technology, according to the manufacturer's protocol. Results will be expressed in pg/mL.

8.1.3 Handling of Missing Data and Outliers

Missing or incomplete data will not be replaced, with the exception of the following:

- All values strictly under the lower limit of quantification (LLOQ) will be treated as LLOQ/2.
- All values above or equal to the upper limit of quantification (ULOQ) will be truncated as ULOQ.

These above replacements of data may have an impact on most parameters.

No search for outliers will be done.

8.2 Efficacy Endpoints and Assessments Methods

No efficacy data will be obtained in the trial.

8.3 Safety Endpoints and Assessments Methods

8.3.1 Definitions

The following definitions are extracted from the ICH E2A Guideline for Clinical Safety Data Management: Definitions and Standards for Expedited Reporting.

Adverse event:

An AE is any untoward medical occurrence in a patient or clinical investigation subject administered a pharmaceutical product and which does not necessarily have to have a causal relationship with this treatment. An AE can therefore be any unfavorable and unintended sign (including an abnormal laboratory finding, for example), symptom or disease temporally associated with the use of a medicinal product, whether or not considered related to the medicinal product.

The worsening of an existing sign of symptom is also considered as an AE. Therefore an AE may be:

- A new illness.
- The worsening of a concomitant illness.
- An effect of vaccination, including the comparator.
- A combination of the above.

Surgical procedures are not AEs; they are the action taken to treat a medical condition. It is the condition leading to the action taken that is the AE (if it occurs during the trial period).

Medical conditions leading to surgery that started prior to the trial but did not worsen during the trial are not to be reported as AEs.

Serious adverse event:

Serious and *severe* are not synonymous. The term *severe* is often used to describe the intensity (severity) of a specific event (as in mild, moderate, or severe myocardial infarction); the event itself, however, may be of relatively minor medical significance (such as severe headache). This is not the same as *serious* which is based on patient/event outcome or action criteria usually associated with events that pose a threat to a patient's life or functioning. Seriousness, not severity, serves as a guide for defining regulatory reporting obligations.

An SAE is any untoward medical occurrence that at any dose (including overdose):

- Results in death.
- Is life-threatening[a].
- Requires inpatient hospitalization or prolongation of existing hospitalization[b].
- Results in persistent or significant disability/incapacity[c].
- Is a congenital anomaly/birth defect.

---

a *The term "life-threatening" refers to an event in which the patient was at risk of death at the time of the event; it does not refer to an event which hypothetically might have cause death if it were more severe.*

b *All medical events leading to hospitalizations will be recorded and reported as Serious Adverse Events, with the exception of: hospitalization planned before inclusion into the trial or out-patient hospitalization with no overnight hospitalization*

c *"Persistent or significant disability or incapacity" means that there is a substantial disruption of a person's ability to carry our normal life functions*

- Is an important medical event[a].

Adverse Reaction (AR):

All noxious and unintended responses to a medicinal product related to any dose should be considered adverse drug reactions.

(The phrase "responses to a medicinal product" means that a causal relationship between a medicinal product and an AE is at least a reasonable possibility).

Unexpected adverse drug reaction (ADR):

An adverse reaction, the nature or severity of which is not consistent with the applicable product information (e.g. Investigator's Brochure for an unapproved investigational medicinal product).

The following additional definitions are used by the Sponsor:

Solicited Reaction

A solicited reaction is a **term prelisted in the CRF.** The assessment of these events post-vaccination is mandatory. A solicited event is defined by a combination of:

- Symptom.
- Onset post-vaccination.

e.g., injection site pain between D0 and D3 post-vaccination, or headache between D0 and D7.

A solicited reaction is therefore an AR observed and reported under the conditions (nature and onset) prelisted (i.e., solicited) in the CRF.

Unsolicited AE / ADR

An unsolicited AE is an observed AE that does not fulfill the conditions prelisted in the CRF in terms of symptom and/or onset post-vaccination, e.g., if headache between day 0 and day 7 is a solicited event (i.e., prelisted in the CRF), then a headache starting on day 7 is a solicited event, whereas headache starting on day 8 post-vaccination is an unsolicited event.

Injection Site Reaction

An injection site reaction[b] is an ADR of and around the injection site. Injection site reactions are commonly inflammatory reactions.

---

[a] *Medical and scientific judgment should be exercised in deciding whether expedited reporting is appropriate in other situations, such as important medical events that may not be immediately life-threatening or result in death or hospitalization but may jeopardize the patient or may require intervention to prevent one of the other outcomes listed in the definition above. These should also usually be considered serious. Examples of such events include allergic bronchospasm requiring intensive treatment in an emergency room or at home, blood dyscrasias or convulsions that do not result in inpatient hospitalization, or the development of drug dependency or drug abuse, new onset diabetes or autoimmune disease.*

[b] All injection site AEs are considered to be related to vaccination and are therefore all *injection site reactions.*

Systemic AE

Systemic AEs are all AEs that are not injection site reactions. They therefore include systemic manifestations such as headache, fever, as well as local or topical manifestations that are not associated with the vaccination site, e.g., rash that is localized but that is not at the injection site.

8.3.2 Endpoints

- Occurrence, nature, (MedDRA preferred term), duration, severity, and relationship to vaccination for any unsolicited systemic adverse events reported in the 30 minutes after each injection.
- The occurrence, time to onset, number of days of occurrence, severity, and seriousness of solicited (prelisted in the subject diary and CRF) injection site reactions and systemic reactions occurring within 7 days following each injection will be reported (including the booster injection).
- The occurrence, nature (MedDRA preferred term), severity, relationship to vaccination and seriousness of unsolicited (spontaneously reported) AEs within 21 days following each injection will be reported (including the booster injection).
- The occurrence, nature, time to onset, and relationship to vaccination of serious adverse events (SAEs) during the whole study period will be reported.
- The occurrence of the following reactions (MedDRA Preferred Terms given in parentheses) in the three days following each injection will be more especially reported (as defined by the CHMP Note for Guidance [CPMP/BWP/214/96]) (including the booster injection)[a]:
  - Injection site induration >5 cm observed for more than 3 days.
  - Injection site ecchymosis (injection site hemorrhage).
  - Rectal equivalent temperature >38°C for 24 hours or more (pyrexia).
  - Malaise.
  - Shivering (chills).

8.3.2.1 Exploratory Endpoints

8.3.3 Safety Assessment methods

8.3.3.1 30-Minute Observation Period

Subjects will be kept under observation for 30 minutes after each vaccination to ensure their safety. Any AE (unsolicited systemic AEs) occurring during this 30-minute period will be recorded in the AE tables of the CRF. If the event is among the list of solicited reactions, it should be reported in the solicited tables only, without specification of the time of onset. Should any SAE

[a] Note that it may be difficult to collect certain of these for Group 3 (e.g. malaise)

occur during this 30 minute period, then the SAE should be reported to the Sponsor according to the procedure described in Section 9.1.

8.3.3.2 Reactogenicity (Solicited Reactions from D0 to D7 after each Vaccination)

After vaccination, the subjects will be provided with a safety DC, a digital thermometer and a flexible centimeter ruler and will be instructed how to record the following items in the safety DC on the day of vaccination and daily for the next 7 days (i.e. D0 to D7).

- Maximum daily axillary temperature.
- Maximum daily measurement or maximum severity grade of all other solicited injection site or systemic reactions.
- Date of last day of presence of the reaction if it lasted beyond D7.
- Action taken for each event, if any (medication etc).

Tables 8.1, 8.2, 8.3 and 8.4 present the injection site and systemic reactions that will be pre-listed in the safety DCs and CRFs, together with the severity scales for subjects aged <2 years and subjects aged ≥2 years.

The D0 to D7 safety data following the two primary series vaccinations will be collected and reviewed at V02 and V04, 8 days after the vaccination. These data will be reviewed for Groups 1 and 2 by the Sponsor prior to progression to the next age group (Groups 2 and 3, respectively).

Table 8.1: Solicited Injection Site Reactions: Definitions, Terminology and Severity Scales for Subjects Aged 6 months to <2 years

	**Injection site tenderness**	**Injection site erythema**	**Injection site swelling**	**Injection site induration**
**Diary Card term***	Tenderness	Redness	Swelling	Hardening
**Definition**	See severity scale	Presence of a redness including the approximate point of needle entry	Swelling at or near the injection site<br>Swelling or edema is caused by a fluid infiltration in tissue or cavity and, depending on the space available for the fluid to disperse, swelling may be either soft (typically) or firm (less typical) to touch and thus can be best described by looking at the size of the swelling	Hardening at or near the injection site<br>Hardening is caused by a slow diffusion of the product in the tissue leading to a thick or hard area to touch at or near the injection site and thus can be best described by looking at the size of the hardening
**Severity scale†**	Mild: minor reaction when injection site is touched<br>Moderate: cries and protests when injection site is touched<br>Severe: cries when injected limb is moved or the movement of the injected limb is reduced	Mild: <2.5 cm<br>Moderate: ≥2.5 to <5<br>Severe ≥5 cm	Mild: <2.5 cm<br>Moderate: ≥2.5 to <5 cm<br>Severe: ≥5 cm	Mild: < 2.5 cm<br>Moderate: ≥2.5 to <5<br>Severe ≥5 cm

* These terms are the ones that will appear in the safety DC.

† For redness and swelling, this information is provided for information only. The classification as mild, moderate or severe will be done by the Sponsor's statistical department. The maximum daily measurement of the reaction will be reported in the safety DC and then in the CRF. The severity scale for the tenderness will be provided in the diary card and CRF for ease of reference.

Table 8.2: Solicited Injection Site Reactions: Definitions, Terminology and Severity Scales for Subjects Aged ≥2 years

	**Injection site pain**	**Injection site erythema**	**Injection site swelling**	**Injection Site Induration**	**Injection Site Ecchymosis**
**Diary Card term***	Pain	Redness	Swelling	Hardening	Bruising
**Definition**	See severity scale	Presence of a redness including the approximate point of needle entry	Swelling at or near the injection site<br>Swelling or oedema is caused by a fluid infiltration in tissue or cavity and, depending on the space available for the fluid to disperse, swelling may be either soft (typically) or firm (less typical) to touch and thus can be best described by looking at the size of the swelling	Hardening at or near the injection site<br>Hardening is caused by a slow diffusion of the product in the tissue leading to a thick or hard area to touch at or near the injection site and thus can be best described by looking at the size of the hardening	Ecchymosis is the result of the diffusion of blood in the skin from ruptured blood vessels that forms a purple or black and blue spot on the skin. It can be best described by looking at its size
**Severity scale†**	Mild = Easily tolerated<br>Moderate = Sufficiently discomforting to interfere with normal behavior or activities<br>Severe = Incapacitating, unable to perform usual activities, may have/or required medical care or absenteeism	Mild: < 2.5 cm<br>Moderate: ≥2.5 to <5 cm<br>Severe ≥5 cm	Mild: < 2.5 cm<br>Moderate: ≥2.5 to <5 cm<br>Severe ≥5 cm	Mild: < 2.5 cm<br>Moderate: ≥2.5 to <5 cm<br>Severe ≥5 cm	Mild: < 2.5 cm<br>Moderate: ≥2.5 to <5 cm<br>Severe ≥5 cm

* These terms are the ones that will appear in the safety DC

† For erythema, swelling, induration and ecchymosis, this information is provided for information only. The classification as mild moderate or severe will be done by the Sponsor's statistical department. The maximum daily measurement of the reaction will be reported in the safety DC and then in the CRF. The severity scale for the pain will be provided in the DC and CRF for ease of reference.

Table 8.3: Solicited Systemic Reactions: Definitions, Terminology and Severity Scales For Subjects Aged 6 months to <2 years

	**Fever**	**Vomiting**	**Crying abnormal**	**Drowsiness**	**Appetite lost**	**Irritability**
**Diary Card term***	Temperature (axillary)	Vomiting	Abnormal crying	Drowsiness	Loss of appetite	Irritability
**Definition**	Elevation of axillary temperature to ≥37.4°C	Vomiting does not include spitting-up.	Inconsolable crying without a reason.	Reduced interest in surroundings or increased sleeping.	See severity scale	An excessive response to stimuli: increased fussiness, whining, and fretfulness despite attempts to comfort the infant and despite parental responses that would normally be soothing.
**Severity scale†**	Mild: ≥37.4-37.9°C<br>Moderate: ≥38-38.9°C<br>Severe: ≥39°C	Mild: 1 episode per 24 hours<br>Moderate: 2-5 episodes per 24 h<br>Severe: ≥6 episodes per 24 h or requiring parenteral hydration	Mild: < 1 hour<br>Moderate: 1-3 h<br>Severe: >3 h	Mild: Sleepier than usual or less interested in surroundings<br>Moderate: not interested in surroundings or did not wake up for a feed/meal<br>Severe: Sleeping most of the time or difficulty to wake up	Mild: eating less than normal<br>Moderate: missed 1 or 2 feeds/meals completely,<br>Severe: refuses ≥3 feeds/meals or refuses most feeds/meals‡	Mild: easily consolable<br>Moderate: requiring increased attention<br>Severe: inconsolable

* These terms are the ones that will appear in the safety DC.

† For fever, this information is provided for information only. The classification as mild moderate or severe will be done by the Sponsor's statistical department. The maximum daily body temperature will be recorded in the diary and then in the CRF. Severity scales for the other reactions will be provided in the diary card and CRF for ease of reference.

‡ The same scale will be used for a 6-month baby and a child less than 2 years. The quantity of food is not the same, but the severity of loss of appetite is comparable.

Table 8.4: Solicited Systemic Reactions: Definitions, Terminology and Severity Scales For Subjects Aged ≥2 years

	Fever	Headache	Malaise	Myalgia	Shivering
**Diary Card term***	Temperature	Headache	Feeling unwell	Muscle aches and pains	Shivering
**Definition**	Fever is defined by an axillary temperature of ≥37.4°C	A headache is pain or discomfort in the head, or scalp. Does not include migraine.	General ill feeling<br>Malaise is a generalized feeling of discomfort, illness, or lack of well-being that can be associated with a disease state. It can be accompanied by a sensation of exhaustion or inadequate energy to accomplish usual activities	Muscle aches and pains are common and can involve more than one muscle at the same time. Muscle pain can also involve the soft tissues that surround muscles. These structures, which are often referred to as connective tissues, include ligaments, tendons, and fascia (thick bands of tendons).<br>Does not apply to muscle pain at the injection site which should be reported as injection site pain.	Cold feeling
**Severity scale†**	Mild<br>≥37.4°C – 37.9°C Oral<br>Moderate ≥38°C – 38.9°C Oral<br>Severe ≥39°C Oral	Mild<br>Noticeable but does not interfere with daily activities<br>Moderate<br>Interferes with daily activities<br>Severe<br>Prevents daily activities	Mild<br>Noticeable but does not interfere with daily activities<br>Moderate<br>Interferes with daily activities<br>Severe<br>Prevents daily activities	Mild<br>Noticeable but does not interfere with daily activities<br>Moderate<br>Interferes with daily activities<br>Severe<br>Prevents daily activities	Mild<br>Noticeable but does not interfere with daily activities<br>Moderate<br>Interferes with daily activities<br>Severe<br>Prevents daily activities

* These terms are the ones that will appear in the safety DC

† For fever, this information is provided for information only. The classification as mild moderate or severe will be done by the Sponsor's statistical department. The maximum daily body temperature will be recorded in the DC and then in the CRF. Severity scales for the other reactions will be provided in the DC and CRF for ease of reference.

Important notes for the accurate assessment of fever

Fever is defined, clinically, as a rectal temperature of 38.0°C or more. However for practical reasons, fever will be defined in this trial as axillary temperature of 37.4°C or more. Tympanic thermometry must not be used.

Whatever the route used, the Investigator will record in the CRF the temperature measured **without** applying any conversion factor. The CRF and the DC will provide the possibility to specify the temperature measurement route used in case it is not the requested route. Temperature should be measured once per day, at the same time each day preferably in the evening, and at the time of any apparent fever. The highest observed daily temperature should be recorded in the DC.

For the statistical analysis, results will be presented as rectal equivalent temperature. The following relationship will be used for the conversion:

Rectal temperature = Axillary temperature + 0.6°C

8.3.3.3 Adverse Events from D0 to D21 after each Vaccination

Subjects will also be instructed to record any other medical events that may occur between D0 and D21 after each vaccination. Space will be provided in the DC for this purpose. For each event, the following will be recorded:

- Start and stop dates.
- Severity of the event according to the following scale:

  Mild: Noticeable but does not interfere with daily activities.

  Moderate: Interferes with daily activities.

  Severe: Prevents daily activities.
- Action taken for each event, if any (medication etc).

AEs likely to be related to the product (or to the experiment[a]), whether serious or not, which persist at the end of the trial will be followed up by the Investigator until their complete disappearance or the stabilization of the subject's condition. The Investigator will inform the Sponsor of the date of final disappearance of the AE and will document it on a CRF data correction sheet.

8.3.3.4 Transcription of Safety Information to the CRF; Causality Assessment

At each visit, the Investigator will perform a physical examination and will interview the subject for any solicited reactions and unsolicited AEs recorded in the DC, as well as regarding any other AEs that may have occurred. All relevant data will be transcribed into the CRF according to the instructions provided with the CRF.

The ***action taken*** to treat a solicited reaction or any AEs will be classified in the CRF using the following scale:

---

[a] European Directive 2001/21/EC, for trials conducted in Europe only

0 = No action.

1 = Medication[a].

2 = Health care contact (defined as: physician/nurse telephone contact; physician/nurse evaluation; emergency visit/outpatient hospitalization or tel. contact).

3 = Health care contact and prescription of a new medication.

4 = Hospitalization (inpatient).

The Investigator will assess the ***causal relationship*** between each unsolicited AE[b] and vaccination as either not related or related, based on the following definitions:

0 Not related / No relationship: The AE is clearly/most probably caused by other etiologies such as subject's underlying condition, therapeutic intervention or concomitant therapy, or the delay between vaccination and the onset of the AE is incompatible with a causal relation, or the AE onset is before vaccination.

1 Related*:* There is a reasonable possibility that the AE was caused by the vaccine. The expression "reasonable possibility" is meant to convey in general that there are facts (evidence) or arguments to suggest a causal relationship (ICH Guidelines. Clinical Safety Data Management E2A).

Serious Adverse Events (see Section 9.1 for information on SAE reporting)

Information on SAEs will be collected and assessed throughout the trial.

The SAEs will be reported by the Investigator using SAE Reporting Forms (the procedure for reporting SAEs is described in Section 9.1). Any relevant information concerning the SAE is to be reported on this form, and if relevant information became available later, the form that was sent initially must be updated (e.g. outcome, medical history, results of investigations, copy of hospitalization reports). The Investigator will assess the causal relationship between the SAE and the vaccine with the scale provided above.

8.3.4 Handling of Missing Data and Outliers

Missing safety data will not be replaced. All vaccinated subjects with safety data, and all safety data recorded in the CRFs will be included in the safety analyses. No search for outliers will be performed.

---

[a] Self-medication, such as over the counter medication.

[b] By convention, all solicited events will be considered as related to vaccination and referred to as reactions. For these reactions, the investigator's opinion will not be requested in the CRF.

9 Serious Adverse Events

9.1 Reporting of Serious Adverse Events

In order to comply with current regulations on SAE reporting to Health Authorities and to allow the Sponsor to conduct a detailed analysis of the safety of the developed products, the Investigator will accurately document any SAE, in accordance with the notification deadlines stated below, to provide the Sponsor with all necessary information and, if requested by the Sponsor, to give access to source documents.

9.1.1 Initial Reporting by the Investigator

The Investigator must report every SAE occurring during a subject's participation in the trial to the Sponsor's Global Pharmacovigilance Department (GPVD) within 24 hours of finding out about the SAE. The SAE must be reported even if the Investigator considers that the SAE is not related to the vaccine.

The Investigator must complete the "SAE Reporting Form" and send it by facsimile to the Sponsor's Pharmacovigilance department at the following number:

Fax: +33 4 37 37 71 32

If no fax machine is available, the notification of the SAE should be made by telephone to the Local Medical Manager. The Investigator will then send the completed 'SAE Reporting Form' by express mail to the following address:

Global Pharmacovigilance Department
Sanofi Pasteur SA
2, avenue Pont Pasteur
69367 Lyon cedex 07
France

9.1.2 Follow-up Reporting by the Investigator

If further relevant information concerning the SAE becomes available later (e.g., outcome, precise description of medical history, results of the investigation, copy of hospitalization report), the SAE Reporting Form sent initially must be completed and the box "Follow-up Reporting Form" must be ticked on the form. It must be reported to the Sponsor's Global Pharmacovigilance Department within 24 hours after the Investigator becomes aware of the new information. The SAE Reporting Form will be sent by fax or express mail as described above for the Initial Reporting.

The anonymity of the subjects must always be respected when forwarding this information.

9.1.3 Reporting of SAEs Occurring After Subject Trial Termination

Any SAE occurring after subject trial termination but likely to be related to the product must also be reported by the Investigator as soon as he/she is alerted of it. In such a case, the procedure to be followed to report the SAE to the Sponsor is identical to that described above.

9.1.4 Causal relationship

The causal relationship between the SAE and the product (yes or no) will first be evaluated by the Investigator using the following definitions:

0 Not related / No relationship: The AE is clearly/most probably caused by other etiologies such as patient’s underlying condition, therapeutic intervention or concomitant therapy, or the delay between vaccination and the onset of the AE is incompatible with a causal relation, or the AE onset before vaccination.

***1 Related**:* There is a reasonable possibility that the AE was caused by the vaccine.
The expression “reasonable possibility” is meant to convey in general that there are facts (evidence) or arguments to suggest a causal relationship (ICH Guidelines. Clinical Safety Data Management E2A).

Then, according to the available information and the current medical knowledge, the Sponsor's Product Safety Officer will also assess the causal relationship to the product in collaboration with the Clinical Team Leader (CTL).

The decision to modify or discontinue the trial, or to break individual or all trial codes may be made after mutual agreement between the Sponsor and the Investigator(s).

9.1.5 Reporting SAEs to Health Authorities and IECs/IRBs

The Sponsor will inform health authorities of any reportable SAE according to the local regulatory requirements. Reporting to the Health Authorities will be according to the Sponsor's standard operating procedures.

The Sponsor's Responsible Medical Officer (the CTL) will notify the Investigators in writing of the occurrence of a reportable SAE. The Investigators will be responsible for informing the IECs or IRBs that reviewed the trial protocol.

9.1.6 Additional Information

If seizure occurs after vaccination, this will be considered as an SAE. The subject will not receive further vaccinations irrespective of his/her age if the seizure is assessed as related to the vaccination. However the subject will be monitored and followed-up for safety information.

If it can help to analyze the SAE, a blood sample (5 mL in a dry tube) can be taken.

For reported deaths, the Investigator should supply the Sponsor and the IEC/IRB with any additional requested information such as autopsy reports or terminal medical reports.

10 Data Collection and Management

10.1 Data Collection, CRF Completion

All clinical trial information will be reported by the Investigator or a designated person on a CRF designed specifically for this trial and provided by the Sponsor. The Sponsor will provide all necessary tools and instructions to complete the CRF. All CRFs must be signed by the Investigator. The Sponsor requires explanations for all missing information. Incorrect data must be crossed-out with a single line, then initialed and dated. Correction fluid or similar correction methods that mask the original data are not to be used. These rules also apply to the completion of SAE Reporting Forms, Data Correction Forms, and Complementary Information Forms.

To ensure the correct and consistent completion of the CRFs and the respect of the visit procedures, the CRF will contain the following information:

- All necessary scales for the rating of severity and causality of events (these scales are presented in Section 8.3.3).
- The trial flow-chart.
- A list of specific procedures to be followed at each visit.

In addition, detailed guidance for the completion of the CRF will be provided in a separate document.

Standard individual safety diaries, specifically designed for this trial and provided by the Sponsor, will be used by the subjects to record daily safety information between D0 and D21 after each vaccination. These diaries will include prelisted terms (see Section 8.3) and areas for free text. Subjects will be provided with diaries, rulers (for measuring the size of injection site reactions) and standard digital thermometers, and will be instructed how to use them. Severity or intensity scales will be provided in the diaries.

At each visit, the Investigator or designated person will transcribe the appropriate post-vaccination safety information (for the period since the previous visit) from the DC to the CRFs after interviewing the subject to ensure that the information is complete and correct.

10.2 Data Management

Data generated during the trial will be managed following two different processes, one related to clinical data, defined as all data reported in the CRF and all laboratory data, and one related to the data pertaining to SAEs (i.e. data reported by the Investigator on the SAE Reporting Forms and Complementary Information Forms).

Clinical Data Management

During the trial, through regular data collection and monitoring, clinical data reported in the CRFs will be integrated into the clinical database under the responsibility of sanofi pasteur Clinical Data Management platform. For each batch of data, double entry, quality control and triggers to computerized logic and/or consistency checks will be systematically applied in order to detect

errors or omissions. Medical safety data reviews may be performed several times during the course of the trial. Queries will be generated and submitted through Data Clarification Forms to the Investigator for resolution. Each step of this process will be monitored through the implementation of individual passwords and regular backups to maintain appropriate database access and to ensure database integrity.

The validation of the immunogenicity data will be performed at the laboratory level following the laboratory's procedures. Information from the laboratories such as subject identifiers and dates or sample numbers will be checked for consistency before the integration into the clinical database. All immunogenicity data, with the exception of those from the observational endpoints, will be provided by GCI, entered into GCI Laboratory Information Management Systems (LIMS) and quality control checked. These data will then be exported to Clinical Data Management for integration into the clinical database.

After integration of all corrections in the complete set of data, and after the SAE information available from Clinical Data Management and Pharmacovigilance has been reconciled, the database will be locked and saved before being released for statistical analysis.

SAE Data Management

During the trial, data pertaining to SAEs reported on SAE Reporting Forms, and Complementary Information Forms will be integrated into the Sponsor's centralized GPVD database.

Upon receipt of an SAE Reporting Form, the data will be entered into the GPVD database after a duplicate check. Each SAE is assigned a case identification number. Entered data will be independently verified against the original SAE forms. All SAEs are then reviewed by the Product Safety Officer and the CTL. Complementary Information Forms are issued and sent to the Investigator as required. Each SAE is reviewed, locked and approved in the GPVD database before being reported to the relevant authorities as necessary. Any follow-up information concerning a locked and approved SAE will be incorporated and a new version of the SAE will be created.

The data on SAEs in the Clinical Data Management database will be reconciled with those in the Pharmacovigilance database.

11 Statistical Methods and Determination of Sample Size

Data in the Clinical Data Management Database will be analyzed by the Biostatistics Platform of sanofi pasteur, France, with SAS software, Version 8.2 or above (SAS Institute, Cary, North Carolina, USA).

A first statistical analysis will be performed once all the data obtained 21 days after the second vaccination have been locked (first partial database lock), to address the safety and immunogenicity objectives of the D0 to D42 period.

A final statistical analysis will be performed when all the data obtained 21 days after the booster vaccination have been locked (second partial database lock), to address the immunogenicity and safety objectives for the booster vaccination and the Ab persistence objective.

An addendum will be produced after the final 6 month safety follow-up (final database lock).

11.1 Statistical Methods

All the main analyses will be descriptive; for the main parameters, 95% CIs of point estimates will be calculated using normal approximation for quantitative data and exact binomial distribution (Clopper-Pearson method) for proportions.

11.1.1 Hypotheses and Statistical Methods for the Safety Endpoints

11.1.1.1 Hypotheses

No statistical hypotheses will be tested.

11.1.1.2 Safety Analysis

The safety analysis will report the occurrence of solicited reactions and the incidence of unsolicited events over the safety observation period by vaccine formulation and by age group. The safety observation period is 21 days after each vaccination, and 7 days after each vaccination for the solicited reactions.

In order to avoid any under-estimation of the incidences, the number of subjects with documented safety will be used as denominator of the frequencies. For solicited reactions, safety summary parameters and EMEA safety criteria, 95% CIs of point estimates of proportion will be calculated using the exact binomial distribution (Clopper-Pearson method) for proportions (23).

Safety combined for the 21 days after the first two vaccinations and after each separate vaccination (including booster):

The analyses of safety will address the number and percentage of subjects, by vaccine formulation and by age group, experiencing injection site or systemic AEs until 21 days after each injection (solicited reactions from D0 to D7 and unsolicited AEs/reactions until D21).

The number and percentage of subjects experiencing the following will be described by vaccine formulation and by age group:

- Each solicited and unsolicited (MedDRA preferred term) reaction or event after each injection.
- Each solicited reaction (from 0 to 7 days) after each injection according to severity, time to onset, and number of days of occurrence.
- Each unsolicited adverse reaction (MedDRA preferred term) (until 21 days) after each injection according to severity, time to onset, and duration.

(Specific solicited reactions for each age group will be presented separately).

Additionally, for subjects aged ≥2 years, the number and percentage of subjects experiencing the following reactions (MedDRA Preferred Terms) in the 3 days following each injection will be reported by vaccine and age group after each injection (as defined in the Note for Guidance on Harmonization Requirements for Influenza Vaccines [CPMP/BWP/214/96]):

- Injection site induration >5 cm observed for more than 3 days.
- Injection site ecchymosis (hemorrhage).

- Pyrexia: rectal equivalent temperature >38°C for ≥24 hours (pyrexia).
- Malaise.
- Shivering (chills).

The number and percentage of subjects in each group experiencing SAEs will be collected throughout the study.

11.1.2 Hypotheses and Statistical Methods for the Immunogenicity Endpoints

11.1.2.1 Hypotheses

No statistical hypotheses will be tested for the immunogenicity endpoints analysis.

11.1.2.2 Statistical Analysis

11.1.2.2.1 Immunogenecity Analysis of the Primary Series Vaccination

The point estimates and their 95% CI of the parameters corresponding to the following endpoints will be presented for each vaccine formulation and age group (a total of 8 groups):

HI titers[a] (1/dil) with Turkey or Horse Erythrocytes:

- Proportion of subjects with a detectable titer (i.e. titer>10 [turkey] or >8 [horse] [1/dil]) on D0[b], D21, and D42.
- GM of titers on D0, D21, D42.
- GM of individual titers ratio (GMTR) D21/D0, D42/D0, and D42/D21.
- Proportion of subjects with a titer ≥40 (turkey) or ≥32 (horse) (1/dil) at D0, D21, and D42.
- Seroconversion (defined as initially seronegative subjects, i.e. with <10 [turkey] or <8 [horse] [1/dil] pre-vaccination titer, with a post-vaccination titer ≥40 [turkey] or ≥32 [horse] [1/dil])

or

- Significant increase rate (defined as initially seropositive subjects i.e. with ≥10 [turkey] or >8 [horse] [1/dil] pre-vaccination titer, with at least a four-fold increase in post-vaccination titer) from D0 to D21, D0 to D42 and from D21 to D42.

Seroneutralization (1/dil):

- Proportion of subjects with detectable neutralizing Ab (i.e. SN titer ≥20 [1/dil]) on D0[c], D21, and D42.

---

[a] Individual geometric means of duplicates

[b] In order to check the baseline naïve immunogenicity

[c] In order to check the baseline-naïve immunogenicity

- GM of SN titer on D0, D21, and D42.
- GMTR: D21/D0, D42/D0, and D42/D21.
- Two- and four-fold increase rate from D0 to D21, from D0 to D42 and from D21 to D42.

11.1.2.2.2 Methods for Statistical Analysis

For all titration methods

Geometric mean computation: Assuming that the Log_{10} transformation of the measurements follows a normal distribution, at first, the mean and 95% CI will be calculated on Log_{10} measurements using the usual calculation for normal distribution, then antilog transformations will be applied to the results of calculations, in order to provide GMs and their 95% CIs.

95% CIs computation will be performed according to:

- The normal approximate method for CIs of GMs (GMTs and GMTRs).
- The exact binomial distribution (Clopper-Pearson method) for CIs of proportions (24).

11.1.2.2.3 Exploratory Statistical Analyses

For each serological method, the following may be performed as exploratory analyses:

- Describe the titers frequency of individual titers with possible non-parametric exploratory comparisons (robustess analysis, alternative to the GMT approach)
- Assess and quantify the second vaccination relative effect.
- Check any age-group heterogeneity.
- Confirm any gender effect on the immunogenicity parameters.

The exploratory analyses will be detailed in the Statistical Analysis Plan prior to the first database lock.

11.1.2.2.4 Pre-booster Antibody Persistence

For each serological method, the statistical analysis will be restricted to:

- Proportion of subjects with detectable titer (for HI method: titer >10 [turkey] or >8 [horse] [1/dil] and for SN method: titer ≥20 [1/dil]) on D90, M3 and M6 or at M3, M6 and M12.
- GM of titers on D90, M3 and M6 or at M3, M6 and M12.
- Proportion of subjects with a HI titer ≥40 (turkey) or ≥32 (horse) (1/dil) on D90, M3 and M6 or at M3, M6 and M12.

The kinetics of the Ab titers assessed by HI, methods will be studied and summarized graphically:

- GM of anti-HA titers (HI method using horse or turkey erythrocytes) at M3 and M6 or at M3, M6 and M12 (*depending on the booster vaccination timing option*).

- GM of Ab titers (seroneutralisation method) at M3 and M6 or at M3, M6 and M12 (*depending on the booster vaccination timing option*).

11.1.2.2.5 Booster Vaccination Immunogenicity

For each serological method, HI (HI method using horse and turkey erythrocytes) and SN, the analysis will be similar to the primary statistical analysis (see Section 11.1.2.2.1) at M6 or M12 (booster vaccination) and M6+21 days or M12+21 days (*depending on the booster vaccination timing option*).

11.1.2.2.6 Cellular Mediated Immune Response

The analysis of the CMI response will be described in the Statistical Analysis Plan (SAP).

11.2 Population to be Analyzed

11.2.1 Definition of Populations

11.2.1.1 Immunogenicity Analysis Set

The immunogenicity analysis set (IAS) is defined for the immunogenicity descriptive analyses of the primo-vaccination, the Ab persistence, and the booster vaccination.

The IAS is defined as the subset of subjects who received the first vaccine injection.

The analysis of the primo-vaccination (see Section 11.1.2.2.1) addresses endpoints involving pre- and post-injection titers at D0, D21 and D42 for HI, SN methods. The analysis will include all available data.

Data from subjects excluded from this analysis set will be listed separately and the reasons for exclusion, as well as the circumstances of the missing data, will be examined.

Additionally, for the booster analysis, the IAS is defined as subjects having also received the booster injection.

The analysis will be performed according to the randomized vaccine formulation group.

11.2.1.2 Per-Protocol Analysis Set

Not applicable.

11.2.1.3 Safety Analysis Set

The safety analysis set (SafAS) is defined as those subjects who received at least one dose of study vaccine. A safety analysis set is defined for each dose as the subset of subjects who received the dose.

Safety results will be presented according to the schedule received.

Subjects who received an inconsistent vaccination schedule (e.g. two primary injections of different dosages) will be assigned to the group determined by the first injection received. Their safety results after the second injection will be excluded from all safety analyses, and will be listed separately.

The SafAS for the booster vaccination is defined as those subjects who received at least one injection of the primo vaccination.

11.2.2 Populations Used in Analyses

The populations to be used in the analyses are presented in the table below.

Table 11.1: Populations for Analyses

<table>
<tr><th>Objectives</th><th colspan="2">Description and Endpoints</th><th>Included and randomized subjects</th></tr>
<tr><td rowspan="2">Safety objectives</td><td colspan="2">Primo vaccination safety (solicited and unsolicited safety events)</td><td>SafAS</td></tr>
<tr><td colspan="2">Booster vaccination safety and safety follow-up (solicited and unsolicited safety events)</td><td>SafAS</td></tr>
<tr><td rowspan="3">Immunogenicity objectives</td><td>Primary series vaccination [D0-D42]</td><td rowspan="3">- HI (horse erythrocytes)
- HI (turkey erythrocytes)
-Seroneutralisation</td><td>IAS</td></tr>
<tr><td>- Ab persistence</td><td>IAS</td></tr>
<tr><td>- Booster vaccination</td><td>IAS (available titers at pre- and post-booster timepoint)</td></tr>
<tr><td>Immunogenicity observationnal objectives</td><td colspan="2">CMI response</td><td>CMI subset of IAS</td></tr>
</table>

11.3 Determination of Sample Size and Power Calculation

The overall study cohort (N=240) will provide a probability >90% of detecting any AE with an incidence of 1% in the study, and a probability of 70% for each pooled adjuvanted and non-adjuvanted group (N=120).

11.4 Interim Analysis

No formal interim statistical analysis is planned.

12 Ethical and Legal Issues and Investigator/Sponsor Responsibilities

12.1 Ethical Conduct of the Trial / Good Clinical Practice

This trial will be conducted in accordance with the Edinburgh revision of the Declaration of Helsinki as far as adopted by the concerned regulatory authorities, as well as ICH Good Clinical Practice, the applicable national and local requirements regarding ethical committee review, informed consent, and other statutes or regulations regarding the protection of the rights and welfare of human subjects participating in biomedical research.

12.2 Source Documents and Source Data

The purpose of source documents is to document the existence of the subject and substantiate the integrity of the trial data collected. The Investigator must maintain the trial source documents accurate, complete, legible and up to date.

Examples of source documents are: Subject Contact, Screening, and Enrolment Log, Clinical and Office Charts, Subject's diaries, hospital records, informed consent forms, investigational dispensing and reconciliation forms, subject's file and records kept at the pharmacy or at the laboratories, mail, certified letters.

The Subject Contact, Screening, and Enrolment Log should list all subjects who contacted the Investigator or were contacted by the Investigators to participate in the trial, regardless of the outcome.

Source data are the data contained in source documents (originals or certified copies).

12.3 Confidentiality of Data and Access to Subject Records

Prior to initiation of the trial, the Investigator will sign a fully executed confidentiality agreement with the Sponsor.

Electronic medical records alone are acceptable only if validated computerized systems are used and are compliant with US 21 CFR Part 11. If this is not the case, the Investigator is obliged to print any records on an on-going basis, to sign and date them immediately after their creation, and to keep them on file as source documents which can be verified by the Sponsor or an inspector in comparison to the electronic records. Any later changes of the electronic records require the record to be reprinted, dated (with an indication of the date of change) and signed. Such records must also be kept together with the original printed copy.

Sponsor personnel, the IRB/IEC and the regulatory authorities will have direct access to source data/documents.

12.4 Monitoring, Auditing, and Archiving

12.4.1 Monitoring

Before the start of the trial (i.e. before the inclusion of the first subject by the first center), the Investigators and the Sponsor's monitoring staff will meet at the "Site-initiation visit" to discuss the trial protocol and the detailed trial procedures, with emphasis on inclusion and exclusion criteria, visit timing, safety procedures, informed consent procedures, SAE reporting procedures, CRF completion, and sample and product handling.

The Sponsor's monitoring staff will ensure and document that all material to be used during the trial has been received and that the investigational team and local monitoring staff have been properly informed about the trial, GCP and regulatory requirements, and the Sponsor's procedures. Specific training sessions for the investigational team and CRAs on these topics may be performed, as necessary.

Specific instruction manuals will be provided for the completion of the CRF (CRF Completion Guide) and for the detailed trial procedures such as the laboratory and sample handling procedures (Operating Guidelines).

After the start of the trial, the Sponsor's monitoring staff will be in regular contact with the investigational team through telephone calls and regular follow-up visits to the trial centers.

The Investigator must be available for these visits and will allow the monitoring staff direct access to subject medical files and CRFs. During monitoring visits, the monitoring staff will:

- Control the quality of the trial progress (e.g., with respect to the protocol and operating guidelines, quality of data collection and document completion, signature of consent forms, appearance of SAEs, sample and product management, cold chain monitoring, and archiving).
- Collect completed CRFs or CRF pages and any corresponding queries (Data Correction Forms).
- Evaluate the number of complete or ongoing observations.

Any identified problems will be discussed with the Investigator and corrective or preventive actions will be determined, as appropriate.

Once the CRF pages corresponding to the last visit have been returned duly completed and signed, the Investigator must be available to complete any queries (Data Correction Forms) forwarded by the Sponsor until database lock.

At the end of the trial, a close-out visit will be performed to ensure that:

- The center has all the documents necessary for archiving.
- All samples have been shipped to the appropriate laboratories.
- All unused material and products have been returned to the Sponsor.

12.4.2 Audits and Inspections

A quality assurance (QA) audit may be performed by the Sponsor's QA Department or by independent auditors to verify that the trial has been conducted according to the protocol, GCP, ICH requirements and the applicable regulations. An inspection may be conducted by regulatory authorities. The Investigator must allow direct access to trial documents during these inspections and audits.

12.4.3 Archiving

The Investigator must keep all trial documents for at least 15 years after the completion or discontinuation, whatever the nature of the investigational center (private practice, hospital, institution). The Investigator will inform sanofi pasteur of any address change.

The Sponsor, or subsequent owner, will retain all documentation pertaining to the trial for the lifetime of the product. Archived data may be held on microfiche or electronic record, provided that a back-up exists and that hard copy can be obtained if required. The protocol, documentation, approvals and all other documents related to the trial, including certificates attesting that satisfactory audit and inspection procedures have been carried out, will be kept by the Sponsor in the Trial Master File. Data on AEs are included in the Trial Master File. All data and documents will be made available if requested by relevant authorities.

12.5 Financial Contract and Insurance Coverage

An agreement will be signed by all the parties involved in the trial's performance, if relevant. Adequate insurance coverage for all subjects to be included in the trial is supplied by the Sponsor.

12.6 Stipends for Participation

The trial is set up in two centers. Transport expenses will be provided to subjects and parents/legal representatives as appropriate.

12.7 Publication Policy

Data derived from the trial are the exclusive property of the Sponsor. Any publication or presentation related to the trial must be approved by the Sponsor before submission of the manuscript. After publication of the results of the trial, any participating center may publish or otherwise use its own data provided that any publication of data from the trial gives recognition to the trial group. In addition, the Sponsor shall be associated with all such publications, it being understood that the Sponsor is entitled to refuse the association.

The Sponsor must have the opportunity to review all proposed abstracts, manuscripts or presentations regarding this trial at least 60 days prior to submission for publication/presentation. Any information identified by the Sponsor as confidential must be deleted prior to submission, it being understood that the results of this trial are not to be considered confidential.

The Sponsor review can be expedited to meet publication guidelines.

13 Reference List

1 Galasso GJ, Tyeryar FJ Jr, La Montagne JR. Overview of clinical trials of influenza vaccines, 1976. J Infect Dis. 1977;136 (Suppl):S425-8.

2 Treanor JJ, Wilkinson BE, Masseoud F, Hu-Primmer J, Battaglia R, O'Brien D, et al. Safety and immunogenicity of a recombinant hemagglutinin vaccine for H5 influenza in humans. Vaccine. 2001;19:1732-7.

3 Nicholson KG, Colegate AE, Podda A, Stephenson I, Wood J, Ypma E, et al. Safety and antigenicity of non-adjuvanted and MF59-adjuvanted influenza A/Duck/Singapore/97 (H5N3) vaccine: a randomised trial of two potential vaccines against H5N1 influenza. Lancet. 2001;357:1937-43.

4 WHO. Cumulative Number of Confirmed Cases of Avian Influneza A/(H5N1) Reported to WHO. http://www.who.int/csr/disease/avian_influenza/country/cases_table_2006_02_13/en/index.html (accessed 4 April 2006).

5 Webby RJ, Perez DR, Coleman JS, Guan Y, Knight JH, and Govorkova EA. Responsiveness to a pandemic alert: use of reverse genetics for rapid development of influenza vaccines. Lancet 2004; 363: 1099-103.

6 Ungchusak K, Auewarakul P, Dowell SF, Kitphati R, Auwanit W, Puthavathana P, et al. Probable person-to-person transmission of avian influenza A (H5N1). N Engl J Med. 2005; 352: 333-40.

7 The World Health Organization Global Influenza Program Surveillance Network. Evolution, of H5N1 Avian Influenza Viruses in Asia. Emerg Infect Dis 2005;11: 1515-21.

8 Schawrtz B and Gellin B. Vaccination strategies for an influenza pandemic. J Infect Dis 2005; 191: 1210-5.

9 Stephenson I, Nicholson KG, Wood JM, Zambon MC, and Katz JM. Confronting the avian influenza threat: vaccine development for a potential pandemic.Lancet Infect Dis 2004; 4: 499-509.

10 Webby RJ and Webster RG. Are we ready for pandemic influenza? Science 2003; 302: 1519-22.

11 Wood JM and Robertson JS. From lethal virus to live-saving vaccine: developing inactivated vaccines for pandemic influenza. Nat Rev Microbiol 2004; 2: 842-7.

12 Stohr K. The global agenda on influenza surveillance and control. Vaccine 2003; 21: 1744-8.

13 Stephenson I, Gust I, Kieny MP, and Pervikov Y. Development and evaluation of influenza pandemic vaccines. Lancet Infect Dis. 2006; 6:71-2.

14 CHMP Guideline on Dossier Structure and Content for Pandemic Influenza Vaccine Marketing Authorisation Application (CPMP/VEG/4717/03).

15 CHMP Guideline on Submission of Marketing Authorization Applications for Pandemic Influenza Vaccine Through the Centralised Procedure (CPMP/VEG/4986/03).

16 Bresson JL, Perronne C, Launay O, Gerdil C, Saville M, Wood J, et al. Safety and immunogenicity of an inactivated split-virion influenza A/Vietnam/1194/2004 (H5N1) vaccine: phase I randomised trial. Lancet 2006; May 20;367(9523):1657-64.

17 Seneviratne, U. Guillain-Barré Syndrome. Postgrad Med J 2000; 76: 774-782.

18 Safety and Immunogenicity of an Intramuscular, Inactivated, Split-Virion, Pandemic Influenza A/H5N1 Vaccine in Adults and the Elderly. Final Clinical Trial Protocol (GPA02) 16 Feb 2006; Version 2.0.

19 Stephenson I, Nicholson KG, Colegate A, Podda A, Wood J, Ypma E, et al Boosting immunity to influenza H5N1 with MF59-adjuvanted H5N3 A/Duck/singapore/97 vaccine in a primed human population. (2003) Vaccine 21: 1687-1693.

20 Kumagai T, Nagai K, Okui T, Tsutsumi H, Nagata N, Yano S et al. Poor immune responses ti influenza vaccination in infants. Vaccine 2004; 22: 3404-10.

21 Sasaki S, Jaimes MC, Holmes TH, Dekker CL, Makmood K, Kemble GW et al. Comparison of the Influenza Virus-Specific Effector and Memory B-Cell Responses to Immunization of Children and Adults with Live Attenuated or Inactivated Influenza Virus Vaccines. Journal of Virology, 2007: 81; 215-28.

22 Zeman AM, Holmes TH, Stamatis S, Tu W, He XS, Bouvier N et al. Humoral and Cellular Immune Responses in Children Given Annual Immunization With Trivalent Inactivated Influenza Vaccine. Pediatric Infectious Disease 2007; 26: 107-15.

23 Newcombe R.G., Two-sided confidence intervals for the single proportion: comparison of seven methods, Statistics in Medicine, (1998) 17, 857-872.

24 Ulanova M, Tarkowski A, Hahn-Zoric, M. The common vaccine adjuvant aluminum hydroxide up-regulates accessory properties of human monocytes via an interleukin-4-dependant mechanism. Infection and Immunity, 2001; 1151-59.

List of Appendices

Appendix 1 List of Investigators, Trial Centers and Other Personnel Involved in the Trial
Appendix 2 Sample Assent Forms and Sample Informed Consent Form
Appendix 3 Signature Page

Protocol Appendix 1: List of Investigators, Trial Centers, and Other Personnel Involved in the Trial

Protocol Appendix 1: List of Investigators, Trial Centers and Other Personnel Involved in the Trial

Principal Investigators	Investigator address	Center number, name and adress
Tawee CHOTPITAYASUNONDH, M.D<br>(Coordinating Investigator)	Same as center	Queen Sirikit National Institute of Child Health (Children's Hospital)<br>Bangkok<br>Thailand<br>Tel: +66 2460382 ext. 2404<br>Fax: +66 2460382
Usa THISYAKORN, M.D	Same as center	Chulalongkorn Hospital<br>Bangkok<br>Thailand<br>Tel: +66 22564000 ext. 3349<br>Fax: +66 22564930

sanofi pasteur involved personnel	Name
Clinical Team Leader	Melanie SAVILLE, MB BS
Clinical Scientist	Stéphanie PÉPIN
Clinical Research Associate (CRA)	Fabienne ROCHE
Statistician	Christophe CARRÉ
Product Safety Officer	Joao MAIO ALMEIDA

Protocol Appendix 2: Sample Assent Forms and Sample Informed Consent Form

ASSENT FORM
(for children aged 7 to 12 years ie from the 7th birthday to the day before the 13th birthday)

Safety and Immunogenicity of Two Different Formulations of an Intramuscular A/H5N1 Inactivated, Split Virion Pandemic Influenza Vaccine in Children (GPA04)

Name and Address of Investigator:	Dr. Tawee CHOTPITAYASUNONDH<br>Queen Sirikit National Institute of Child Health (Children's Hospital)<br>Bangkok, Thailand
Name and Address of Sponsor:	sanofi pasteur<br>2, avenue Pont Pasteur<br>F-69367 Lyon cedex 07<br>France

You are being invited to be part of a research trial. A research trial tries to find better ways to treat or prevent diseases. It is important that you read all of this form before you decide if you want to be in this trial or not.

If you don't understand the information on the form, please ask your parents or the person who looks after you or the doctor and they will explain it to you. Lots of other children are also being asked if they would like to be in this trial.

This trial is to try and find a vaccine that could prevent a possible outbreak of pandemic influenza all over the world called H5N1.

A vaccine is a special type of medicine that helps to protect you against getting sick from germs. The H5N1 germ that we are interested in may make children and grown-ups ill with things like headaches, fever, and feeling sick. In some serious cases people could get very ill and have to go to hospital. We want to try and find out how we can prevent this happening.

At the moment we don't know if this vaccine would stop you getting ill from this germ. So we want to test the vaccine to see whether it could protect you and also to make sure that giving you the vaccine itself does not make you feel ill.

If you agree to be in the trial, you will visit the trial center either nine or ten times over the next one to one and a half years.

You will receive three injections of the vaccine into your arm.

The first two injections will be about three weeks apart. The third injection will be after half a year or a year.

The doctor will take six or seven blood samples to learn about how the vaccine works in your body. The doctor will take these blood samples just before and a little bit after each injection. Each blood sample will be about a two teaspoonfuls.

During the visits, the doctor may also take your temperature under your armpit, examine you, and ask you or your parents or the person who looks after you questions about your health.

After the vaccinations you may get a little bruise on your arm. You may also feel a bit unwell for a couple of days.

Blood samples will be taken from your other arm using a needle and syringe. The needle may hurt a little bit, but the hurt will go away after a little while. You may get a little bruise on your arm. If you feel bad, you should tell the doctor or your parents or the person who looks after you.

In between the times when you visit the doctor your parents or the person who looks after you will also take your temperature under your armpit. This information will be collected the next time you visit the doctor.

The doctor will also ask your parents or the person who looks after you to give permission for you to be in the trial. You will only be able to take part if both you and your parents or the person who looks after you agree. If one of you does not want to agree, then you will not be able to take part.

In addition, we would also like to keep any spare part of your blood samples for possible use in the future. If you don't want to agree to this, you can still agree to take part in the rest of the trial.

If you sign your name at the bottom of this form, it means that you agree to take part in the trial. We will give you and your parents or the person who looks after you a copy of this form after you have signed it.

Assent for participating in the trial

- I have read all of this form.
- I have been allowed to ask questions about the trial.
- All of my questions have been answered and I am satisfied with the answers I received.
- I will have a copy of this signed form to keep for myself.
- If I change my mind after agreeing to be in the trial, I will tell the doctor right away.
- No one will be upset if I don't want to be in the trial.
- I agree to be in the trial.

Specific consent statement for the future use of your blood samples in research:

Please indicate your choice by ticking **one** of the two boxes below:

☐ I agree to the future use of my blood samples for future research.

<u>OR</u>

☐ I agree the use of my blood samples for doing tests related to this trial *BUT* do *NOT* agree to the future use of my blood samples for any other research.

Name of the subject:

____

Signature of the subject:

____

Date:

____

Name and signature of person explaining the assent:

____

Name and signature of person collecting the assent (if different):

____

Date:

____

ASSENT FORM
(for subjects aged 13 to 17 years, ie from the 13th birthday to the day before the 18th birthday)

Safety and Immunogenicity of Two Different Formulations of an Intramuscular A/H5N1 Inactivated, Split Virion Pandemic Influenza Vaccine in Children (GPA04)

Name and Address of Investigator: ____________________________________

____

____

____

Name and Address of Sponsor: sanofi pasteur
2, avenue Pont Pasteur
F-69367 Lyon cedex 07
France

You are being asked to agree to take part in a research trial to help to develop a vaccine against a pandemic influenza virus, which is being conducted in Thailand.

Although this vaccine has been approved for use in this particular trial, it has not been licensed for use in Thailand. No guarantee or assurance can be made regarding the results of this trial.

This trial is being paid for by sanofi pasteur (the rest of this form will refer to sanofi pasteur as "the Sponsor"). Dr. ………………….. is the "investigator" for the trial, and his/her institution will receive a research grant for the conduct of this trial. The Ethics Committees (EC)/Institutional Review Boards (IRB) of your center approved this trial.

This document will provide you with the information needed to help you decide whether you wish to take part in this trial. If any part or word of this document is unclear, or if you have any questions or want additional information at any time, please do not hesitate to ask one of the trial team members.

What is Pandemic Influenza?

Since December 2003, a growing number of Asian and Eastern European countries have reported outbreaks of avian influenza in chickens and ducks, resulting in the death or culling of more than 100 million poultry. The A/H5N1 strain has been the cause of most of these outbreaks.

Between December 2003 and mid-July 2006, A/H5N1 viruses have caused at least 230 confirmed cases (including 132 deaths) in human adults and children in Vietnam, Thailand, Indonesia, China, Cambodia, Turkey, Iraq, Azerbaijan, Djibouti and Egypt. To date, people who have been

infected with the A/H5N1 virus have been in direct contact with infected and sick birds. Until recently, the virus had not been infectious between humans, but a recent bird-flu outbreak in an Indonesian village has raised the level of concern that the virus may be able to pass directly between people since no animal has been identified as the source of the infection. This would be the first known three-person chain of human-to-human transmission.

There is a concern that the A/H5N1 virus, if given sufficient opportunity, will mutate into a form that is highly infectious and for which humans would have no natural immunity (that is, the population would have no antibody [substances that protect the body from infections] in the blood to protect against the virus). Such a virus could spread easily from one person to another, resulting in pandemic influenza.

Past influenza pandemics have been associated with the sudden onset of severe typical influenza symptoms such as headache, high fever, muscle and joint aches, reduced appetite, nausea, vomiting and cough, which typically last for 2 to 4 days. Some patients, after an initial recovery, subsequently develop pneumonia, and although most patients ultimately recover, some die rapidly due to breathing difficulties.

What vaccines or other treatments may be used to prevent or treat Pandemic Influenza?

In the event of a pandemic, it will be possible to use antiviral drugs, which are commonly used to treat influenza each year (for more information, ask your doctor), to control pandemic influenza and to treat those infected with the virus however the effectiveness of anti-viral drugs in the treatment of pandemic influenza has yet to be demonstrated. Vaccination may be effective at preventing pandemic influenza but as with antiviral drugs the use of an A/H5N1 vaccine in an A/H5N1 pandemic has to date not been demonstrated.

What is the purpose of this trial?

This is a medical research trial, the purpose of which is to test two influenza pandemic vaccine formulations as a two-primary dose schedule plus a booster vaccination in A/H5N1-naïve subjects (that is, subjects who have not previously been exposed to the virus contained in the vaccine) aged from 6 months to 17 years. Subjects will be studied in three age groups (9 to 17 years, 3 to 8 years, and 6 to 35 months). The influenza pandemic vaccine is produced from an avian influenza strain, specifically an A/H5N1 virus.

A similar influenza vaccine has already been injected in 300 naïve adults (aged 18 to 40 years) in a trial conducted in France, and in a further 300 naïve adults (aged 18 to 60 years) and 300 elderly subjects (aged >60 years) in a second trial in Belgium and England. Based on the results of the first trial, a 30 µg dose of vaccine with adjuvant (an adjuvant is a component of many vaccines, which is added to enhance the immune response) and 7.5 µg dose of vaccine without adjuvant were selected for use in the second trial. In the first trial, each vaccine formulation appeared to be generally safe and well tolerated and the highest antibody response was observed following vaccination with the 30 µg dose with adjuvant.

The objectives of the present trial are to describe the safety and the immune response following administration of two vaccinations (separated by 21 days) followed by a booster vaccination. The booster dose will be administered 6 or 12 months after the first vaccination. A final decision regarding the time of administration of the booster (6 or 12 months) and its formulation will be made following a review of further data from the previous studies.

How will this trial be done?

This trial will consist of nine (design with booster at 6 months) or ten (design with booster at 12 months) visits (although the final 'visit' may be conducted by telephone) and will last 12 or 18 months, respectively. It will involve 60 subjects aged 9 to 17 years, 60 subjects aged 3 to 8 years, and 120 subjects aged 6 to 35 months. For subjects aged 3 to 8 years and 9 to 17 years, 30 subjects in each age group will receive each formulation (7.5 µg without adjuvant and 30 µg with adjuvant); for subjects aged 6 to 35 months, 30 subjects will receive each formulation and in addition 30 subjects will receive a half-dose of each formulation (ie 3.75 µg without adjuvant and 15 µg with adjuvant). The trial will be performed in Thailand.

At the start of the trial, you will be assigned by chance to receive one of the selected formulations and will receive two injections of the same vaccine formulation, separated by an interval of 21 days, and a third (booster) vaccination at 6 or 12 months after the first vaccination.

At each visit, the Investigator will examine you and will ask you questions about your health, and you will be asked to record any unwanted medical effects that could occur between these visits. In addition, a blood sample (7 mL [approximately 2 teaspoons]) will be taken from you at each vaccination visit, at the visits prior to and 21 days after vaccination, and at 3 months after the first vaccination to measure the effect of the vaccinations on the amount of antibodies that you develop.

Once all the results of the previous studies are available, the decision to administer a booster vaccination at either 6 or 12 months after the first vaccination, and the formulation to be used, will be made. Both options for a booster at 6 months or 12 months are presented below and you will be informed of the decision once this information is available.

- First visit:

All the risks and benefits associated with the trial will be explained to you before you sign the following informed consent statement. After having received satisfactory answers to any questions you may have, and if you agree to take part, you will sign the Assent Form.

The Investigator will examine you and check your medical history to make sure that you can take part in the trial. This involves a physical examination, a urine pregnancy test (if appropriate), and recording of your medical history (especially regarding any previous influenza-like illness, any previous influenza vaccination, any allergic reactions to previous vaccinations or any allergy to egg proteins). You will be asked not to participate in another research trial for the whole period of the present trial.

If you are eligible for the trial, 7 mL (approximately 2 teaspoons) of blood will be taken in order to measure your level of antibodies before vaccination. You will then receive an injection of one of the two vaccine formulations into your upper-arm muscle. To ensure your safety, you will stay at the trial center under medical observation for 30 minutes following vaccination.

You will then be given a digital thermometer, a ruler, and a diary card, together with instructions on their use, to note any unwanted effects that may happen to you between vaccination and the next visit. On the evening of vaccination and on each of the next 7 days, you will be asked to record your temperature under your arm pit and any specified symptoms commonly observed after any vaccine administration. You will also be asked to record any medications (other than the vaccine) that you may take/be given during the 7 days following the vaccination. In addition, any

non-specified symptoms occurring between the vaccination and the second visit should be recorded in the diary card.

- Second visit: 8 days after the first vaccination:

The Investigator will examine you, review with you the diary card that you will have completed since the first vaccination and will question you on your health since vaccination.

- Third visit: 21 days after the first vaccination:

The Investigator will examine you, review with you the diary card that you will have completed since the second visit and will question you on your health since the second visit. A urine pregnancy test will be performed if appropriate. A 7 mL (approximately 2 teaspoons) blood sample will be taken from you to test the effect of the vaccine on your body's immune system. Then you will receive the second vaccine injection into your upper-arm muscle. To ensure your safety, you will stay at the trial center under medical observation for 30 minutes following vaccination.

You will then be given a second diary card to note any medications that you may take/be given and unwanted effects that may happen between the second vaccination and the next visit.

- Fourth visit: 8 days after the second vaccination:

The Investigator will examine you, review with you the diary card that you will have completed since the second vaccination and will question you on your health since vaccination.

- Fifth visit: 21 days after the second vaccination:

The Investigator will examine you, review with you the diary card that you will have completed since the previous visit and will question you on your health since the previous visit. A blood sample (7 mL [approximately 2 teaspoons]) will be taken in order to further investigate the effect of the vaccine on your body's immune system.

A memory aid will be provided to help you to record any important events until the next visit.

- Sixth visit: 3 months after the first vaccination:

The Investigator will examine you, review with you the memory aid that you will have completed since the previous visit and will question you on your health since the previous visit. A blood sample (7 mL [approximately 2 teaspoons]) will be taken in order to further investigate the effect of the vaccine on your body's immune system.

Trial design with a 6-month booster design: seventh visit to ninth visit:

- Seventh visit: 6 months after the first vaccination:

The Investigator will examine you, review with you the memory aid that you will have completed since the previous visit and will question you on your health since the previous visit. A urine pregnancy test will be performed if appropriate. A blood sample (7 mL [approximately 2 teaspoons]) will be taken from you in order to further investigate the effect of the vaccine on your body's immune system. Then you will receive the third vaccine dose (booster vaccination) into your upper-arm muscle. To ensure your safety, you will stay at the trial center under medical observation for 30 minutes following vaccination.

You will then be given a third diary card to note any medications that you may take and unwanted effects that may happen between the booster vaccination and the next visit.

- Eighth visit: 21 days after the booster vaccination:

The Investigator will examine you, review with you the diary card that you will have completed since the previous visit and will question you on your health since the previous visit. A blood sample (7 mL [approximately 2 teaspoons]) will be taken from you in order to further investigate the effect of the vaccine on body's immune system.

A second memory aid will be provided to help you to record any important events until the next visit.

- Ninth visit (or telephone call): 12 months after the first vaccination:

The Investigator will examine you (or interview you over the telephone), review with you the memory aid that you will have completed since the previous visit and will question you on your health since the previous visit. This visit will mark the end of your participation in the trial.

Trial design with a 12-month booster design: seventh visit to tenth visit:

- Seventh visit: 6 months after the first vaccination:

The Investigator will examine you, review with you the memory aid that you will have completed since the previous visit and will question you on your health since the previous visit. A blood sample (7 mL [approximately 2 teaspoons]) will be taken from you in order to further investigate the effect of the vaccine on your body's immune system.

A second memory aid will be provided to help you to record any important events until the next visit.

- Eighth visit: 12 months after the first vaccination:

The Investigator will examine you, review with you the memory aid that you will have completed since the previous visit and will question you on your health since the previous visit. A urine pregnancy test will be performed if appropriate. A blood sample (7 mL [approximately 2 teaspoons]) will be taken from you in order to further investigate the effect of the vaccine on your body's immune system. Then you will receive the third vaccine dose (booster vaccination) into your upper-arm muscle. To ensure your safety, you will stay at the trial center under medical observation for 30 minutes following vaccination.

You will then be given a third diary card to note any medications that you may take and unwanted effects that may happen between the booster vaccination and the next visit.

- Ninth visit: 21 days after the booster vaccination:

The Investigator will examine you, review with you the diary card that you will have completed since the previous visit and will question you on your health since the previous visit. A blood sample (7 mL [approximately 2 teaspoons]) will be taken from you in order to further investigate the effect of the vaccine on your body's immune system.

A third memory aid will be provided to help you to record any important events until the next visit.

- Tenth visit (or telephone call): 18 months after the first vaccination:

The Investigator will examine you (or interview you over the telephone), review with you the memory aid that you will have completed since the previous visit and will question you on your health since the previous visit.

This visit will mark the end of your participation in the trial.

What are the risks and possible unwanted effects of vaccination and blood sampling?

Influenza vaccines are usually well tolerated when injected into a muscle. However, as with the administration of any other vaccine, you may experience a variety of unwanted events at the injection site. The most frequently observed local reactions in a previous study were pain, erythema (red skin) and induration (a lump in the skin) and the most frequently reported solicited systemic reactions (general reactions) in all groups and for each injection were headache and muscle and joint pain. You may also feel unwell or experience high temperature (39°C to 40°C), or malaise. These effects usually disappear without treatment within 3 days. This is not a complete list of possible effects.

Since influenza vaccines have been marketed, some other reactions such as itching, urticaria, or redness over the entire body have been observed infrequently (between 1/100 and 1/1000 vaccinees). Nerve pain, paresthesia (feeling of "pins and needles"), convulsions, and temporary low blood platelet count have occurred rarely (between 1/1000 and 1/10,000 vaccinees). Very rare reactions like neurological disorders have been reported (in less than 1 out of 10,000 vaccinees). As with any vaccination, there is a rare possibility of an allergic reaction. This may cause a severe narrowing of the air passages and breathing difficulties. If this occurs, you will need immediate medical attention. This is why you is required to remain under medical observation in the trial center for 30 minutes after each vaccination.

In addition, the sampling of blood from a vein may cause some pain, bruising at the puncture site, or lightheadedness.

There may be also other risks for you or, if appropriate, for your unborn child, should you become pregnant during the trial, that are not yet known. If you are pregnant at the moment, inform the Investigator. If you become pregnant between the first visit and the booster visit, the Investigator will withdraw you from the trial but will follow you until the end of your pregnancy to monitor your health and that of your baby.

What are the possible benefits for you?

The efficacy of the vaccine has not yet been established and there is no certainty that the influenza strain used in this vaccine will protect against a pandemic strain. As such, no benefit can be guaranteed.

What will you have to do?

If you agree to take part in this trial, you will have to attend the nine (design with booster at 6 months) or ten (design with booster at 12 months) planned visits. As described above, after each vaccination, you will be given a diary card to note any medical events or symptoms that may appear after vaccination until 21 days following vaccination, as well as any medication taken during that time. You will also have to measure your temperature under your arm pit in the

evening following each vaccination and for the seven subsequent days, and at any time that you feel feverish.

You or a family member must inform the Investigator as soon as possible if a serious medical event occurs, e.g. if you are admitted to hospital or visit an emergency department.

Sexually active female subjects will have to use an effective method of birth control for at least 4 weeks prior to and 4 weeks after each vaccination (including the booster vaccination at 6 or 12 months). You will have to inform the investigator as soon as possible should you become pregnant. If you plan to become pregnant during the next 12 or 18 months, you should not participate in the trial.

Who will see your medical and personal information?

Personal information collected during the trial will be confidential (with the exception of the situation described in the paragraph below); this confidential information will be computerized and sent to the Sponsor, and, if necessary, to authorized regulatory authorities. The personal information that will be collected includes medical data, date of birth, and age. You may exercise your right to access and correct your data. In these files, your name will be coded using your initials and a trial number. This is to protect your privacy. Your name and other information identifying you will not be forwarded to the Sponsor.

In addition to the use of computerized information as described above, any personal records, including your medical records and health history, relating to this trial may be inspected in the Investigator's offices by authorized representatives of the Sponsor, or by the health authorities of any country where the trial vaccine may be considered for approval. Absolute confidentiality of your records can therefore not be guaranteed.

The results of this trial may be presented at meetings or in publications. Your identity will not be revealed in these presentations.

What happens if you refuse to take part in the trial or change your mind after you agree?

Your decision regarding your participation in this trial is entirely voluntary. If you do not want to take part then you do not have to agree to your participation. You also have the right to agree to your participation and subsequently to change your mind. You can withdraw yourself from the trial at any time. If you withdraw yourself from the trial, the blood samples collected before your withdrawal will be used unless if you specifically request otherwise.

Whatever your decision, you will continue to qualify for full medical care without any penalty or loss of benefits to which you are otherwise entitled.

The Investigator or Sponsor may withdraw you from this trial for any reason at any time even without your consent, for example if the Investigator decides that it is in your best interest, or if you do not follow the trial instructions.

If you do decide to withdraw yourself from the trial, or if you are withdrawn by the doctor, then the Investigator may recommend that certain laboratory tests or physical examinations should be done to ensure your safety and well-being.

What else do you need to know before agreeing to take part in this trial?

Taking part in this trial is entirely voluntary. You will be provided with any new relevant information during the trial that may affect your willingness to continue your participation.

The investigator will inform your general practitioner about your participation in this trial unless you do not consent to this.

This trial has been designed and will be carried out in accordance with the ICH/GCP guidelines laid down in the Declaration of Helsinki for the protection of individuals participating in clinical trials.

You will receive a copy of this signed Assent Form. Please keep it safe and use it for information and reference throughout the trial.

If you are ill or injured during the study and it is determined that this was due to the vaccination or your study participation, you will be compensated for all reasonable outpatient costs of medical evaluation and treatment or hospitalization that are directly related to this illness or injury. In any case, and whatever the cause of the illness or injury, you will qualify for the usual standard of medical care that you would have received had you not participated in this trial.

The Sponsor has an insurance policy covering study-related illness or injury.

You will be re-imbursed for travel expenses associated with your participation in this trial.

Do not sign this Assent Form or agree to your participation in this trial unless you are comfortable with the risks involved and you have had an opportunity to ask questions and feel you have received satisfactory answers.

What will happen to your blood samples?

As a part of this trial, blood samples will be taken to test the effect of the pandemic influenza vaccine on your body's immune system. These tests will be done by the Sponsor or by another laboratory for the Sponsor. Your samples will be shipped out of Thailand for these tests and will be stored in a freezer until these tests are done. Therefore, you should only agree to participation in this trial if you agree to this use of your blood samples.

Any unused part of your blood samples will be securely stored by the Sponsor in the USA. They will be stored until at least 10 years after the vaccine being tested has been approved for use by the health authorities.

What should you know about possible future use of your samples for research?

The Sponsor would also like to keep any unused part of your blood samples for use in other evaluation studies, e.g. studies of the immune response to influenza vaccines, improvement of knowledge and documentation of the safety of this vaccine by using new developed laboratory methods or applying newly discovered concepts. You will not be informed about the types of research that will be conducted or any of the results, although, genetic tests will never be performed on these samples.

You have the right to agree to participation in the trial proposed to you today and still refuse to allow the Sponsor keep any unused blood samples for future use. You will be asked to decide

whether or not you permit this future use of your blood samples and to indicate your decision on the Assent Form.

You will not be paid for allowing the Sponsor to keep and later use any remaining part of your blood samples.

Should you refuse to allow the Sponsor to keep your unused blood samples then they will not be used for any testing other than that directly related to this trial.

Who can you contact if you have questions?

If you have any questions about this trial or if you want more information on compensation or medical treatment in the case of trial injuries, please contact:

Name: ……………….

Tel. Number: …………………..

If you have any questions about your rights as a subject in this trial, please contact:

Name: ……………….

Tel. Number: …………………..

Informed consent statement for participating in the trial

Subject's Last and First Names: ____

Subject's Entry Number: |0|0|-|0|0| | | | | Subject's Initials: |__| |__| |__|

By signing this form I certify to all of the following:

- I have read this entire Assent Form (or had the information read to me) and understand what will be done to me and what I am being asked to do.
- I consent to take part in this trial.
- I consent to make my confidential personal information available for review (direct access) to the Sponsor's representative or to any Competent Authorities, Institutions or governmental agencies assigned this task in this country or in another country where the trial vaccine may be considered for approval, or if applicable, the Ethics Committee/Institutional Review Board.
- I authorize the Investigator and Sponsor to have access to the medical records if I am admitted to a hospital or visit an emergency ward for any reason during the trial period.
- I understand and accept that information related to me collected during the trial will be coded so that my name does not appear in my record that is computerized and shared with the Sponsor. I am aware that my computerized information may be handled in another country. I am aware that I may exercise my right of access and correction of these data at any time with the Investigator.
- I have had the opportunity to ask questions and have received satisfactory answers, and I understand that I will receive a signed copy of this Assent Form.
- I understand that I may ask additional questions about this trial at any time.
- I understand that I am free to withdraw from the trial at any time without justifying my decision to do so and without it affecting my medical care.
- I understand that if I withdraw my consent, my blood samples collected before my withdrawal will be used unless I specifically request otherwise.
- I authorize the shipment of my blood samples to the Sponsor's laboratory (or other location as applicable) for doing tests related to this trial, and I understand that the laboratory may be in another country.

Specific consent statement for the future use of your blood samples in research

Please indicate your choice by checking one of the two boxes below:

☐ I authorize the future use of my blood samples for future research by the Sponsor under the conditions described in this information sheet (see the paragraph called "*What should you know about possible future use of your* blood samples*for research*").

OR

☐ I authorize the use of my blood samples for doing tests related to this trial *BUT DO NOT* authorize the future use of my blood samples for any other research.

Subject's signature	Date

Last and first name of witness
(only required the subject is illiterate)

Signature of witness<br>(only required if the subject is illiterate)	Relationship	Date

Investigator's statement (or person performing the Informed Consent procedure)

I certify that I have explained to the above individual the nature and purpose of the trial, potential benefits, and reasonably foreseeable risks associated with participation in this research trial. I have answered any questions that have been raised and have witnessed the above signature. I have explained the trial, as described in the Assent Form, to the above subject on the date stated on this Assent Form.

Last and first name and signature (of the person performing the Informed Consent procedure) — Date

Signature of Investigator (if different from the above and if required by local regulations) — Date

INFORMED CONSENT FORM
(for parents/legal representatives of all subjects)

Safety and Immunogenicity of Two Different Formulations of an Intramuscular A/H5N1 Inactivated, Split Virion Pandemic Influenza Vaccine in Children (GPA04)

Name and Address of Investigator: ____

____

____

____

Name and Address of Sponsor: sanofi pasteur
2, avenue Pont Pasteur
F-69367 Lyon cedex 07
France

As parents/legal representatives, you are being asked to volunteer your child to take part in a research trial to help to develop a vaccine against a pandemic influenza virus, which is being conducted in Thailand.

This form is to be signed by all parents/legal representatives to indicate consent for your child's participation in the trial.

Although this vaccine has been approved for use in this particular trial, it has not been licensed for use in Thailand. No guarantee or assurance can be made regarding the results of this trial.

This trial is being paid for by sanofi pasteur (the rest of this form will refer to sanofi pasteur as "the Sponsor"). Dr. ………………….. is the "investigator" for the trial, and his/her institution will receive a research grant for the conduct of this trial. The Ethics Committees (EC)/Institutional Review Boards (IRB) of your center approved this trial.

This document will provide you with the information needed to help you decide whether you wish for your child to take part in this trial. If any part or word of this document is unclear, or if you have any questions or want additional information at any time, please do not hesitate to ask one of the trial team members.

What is Pandemic Influenza?

Since December 2003, a growing number of Asian and Eastern European countries have reported outbreaks of avian influenza in chickens and ducks, resulting in the death or culling of more than 100 million poultry. The A/H5N1 strain has been the cause of most of these outbreaks.

Between December 2003 and mid-July 2006, A/H5N1 viruses have caused at least 230 confirmed cases (including 132 deaths) in human adults and children in Vietnam, Thailand, Indonesia,

China, Cambodia, Turkey, Iraq, Azerbaijan, Djibouti and Egypt. To date, people who have been infected with the A/H5N1 virus have been in direct contact with infected and sick birds. Until recently, the virus had not been infectious between humans, but a recent bird-flu outbreak in an Indonesian village has raised the level of concern that the virus may be able to pass directly between people since no animal has been identified as the source of the infection. This would be the first known three-person chain of human-to-human transmission.

There is a concern that the A/H5N1 virus, if given sufficient opportunity, will mutate into a form that is highly infectious and for which humans would have no natural immunity (that is, the population would have no antibody [substances that protect the body from infections] in the blood to protect against the virus). Such a virus could spread easily from one person to another, resulting in pandemic influenza.

Past influenza pandemics have been associated with the sudden onset of severe typical influenza symptoms such as headache, high fever, muscle and joint aches, reduced appetite, nausea, vomiting and cough, which typically last for 2 to 4 days. Some patients, after an initial recovery, subsequently develop pneumonia, and although most patients ultimately recover, some die rapidly due to breathing difficulties.

What vaccines or other treatments may be used to prevent or treat Pandemic Influenza?

In the event of a pandemic, it will be possible to use antiviral drugs, which are commonly used to treat influenza each year (for more information, ask your doctor), to control pandemic influenza and to treat those infected with the virus however the effectiveness of anti-viral drugs in the treatment of pandemic influenza has yet to be demonstrated. Vaccination may be effective at preventing pandemic influenza but as with antiviral drugs the use of an A/H5N1 vaccine in an A/H5N1 pandemic has to date not been demonstrated.

What is the purpose of this trial?

This is a medical research trial, the purpose of which is to test two influenza pandemic vaccine formulations as a two-primary dose schedule plus a booster vaccination in A/H5N1-naïve subjects (that is, subjects who have not previously been exposed to the virus contained in the vaccine) aged from 6 months to 17 years. Subjects will be studied in three age groups (9 to 17 years, 3 to 8 years, and 6 to 35 months). The influenza pandemic vaccine is produced from an avian influenza strain, specifically an A/H5N1 virus.

A similar influenza vaccine has already been injected in 300 naïve adults (aged 18 to 40 years) in a trial conducted in France, and in a further 300 naïve adults (aged 18 to 60 years) and 300 elderly subjects (aged >60 years) in a second trial in Belgium and England. Based on the results of the first trial, a 30 µg dose of vaccine with adjuvant (an adjuvant is a component of many vaccines, which is added to enhance the immune response) and 7.5 µg dose of vaccine without adjuvant were selected for use in the second trial. In the first trial, each vaccine formulation appeared to be generally safe and well tolerated and the highest antibody response was observed following vaccination with the 30 µg dose with adjuvant.

The objectives of the present trial are to describe the safety and the immune response following administration of two vaccinations (separated by 21 days) followed by a booster vaccination. The booster dose will be administered 6 or 12 months after the first vaccination. A final decision

regarding the time of administration of the booster (6 or 12 months) and its formulation will be made following a review of further data from the previous studies.

How will this trial be done?

This trial will consist of nine (design with booster at 6 months) or ten (design with booster at 12 months) visits (although the final 'visit' may be conducted by telephone) and will last 12 or 18 months, respectively. It will involve 60 subjects aged 9 to 17 years, 60 subjects aged 3 to 8 years, and 120 subjects aged 6 to 35 months. For subjects aged 3 to 8 years and 9 to 17 years, 30 subjects in each age group will receive each formulation (7.5 µg without adjuvant and 30 µg with adjuvant); for subjects aged 6 to 35 months, 30 subjects will receive each formulation and in addition 30 subjects will receive a half-dose of each formulation (ie 3.75 µg without adjuvant and 15 µg with adjuvant). The trial will be performed in Thailand.

At the start of the trial, your child will be assigned by chance to receive one of the selected formulations and will receive two injections of the same vaccine formulation, separated by an interval of 21 days, and a third (booster) vaccination at 6 or 12 months after the first vaccination.

At each visit, the Investigator will examine your child and will ask you questions about your child's health, and you will be asked to record any unwanted medical effects that could occur between these visits. In addition, a blood sample (7 mL [approximately 2 teaspoons] for those aged 3 to 17 years and 2 mL [approximately half a teaspoon] for those aged 6 to 35 months) will be taken from your child at each vaccination visit, at the visits prior to and 21 days after vaccination, and at 3 months after the first vaccination to measure the effect of the vaccinations on the amount of antibodies that your child develop.

Once all the results of the previous studies are available, the decision to administer a booster vaccination at either 6 or 12 months after the first vaccination, and the formulation to be used, will be made. Both options for a booster at 6 months or 12 months are presented below and you will be informed of the decision once this information is available.

- First visit:

All the risks and benefits associated with the trial will be explained to you before you sign the following informed consent statement. After having received satisfactory answers to any questions you may have, and if you agree for your child to take part in the trial, you will sign the informed consent form.

The Investigator will examine your child and check his/her medical history to make sure that he/she can take part in the trial. This involves a physical examination, a urine pregnancy test (if appropriate), and recording of your child's medical history (especially regarding any previous influenza-like illness, any previous influenza vaccination, any allergic reactions to previous vaccinations or any allergy to egg proteins). Parents/legal representatives will be asked not to allow your child to participate in another research trial for the whole period of the present trial.

If your child is eligible for the trial, 7 mL (approximately 2 teaspoons) of blood for those aged 3 to 17 years and 2 mL (approximately half a teaspoon of blood) for those aged 6 to 35 months will be taken in order to measure his/her level of antibodies before vaccination. An additional 2 mL (approximately half a teaspoon) of blood will be taken from subjects aged 6 to 35 months only, for further immunological analyses. Your child will then receive an injection of one of the two vaccine formulations into his/her upper-arm muscle (for those aged 1 to 17 years) or the thigh (for

those aged less than 1 year). To ensure your child's safety, he/she will stay at the trial center under medical observation for 30 minutes following vaccination.

You will then be given a digital thermometer, a ruler, and a diary card, together with instructions on their use, to note any unwanted effects that may happen to your child between vaccination and the next visit. On the evening of vaccination and on each of the next 7 days, you will be asked to record your child's axillary temperature and any specified symptoms commonly observed after any vaccine administration. You will also be asked to record any medications (other than the vaccine) that your child may take/be given during the 7 days following the vaccination. In addition, any non-specified symptoms occurring between the vaccination and the second visit should be recorded in the diary card.

- Second visit: 8 days after the first vaccination:

The Investigator will examine your child, review with you the diary card that you will have completed since the first vaccination and will question you on your child's health since vaccination.

- Third visit: 21 days after the first vaccination:

The Investigator will examine your child, review with you the diary card that you will have completed since the second visit and will question you on your child's health since the second visit. A urine pregnancy test will be performed if appropriate. A 7 mL (approximately 2 teaspoons) blood sample for those aged 3 to 17 years and 2 mL (approximately half a teaspoon) for those aged 6 to 35 months will be taken from your child to test the effect of the vaccine on his/her body's immune system. Then your child will receive the second vaccine injection into his/her upper-arm muscle or thigh (depending on age, as described earlier). To ensure your child's safety, he/she will stay at the trial center under medical observation for 30 minutes following vaccination.

You will then be given a second diary card to note any medications that your child may take/be given and unwanted effects that may happen between the second vaccination and the next visit.

- Fourth visit: 8 days after the second vaccination:

The Investigator will examine your child, review with you the diary card that you will have completed since the second vaccination and will question you on your child's health since vaccination.

A 2 mL (approximately half a teaspoon) blood sample will be taken from subjects aged 6 to 35 months only, for immunological analyses.

- Fifth visit: 21 days after the second vaccination:

The Investigator will examine your child, review with you the diary card that you will have completed since the previous visit and will question you on your child's health since the previous visit. A blood sample (7 mL [approximately 2 teaspoons] for those aged 3 to 17 years and 2 mL [approximately half a teaspoon] for those aged 6 to 35 months) will be taken in order to further investigate the effect of the vaccine on your child's body's immune system.

A memory aid will be provided to help you to record any important events until the next visit.

- Sixth visit: 3 months after the first vaccination:

The Investigator will examine your child, review with you the memory aid that you will have completed since the previous visit and will question you on your child's health since the previous visit. A blood sample (7 mL [approximately 2 teaspoons] for those aged 3 to 17 years and 2 mL [approximately half a teaspoon] for those aged 6 to 35 months) will be taken in order to further investigate the effect of the vaccine on your child's body's immune system.

Trial design with a 6-month booster design: seventh visit to ninth visit:

- Seventh visit: 6 months after the first vaccination:

The Investigator will examine your child, review with you the memory aid that you will have completed since the previous visit and will question you on your child's health since the previous visit. A urine pregnancy test will be performed if appropriate. A blood sample (7 mL [approximately 2 teaspoons] for those aged 3 to 17 years and 2 mL [approximately half a teaspoon] for those aged 6 to 35 months) will be taken from your child in order to further investigate the effect of the vaccine on his/her body's immune system. Then your child will receive the third vaccine dose (booster vaccination) into his/her upper-arm muscle or thigh (depending on age, as described earlier). To ensure your child's safety, he/she will stay at the trial center under medical observation for 30 minutes following vaccination.

You will then be given a third diary card to note any medications that your child may take and unwanted effects that may happen between the booster vaccination and the next visit.

- Eighth visit: 21 days after the booster vaccination:

The Investigator will examine your child, review with you the diary card that you will have completed since the previous visit and will question you on your child's health since the previous visit. A blood sample (7 mL [approximately 2 teaspoons] for those aged 3 to 17 years and 2 mL [approximately half a teaspoon] for those aged 6 to 35 months) will be taken from your child in order to further investigate the effect of the vaccine on his/her body's immune system.

A second memory aid will be provided to help you to record any important events until the next visit.

- Ninth visit (or telephone call): 12 months after the first vaccination:

The Investigator will examine your child (or interview you over the telephone), review with you the memory aid that you will have completed since the previous visit and will question you on your child's health since the previous visit. This visit will mark the end of your child's participation in the trial.

Trial design with a 12-month booster design: seventh visit to tenth visit:

- Seventh visit: 6 months after the first vaccination:

The Investigator will examine your child, review with you the memory aid that you will have completed since the previous visit and will question you on your child's health since the previous visit. A blood sample (7 mL [approximately 2 teaspoons] for those aged 3 to 17 years and 2 mL [approximately half a teaspoon] for those aged 6 to 35 months) will be taken from your child in order to further investigate the effect of the vaccine on your body's immune system.

A second memory aid will be provided to help you to record any important events until the next visit.

- Eighth visit: 12 months after the first vaccination:

The Investigator will examine your child, review with you the memory aid that you will have completed since the previous visit and will question you on your child's health since the previous visit. A urine pregnancy test will be performed if appropriate. A blood sample (7 mL [approximately 2 teaspoons] for those aged 3 to 17 years and 2 mL [approximately half a teaspoon] for those aged 6 to 35 months) will be taken from your child in order to further investigate the effect of the vaccine on your child's body's immune system. Then your child will receive the third vaccine dose (booster vaccination) into his/her upper-arm muscle or thigh (depending on age, as described earlier). To ensure your safety, your child will stay at the trial center under medical observation for 30 minutes following vaccination.

You will then be given a third diary card to note any medications that your child may take and unwanted effects that may happen between the booster vaccination and the next visit.

- Ninth visit: 21 days after the booster vaccination:

The Investigator will examine your child, review with you the diary card that you will have completed since the previous visit and will question you on your child's health since the previous visit. A blood sample (7 mL [approximately 2 teaspoons] for those aged 3 to 17 years and 2 mL [approximately half a teaspoon] for those aged 6 to 35 months) will be taken from your child in order to further investigate the effect of the vaccine on your child's body's immune system.

A third memory aid will be provided to help you to record any important events until the next visit.

- Tenth visit (or telephone call): 18 months after the first vaccination:

The Investigator will examine your child (or interview you over the telephone), review with you the memory aid that you will have completed since the previous visit and will question you on your child's health since the previous visit.

This visit will mark the end of your participation in the trial.

What are the risks and possible unwanted effects of vaccination and blood sampling?

Influenza vaccines are usually well tolerated when injected into a muscle. However, as with the administration of any other vaccine, your child may experience a variety of unwanted events at the injection site. The most frequently observed local reactions in a previous study were pain, erythema (red skin) and induration (a lump in the skin) and the most frequently reported solicited systemic reactions (general reactions) in all groups and for each injection were headache and muscle and joint pain. Your child may also feel unwell or experience high temperature (39°C to 40°C), or malaise. These effects usually disappear without treatment within 3 days. This is not a complete list of possible effects.

Since influenza vaccines have been marketed, some other reactions such as itching, urticaria, or redness over the entire body have been observed infrequently (between 1/100 and 1/1000 vaccinees). Nerve pain, paresthesia (feeling of "pins and needles"), convulsions, and temporary low blood platelet count have occurred rarely (between 1/1000 and 1/10,000 vaccinees). Very rare reactions like neurological disorders have been reported (in less than 1 out of 10,000 vaccinees). As with any vaccination, there is a rare possibility of an allergic reaction. This may cause a severe narrowing of the air passages and breathing difficulties. If this occurs, your child will need

immediate medical attention. This is why your child is required to remain under medical observation in the trial center for 30 minutes after each vaccination.

In addition, the sampling of blood from a vein may cause some pain, bruising at the puncture site, or lightheadedness.

There may be also other risks for your child or, if appropriate, for your daughter's unborn child, should she become pregnant during the trial, that are not yet known. If your child is pregnant at the moment, inform the Investigator. If your daughter becomes pregnant between the first visit and the booster visit, the Investigator will withdraw her from the trial but will follow her until the end of her pregnancy to monitor her health and that of her baby.

What are the possible benefits for your child?

The efficacy of the vaccine has not yet been established and there is no certainty that the influenza strain used in this vaccine will protect against a pandemic strain. As such, no benefit can be guaranteed.

What will you and/or your child have to do?

If you agree for your child to take part, or if you agree to take part in this trial, your child will have to attend the nine (design with booster at 6 months) or ten (design with booster at 12 months) planned visits. As described above, after each vaccination, you will be given a diary card to note any medical events or symptoms that may appear after vaccination until 21 days following vaccination, as well as any medication taken during that time. You will also have to measure your child's axillary temperature in the evening following each vaccination and for the seven subsequent days, and at any time that your child feel feverish.

You or a family member must inform the Investigator as soon as possible if a serious medical event occurs, e.g. if your child is admitted to hospital or visit an emergency department.

Sexually active female subjects will have to use an effective method of birth control for at least 4 weeks prior to and 4 weeks after each vaccination (including the booster vaccination at 6 or 12 months). You will have to inform the investigator as soon as possible should your daughter become pregnant. If your daughter plans to become pregnant during the next 12 or 18 months, she should not participate in the trial.

Who will see your child's medical and personal information?

Personal information collected during the trial will be confidential (with the exception of the situation described in the paragraph below); this confidential information will be computerized and sent to the Sponsor, and, if necessary, to authorized regulatory authorities. The personal information that will be collected includes medical data, date of birth, and age. You may exercise your right to access and correct your child's data. In these files, your child's name will be coded using his/her initials and a trial number. This is to protect your child's privacy. Your child's name and other information identifying you and/or your child will not be forwarded to the Sponsor.

In addition to the use of computerized information as described above, any personal records, including your child's medical records and health history, relating to this trial may be inspected in the Investigator's offices by authorized representatives of the Sponsor, or by the health authorities of any country where the trial vaccine may be considered for approval. Absolute confidentiality of your child's records can therefore not be guaranteed.

The results of this trial may be presented at meetings or in publications. Your child's identity will not be revealed in these presentations.

What happens if you refuse to let your child take part in the trial or change your mind after you agree?

Your decision regarding your child's participation in this trial is entirely voluntary. If you do not want your child to take part, then you do not have to agree to his/her participation. You also have the right to agree to your child's participation and subsequently to change your mind. You can withdraw your child from the trial at any time. If you withdraw your child from the trial, the blood samples collected before your child's withdrawal will be used unless if you specifically request otherwise.

Whatever your decision, your child will continue to qualify for full medical care without any penalty or loss of benefits to which your child is otherwise entitled.

The Investigator or Sponsor may withdraw your child from this trial for any reason at any time even without your consent, for example if the Investigator decides that it is in your child's best interest, or if you do not follow the trial instructions.

If you do decide to withdraw your child from the trial, or if your child is withdrawn by the doctor, then the Investigator may recommend that certain laboratory tests or physical examinations should be done to ensure your child's safety and well-being.

What else do you need to know before agreeing to let your child to take part in this trial?

Taking part in this trial is entirely voluntary. You will be provided with any new relevant information during the trial that may affect your willingness to continue your child's participation.

The investigator will inform your child's general practitioner about his/her participation in this trial unless you do not consent to this.

This trial has been designed and will be carried out in accordance with the ICH/GCP guidelines laid down in the Declaration of Helsinki for the protection of individuals participating in clinical trials.

You will receive a copy of this signed Informed Consent Form. Please keep it safe and use it for information and reference throughout the trial.

If your child is ill or injured during the study and it is determined that this was due to the vaccination, you will be compensated for all reasonable outpatient costs of medical evaluation and treatment or hospitalization that are directly related to this illness or injury. In any case, and whatever the cause of the illness or injury, your child will qualify for the usual standard of medical care that he/she would have received had he/she not participated in this trial.

The Sponsor has an insurance policy covering study-related illness or injury.

You will be re-imbursed for travel expenses associated with your child's participation in this trial.

Do not sign this Informed Consent Form or agree to your child's participation in this trial unless you are comfortable with the risks involved and you have had an opportunity to ask questions and feel you have received satisfactory answers.

What will happen to your child's blood samples?

As a part of this trial, blood samples will be taken to test the effect of the pandemic influenza vaccine on your child's body's immune system. These tests will be done by the Sponsor or by another laboratory for the Sponsor. Your child's samples will be shipped out of Thailand for these tests and will be stored in a freezer until these tests are done. Therefore, you should only agree to participation in this trial if you agree to this use of your child's blood samples.

Any unused part of your child's blood samples will be securely stored by the Sponsor in the USA. They will be stored until at least 10 years after the vaccine being tested has been approved for use by the health authorities.

What should you know about possible future use of your samples for research?

The Sponsor would also like to keep any unused part of your child's blood samples for use in other evaluation studies, e.g. studies of the immune response to influenza vaccines, improvement of knowledge and documentation of the safety of this vaccine by using new developed laboratory methods or applying newly discovered concepts. You will not be informed about the types of research that will be conducted or any of the results, although, genetic tests will never be performed on these samples.

You have the right to agree to participation in the trial proposed to you today and still refuse to allow the Sponsor keep any unused blood samples for future use. You will be asked to decide whether or not you permit this future use of your child's blood samples and to indicate your decision on the consent form.

You will not be paid for allowing the Sponsor to keep and later use any remaining part of your child's blood samples.

Should you refuse to allow the Sponsor to keep your child's unused blood samples then they will not be used for any testing other than that directly related to this trial.

Who can you contact if you have questions?

If you have any questions about this trial or if you want more information on compensation or medical treatment in the case of trial injuries, please contact:

Name: ……………….

Tel. Number: …………………..

If you have any questions about your rights as a subject in this trial, please contact:

Name: ……………….

Tel. Number: …………………..

Informed consent statement for participating in the trial

Subject's Last and First Names: ____

Subject's Entry Number: |0|0|-|0|0| | | | | Subject's Initials: |__| |__| |__|

By signing this form I certify to all of the following:

- I have read this entire Informed Consent Form (or had the information read to me) and understand what will be done to my child.
- I consent to let my child take part to take part in this trial.
- I consent to make my child's confidential personal information available for review (direct access) to the Sponsor's representative or to any Competent Authorities, Institutions or governmental agencies assigned this task in this country or in another country where the trial vaccine may be considered for approval, or if applicable, the Ethics Committee/Institutional Review Board.
- I authorize the Investigator and Sponsor to have access to the medical records if my child is admitted to a hospital or visit an emergency ward for any reason during the trial period.
- I understand and accept that information related to my child/me collected during the trial will be coded so that my child's name does not appear in my child's record that is computerized and shared with the Sponsor. I am aware that my child's computerized information may be handled in another country. I am aware that I may exercise my right of access and correction of these data at any time with the Investigator.
- I have had the opportunity to ask questions and have received satisfactory answers, and I understand that I will receive a signed copy of this Informed Consent Form.
- I understand that I may ask additional questions about this trial at any time.
- I understand that I am free to withdraw my child from the trial at any time without justifying my decision to do so and without it affecting my child's medical care.
- I understand that if I withdraw my consent, my child's blood samples collected before my child's withdrawal will be used unless I specifically request otherwise.
- I authorize the shipment of my child's blood samples to the Sponsor's laboratory (or other location as applicable) for doing tests related to this trial, and I understand that the laboratory may be in another country.

Specific consent statement for the future use of your child's blood samples in research

Please indicate your choice by checking one of the two boxes below:

☐ I authorize the future use of my child's/my blood samples for future research by the Sponsor under the conditions described in this information sheet (see the paragraph called "*What should you know about possible future use of your child's* blood samples*for research*").

OR

☐ I authorize the use of my child's/my blood samples for doing tests related to this trial *BUT DO NOT* authorize the future use of my child's/my blood samples for any other research.

Last and first name of parent/legally authorized representative

Signature of parent/legally authorized representative	Relationship	Date

Last and first name of witness
(only required if parent/legally authorized representative is illiterate)

Signature of witness (only required if parent/legally authorized representative is illiterate)	Relationship	Date

Investigator's statement (or person performing the Informed Consent procedure)

I certify that I have explained to the above individual the nature and purpose of the trial, potential benefits, and reasonably foreseeable risks associated with participation in this research trial. I have answered any questions that have been raised and have witnessed the above signature. I have explained the trial, as described in the informed consent form, to the parent/legally authorized representative of the child/the child whose name is listed above on the date stated on this consent form.

____

Last and first name and signature (of the person performing the Informed Consent procedure) — Date

______________________________________ ____________

Signature of Investigator (if different from the above and if required by local regulations) — Date

Protocol Appendix 3: Signature Page

Signatures

I have read and agree to conduct this trial according to the procedures outlined in this protocol (Protocol version 5.0 dated 21 May 2007) and in accordance with applicable regulations and Good Clinical Practice.

Function	Name	Date	Signature
**Investigators**	T. CHOTPITAYASUNONDH<br><br>U. THISYAKORN	23 May 2007<br><br>23 May 2007	[signature]<br><br>Usa Thisyakorn
**Sponsor's Responsible Medical Officer**<br>Clinical Team Leader<br>sanofi pasteur France	M. SAVILLE	21 May 2007	[signature]
**Biostatistician**<br>sanofi pasteur France	C. CARRÉ	21 MAY 2007	[signature]
